# Unprecedented Formation of a Formally Cu(III) Trifluoromethyl Hydroxide Tetramer

**DOI:** 10.1002/chem.202503417

**Published:** 2025-12-18

**Authors:** Vladimir Motornov, Niklas Limberg

**Affiliations:** ^1^ Freie Universität Berlin Berlin Germany

**Keywords:** copper, fluorine, high‐valent copper, oxidation, oxygen

## Abstract

Aerobic copper‐mediated oxidative processes play a pivotal role in the context of enzymatic oxidation and catalysis. Herein, the first high‐valent copper(III) trifluoromethyl hydroxide with the tetrameric heterocubane structure [Cu(CF_3_)_2_(OH)]_4_ was synthesized by air oxidation of copper(I) in the presence of TMSCF_3_/KF and fully characterized, including X‐ray crystallography. This unique compound displays versatile reactivity, functioning as a hydroxide base in neutralization reactions with acids, which affords a broad variety of high‐valent Cu(III) complexes with two trifluoromethyl groups. The synthetic relevance of copper(III) trifluoromethyl hydroxide for boronic acid trifluoromethylation was demonstrated. The natural population analysis (NPA) charge distribution in the tetrameric heterocubane was studied by DFT calculations, which support the experimentally observed behavior of this compound as a weak hydroxide base, and revealed the positive NPA charge of +1.157 on high‐valent copper.

## Graphical abstract

Novel high‐valent Cu(III) trifluoromethyl hydroxide scaffold with an unprecedented heterocubane structure was synthesized. Its neutralization reactions with acids afford a broad variety of Cu(III) complexes with two trifluoromethyl groups. Synthetic relevance of this compound for C─CF_3_ bond formation was demonstrated.

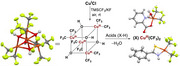

## 1 Introduction

**FIGURE 1:**
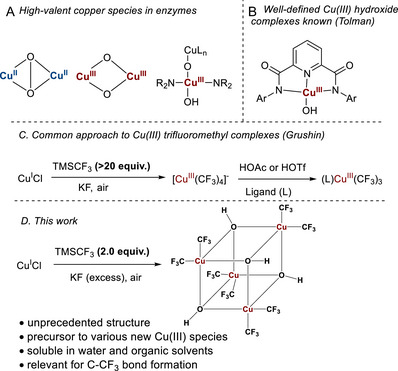
Copper(III) hydroxide and trifluoromethyl complexes and the present work.

Copper(III) complexes are key intermediates in aerobic oxidation, crucial for understanding the mechanisms in copper‐containing enzymes, in addition to oxidative processes in synthetic chemistry [1, 2, 3, 4, 5]. Elusive high‐valent copper‐dioxygen, copper‐peroxo, and hydroxide species (Figure 1A) are products of oxygen activation by Cu(I) and are most commonly represented in biological systems [6, 7, 8, 9, 10, 11]. From an inorganic chemistry perspective, the oxidation state +3 of copper is extremely rare in well‐defined complexes [12, 13]. All known inorganic derivatives of high‐valent copper, as well as Cu(III) hydroxide species stabilized by organic ligands [6, 7, 8, 9, 10, 11] (Figure 1B), exhibit strong oxidative properties. The chemistry of Cu(III) trifluoromethyl complexes represents a significant, yet underexplored field within the realms of organometallic and coordination chemistry, which has witnessed a significant increase in scientific interest over the past two decades [14, 15, 16, 17, 18]. Trifluoromethyl group is known to stabilize high oxidation states [14, 15, 16, 17, 18], including Cu(III), Ni(IV), and Co(III), due to its electron‐withdrawing nature and a high degree of covalency in the M─CF_3_ bonds [19, 20, 21, 22]. While the first Cu(III) trifluoromethyl species were prepared a while ago,[23, 24, 25] a major advancement in this area occurred in 2015, when Grushin and co‐workers developed a straightforward method to prepare tetrakis(trifluoromethyl)cuprate salts, Q^+^[Cu(CF_3_)_4_]^−^, from inexpensive copper(I) chloride and TMSCF_3_ using only air as the oxidant (Figures 1C) [25]. This discovery opened the door to the broader application of neutral Cu(III) trifluoromethyl complexes as reagents in synthetic organic chemistry, such as arene, alkene, and alkyne trifluoromethylation [26, 27, 28, 29, 30]. In the recent five years, new trifluoromethyl complexes, such as weakly ligated Cu(CF_3_)_3_ solvates [31], 1,3‐diketonates [32], Cu(III) halide derivatives [33, 34, 35], pyridine‐2,6‐dicarboxamide [34, 36] complexes were prepared by us and other researchers. However, no hydroxide or oxo‐complexes of Cu(III) have been stabilized by trifluoromethyl group to date. The formation of Cu(III) complexes directly by air oxidation of Cu(I) is a fundamental step in crucial transformations such as Cu(I)‐mediated oxidative couplings, employing air or oxygen as cost‐efficient oxidants [37, 38, 39]. However, the identity of such species in trifluoromethylation still remains unknown, since [Cu(CF_3_)_4_]^−^ is poorly reactive in CF_3_ group transfer [26, 40]. Studying Cu(III) oxide and hydroxide species formed by oxygen oxidation of Cu(I) in the stabilized CF_3_‐ligand environment is therefore of special importance for understanding aerobic copper‐mediated processes both in biological systems and catalysis applications. Herein, we report the formation of the unique tetrameric copper(III) hydroxide with a distorted heterocubane structure, along with its reactivity as a base to access novel Cu(III) complexes (Figure 1D).

Inspired by the simplicity of the original Grushin's synthesis of [Cu(CF_3_)_4_]^−^ anion [25], along with the importance of Cu(I) aerobic oxidation for biological systems [1, 2, 3, 4, 5, 6, 7, 8, 9, 10, 11], we attempted to perform oxidation of CF_3_‐coordinated Cu(I) species by air. Notably, a large excess of TMSCF_3_ has always been used in this synthesis to facilitate the efficient formation of [Cu(CF_3_)_4_]^−^ anion [25, 31]. However, the outcome of this oxidation process with a lower Cu:CF_3_ ratio is unclear and has not been studied. Modifying the Grushin's synthesis to change the chemoselectivity and unlock the routes to other Cu(III) species apart from the extremely stable homoleptic anion is a challenging task.

## 2 Results and Discussion

**SCHEME 1:**
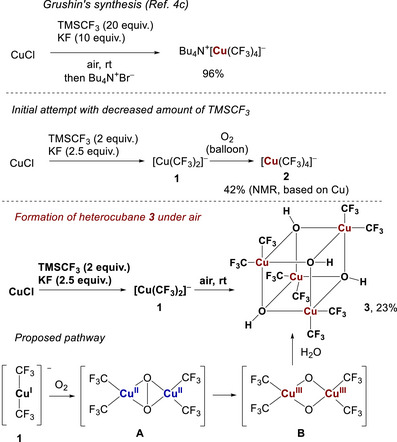
Unprecedented formation of heterocubane **3** compared to the original synthesis of cuprate salt **2**.

We initiated our studies with the oxidation of pre‐formed bis(trifluoromethyl)cuprate(I) under an oxygen atmosphere in the absence of additional TMSCF_3_ (Scheme 1). However, only the formation of [Cu(CF_3_)_4_]^−^ in 42% yield was observed by ^19^F NMR. Thus, no target species with two trifluoromethyl groups was detected, which can likely be explained by transmetallation of the intermediate species with an excess of [Cu(CF_3_)_2_]^−^. To our surprise, replacing oxygen with air resulted in the formation of a new compound with a resonance of −26.3 ppm in ^19^F NMR in CDCl_3_, with two magnetically equivalent CF_3_ groups. The compound could be easily isolated via column chromatography as a slightly yellow powder, soluble in water, acetonitrile, acetone, and chloroform. ^1^H NMR of the pure complex featured a single broad singlet at 1.16 ppm, while the infrared (IR) spectrum revealed a characteristic band at 3636 cm^−1^ indicating the presence of an OH group. Thus, both oxygen and water vapors are necessary for the synthesis of this copper(III) hydroxide. Upon exposure of the reaction mixture to air, it turned dark, which can indicate the formation of intermediate Cu(II) species. Copper‐oxo intermediates such as (μ‐η^2^:η^2^‐peroxo)dicopper(II) (**A**) or bis(μ‐oxo)dicopper(III) (**B**) may be involved in the process, which undergo hydrolysis to the Cu(III) hydroxide **3**. Most likely, an excess of KF and water vapors is responsible for tuning the chemoselectivity of the process, since the basic medium is known to induce the formation of Cu(III) species **B** from Cu(II)‐dioxygen species **A** [7]. In the case of Grushin's process **B** may undergo transmetallation with another equivalent of [Cu(CF_3_)_2_]^−^ to form [Cu(CF_3_)_4_]^−^ subsequent to further oxidation.

**FIGURE 2:**
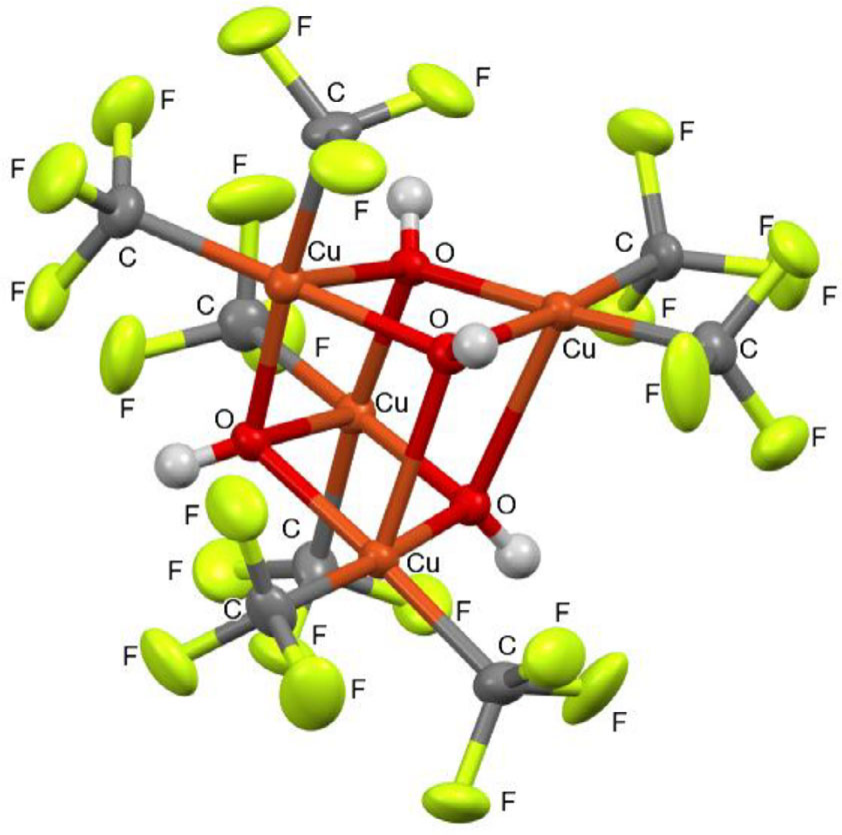
X‐ray structure of copper(III) hydroxide heterocubane **3**. CCDC 2481836. Thermal ellipsoids are shown at a 50% probability level.

X‐ray crystallography confirmed an unprecedented tetrameric, distorted heterocubane structure, matching the formula [Cu(CF_3_)_2_(OH)]_4_, with copper and oxygen atoms of the bridge μ_3_‐OH groups forming the scaffold (Figure 2). The three symmetrically independent torsion angles between two adjacent Cu─O axes are 10.8°, −8.5°, and 14.1°, respectively, which makes the scaffold rather unsymmetrical. Copper(III) centers adopt a square‐pyramidal geometry with a coordination number of five, which is characteristic of Cu(III) trifluoromethyl compounds. Two Cu─OH distances represent strong coordination (Cu─O = 1.90‐1.94 Å), whereas one Cu─OH in apical position to each copper atom is weakly coordinated (Cu─O = 2.39‐2.41 Å) (Figure 2). The preference for high coordination numbers in Cu(III) complexes in the presence of trifluoromethyl groups, together with the strong Lewis acidity of Cu(III), could explain the tetrameric structure of **3**, compared to a hypothetical coordinatively unsaturated monomer Cu(CF_3_)_2_(OH).

The product is stable at room temperature for several hours, and for months at −40°C. The developed method is a one‐step procedure that is exceedingly simple to operate and uses oxygen as the most cost‐efficient oxidant. While the chemical yield of **3,** based on copper, is not high (23%), this is completely reasonable, considering the unique properties of this copper(III) hydroxide scaffold and the fact that TMSCF_3_, more expensive than a copper salt, is used in a stoichiometric quantity.

**SCHEME 2:**
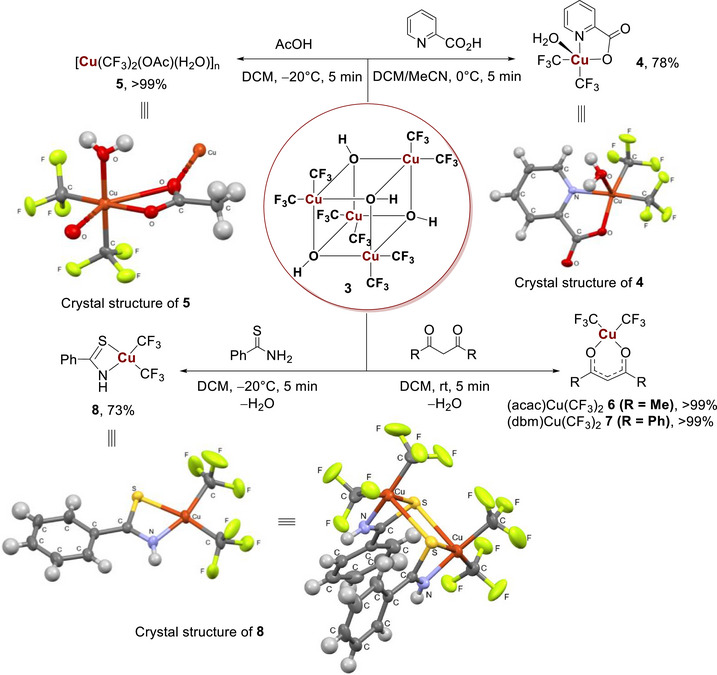
Transformations of heterocubane **3**. CCDC 2481839 (for **4**), 2481840 (for **5**), and 2481838 (for **8**). Thermal ellipsoids are shown at 50% probability level.

With the confirmed structure of the first Cu(III) heterocubane, we investigated the transformations of this unique compound. First, due to one relatively long Cu─OH bond, a quite labile character of the coordination can be expected, which could result in facile ligand exchange with the breakdown of the heterocubane scaffold. Second, compound **3** can be considered a formal Cu(III) hydroxide and thus expected to exhibit basic properties. Accordingly, we tested its reactivity with Brønsted acids (Scheme 2). Reaction with coordinating pyridine‐2‐carboxylic acid resulted in the formation of complex (2‐PyCOO)Cu(CF_3_)_2_(H_2_O) **4**, isolated as bright yellow crystals. X‐ray analysis confirmed a structure with a square‐pyramidal Cu(III) center (*τ*_5_ = 0.194), which turned out to be a monohydrate with a weakly coordinated water molecule at the apical position. In contrast to coordinating pyridine‐2‐carboxylic acid, the reaction of **3** with acetic acid at −20°C afforded the significantly less stable acetate complex [Cu(CF_3_)_2_(OAc)(H_2_O)]_n_ **5** in quantitative yield. X‐ray analysis revealed a polymeric structure, where each copper atom is coordinated by two acetate ligands with additionally coordinated water molecules.

With the reactivity of hydroxide **3** with carboxylic acids being examined, we tested weaker acidic agents as reaction partners. Compound **3** reacted quantitatively with 1,3‐diketones, such as acetylacetone (Hacac) and dibenzoylmethane (Hdbm), with neutralization and elimination of a water molecule, to give synthetically useful [32] complexes (acac)Cu(CF_3_)_2_ **6** and (dbm)Cu(CF_3_)_2_ **7,** respectively, in quantitative yield. The synthesis is quick and straightforward due to the fast ligand exchange in Cu(III) trifluoromethyl species, offering an alternative to AgF oxidation [32].

In contrast to the stabilization by nitrogen‐ and oxygen‐donor ligands, reaction with thiobenzamide at reduced temperature resulted in the formation of an unstable complex **8**, precipitated as orange crystals, in which bidentate (S, N) η_2_‐coordination of the ligand is realized. In the solid state, the compound **8** adopts a dimeric structure comprising a four‐membered ring with two Cu and two S atoms.

**SCHEME 3:**
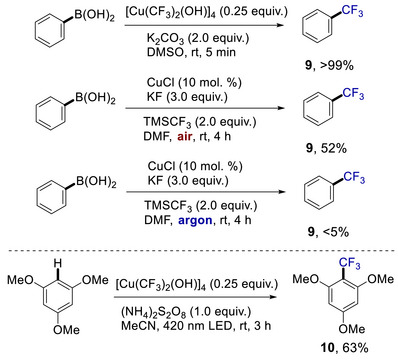
Applications of copper(III) trifluoromethyl hydroxide **3** in C─CF_3_ bond formation reaction.

With the versatile reactivity of **3** in ligand exchange reactions established, we turned our attention to demonstrate the relevance of this unique high‐valent copper species to trifluoromethylation reactions (Scheme 3). Thus, transmetallation of **3** with boronic acid proceeds smoothly in mild conditions to quantitatively afford the corresponding trifluoromethylarene **9** within 5 min. This provides evidence for the participation of copper(III) hydroxide **3** in aerobic copper‐catalyzed coupling reactions [37, 38, 39]. Formation of the trifluoromethylation product was also observed under catalytic conditions with CuCl under air, which did not take place under an inert atmosphere. While a similar reactivity was observed by Grushin and coworkers in the presence of the ligand [40], we believe the elusive Cu(III)‐oxo complex (Scheme 1B) might be the catalytically active species, which forms the title compound **3** after hydrolysis. In addition to catalytically relevant reactivity in boronic acid trifluoromethylation, C−H trifluoromethylation of an electron‐rich arene (trimethoxybenzene) under 420 nm LED irradiation smoothly afforded mono‐trifluoromethylation product **10**.

**FIGURE 3:**
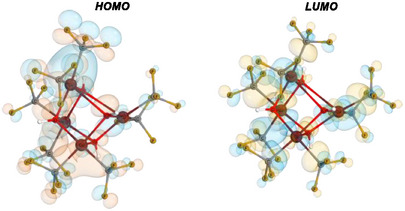
Frontier orbitals of the title compound **3** [Cu(CF_3_)_2_(OH)]_4_ calculated on ωB97X‐D3(BJ)/def2‐QZVPPD//B3LYP/ZORA‐def2‐TZVP level of theory.

Having investigated the reactivity of the heterocubane **3**, we studied the electronic structure of this compound by DFT calculations. The frontier orbitals of **3** were calculated on ωB97X‐D3(BJ)/def2‐QZVPPD//B3LYP/ZORA‐def2‐TZVP level of theory. The calculated LUMO is predominantly metal‐centered, especially along the Cu─C σ‐bonds, while the HOMO has a predominantly Cu─O‐centered character (Figure 3 ). This HOMO differs from the substantially ligand‐based HOMO in the classical inverted ligand field species [Cu(CF_3_)_4_]^−^ [19, 20, 21, 22, 25], as well as (DMF)_2_Cu(CF_3_)_3_ [31], while the significant electron density along the Cu─C bond supports the strong covalent bonding suggested for high‐valent copper trifluoromethyl species.

**FIGURE 4:**
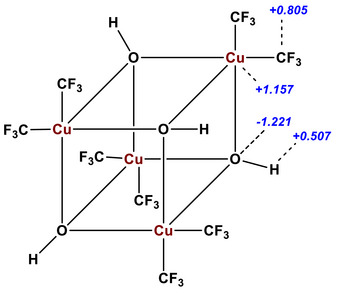
Calculated NPA charges (blue numbers) for the compound **3** [Cu(CF_3_)_2_(OH)]_4_ on ωB97X‐D3(BJ)/def2‐QZVPPD//B3LYP/ZORA‐def2‐TZVP level of theory.

The analysis of the natural charges (NPA) (Figure 4) revealed a relatively low positive charge on the high‐valent copper (+1.157), which is nevertheless considerably higher than for complexes with three or four trifluoromethyl groups (+0.19 [25] for [Cu(CF_3_)_4_]^−^ and +0.8 for Cu(CF_3_)_3_ species [31]. The positive charge of the formally trivalent copper is therefore distributed between copper and two adjacent carbon atoms (+0.805). Polarization of the Cu─OH bonds supports the experimentally observed reactivity of **3** as a weak hydroxide base.

## 3 Conclusion

In conclusion, a new high‐valent Cu(III) organometallic scaffold with a unique heterocubane structure was synthesized. Its remarkably versatile reactivity as a hydroxide base with carboxylic acids, 1,3‐diketones, and thioamide gives rise to a variety of Cu(III) complexes with two trifluoromethyl groups. High synthetic relevance of the copper(III) hydroxide tetramer for C─CF_3_ bond formation was demonstrated for boronic acid trifluoromethylation. Further in‐depth studies of reactivity and applications of tetrameric Cu(III) hydroxide are currently underway.

## Conflicts of Interest

The authors declare no conflict of interest.

## Supporting information




**Supporting file 1**: The authors have cited additional references within the Supporting Information [41, 42, 43, 44, 45, 46, 47, 48, 49, 50, 51, 52, 53, 54, 55, 56]. Deposition Numbers 2481836 (for **3**), 2481839 (for **4**), 2481840 (for **5**), 2481838 (for **8**) contain the supplementary crystallographic data for this paper. These data are provided free of charge by the joint Cambridge Crystallographic Data Centre and Fachinformationszentrum Karlsruhe Access Structures service (https://www.ccdc.cam.ac.uk/structures).

**Supporting file 2**: chem70587‐sup‐0002‐SuppMat.zip

**Supporting file 3**: chem70587‐sup‐0003‐SuppMat.zip
