# Supplementary material for: Unprecedented Formation of a Formally Cu(III) Trifluoromethyl Hydroxide Tetramer
Source: Chemistry. 2025 Dec 18;32(5):e03417. doi: 10.1002/chem.202503417 (PMC12865140; doi:10.1002/chem.202503417)

Supplementary information for

Unprecedented Formation of a Cu(III) Trifluoromethyl Hydroxide Tetramer

Vladimir Motornov,[a],* and Niklas Limberg [a]

[a] Dr. V. Motornov, N. Limberg, Freie Universität Berlin; Fabeckstraße 34-36, 14195 Berlin, Germany.
E-mail: cuprate51@gmail.com, motornov@zedat.fu-berlin.de

Table of contents

General 1
General procedure for the synthesis of heterocubane 3. 2
Synthesis of complex 4 with pyridine-2-carboxylic acid 2
Synthesis of (acetato)bis(trifluoromethyl)aquacopper(III) 5 3
Synthesis of 1,3-diketonate complexes 6-7 3
Synthesis of thiobenzamide derivative 8 4
Synthetic applications of Cu(III) trifluoromethyl hydroxide 4
X-ray crystallography 7
DFT calculations 22
References 24
Author contributions 24
NMR spectra 25

General

All solvents used for the reactions were HPLC grade. All commercially available chemicals were purchased from commercial suppliers and were used as received. KF was dried under high vacuum at 120°C for 4 hours before use and stored under argon. Copper(I) chloride was purified by dissolution in concentrated HCl and stored under argon (should be a **white** powder). Yields refer to isolated compounds, estimated to be >95% pure as determined by ^{1}H NMR. Column chromatography was performed using silica gel 60 (40–63 µm) from Merck. NMR spectra were

recorded on a JEOL 400 MHz spectrometer in the solvent indicated; chemical shifts (δ) are given in ppm relative to the residual solvent peak. ^{1}H, ^{13}C and ^{19}F NMR spectra were measured at ambient temperature using 5 mm diameter NMR tubes. ^{13}C NMR spectra were proton decoupled. The chemical shift values (*δ*) are reported in ppm relative to Me_4Si (0 ppm for ^{1}H, ^{13}C NMR) or $CFCl_3$ (0 ppm for ^{19}F NMR). Coupling constants (*J*) are reported in Hertz.

Synthetic procedure for the synthesis of heterocubane 3.

In a 100 ml Schlenk flask under argon atmosphere, to the solid mixture of CuCl (495 mg, 5 mmol) and dry KF (696 mg, 12 mmol, 2.4 equiv.) dry DMF (10 ml) was added and the resulting mixture was stirred for 5 min until a bright red solution of solvated Cu(I) fluoride species is obtained. Then it was cooled to −30 °C and $TMSCF_3$ (1.51 ml, 10 mmol) was added dropwise via syringe. The mixture was stirred for 1-2 h at −30 to −10 °C. After the red color of the reaction mixture ceased (which indicates complete formation of $[Cu(CF_3)_2]^-$), the mixture was warmed up to room temperature, exposed to air, and stirred intensively overnight. The resulting dark mixture was quenched by CH_2Cl_2 (100 ml) and H_2O (50 ml). After intensive stirring for 15 min the dark color changed to yellow in the organic phase. Organic layer was separated; the aqueous layer was washed twice with CH_2Cl_2. The combined organic layer was dried over anhydrous Na_2SO_4, evaporated (heating above 30°C should be avoided), and the residual DMF was removed on a high vacuum pump equipped with a liquid nitrogen trap. The yellow residue was purified by column chromatography on silica gel (1:4 pentane/CH_2Cl_2 to CH_2Cl_2) to give **3** (251 mg, 23 %) as a pale-yellow solid.

X-ray quality colorless crystals of **3** were obtained by slow evaporation from the DCM solution at 0-5°C (refrigerator) for 3 days.

^{1}H NMR (400 MHz, MeCN-*d*$_3$) δ 1.16 (br s); **^{19}F NMR** (376 MHz, MeCN-*d*$_3$) δ −30.3 (br s). **^{19}F NMR** (376 MHz, $CDCl_3$) δ −26.3 (s). **^{19}F NMR** (376 MHz, acetone-*d*$_6$) δ −29.8 (br s). **^{19}F NMR** (376 MHz, D_2O) δ −32.1 (br s). **IR** (ATR): $\tilde{\nu}$ = 3636, 1651, 1105, 953, 709 cm^{-1}; **CCDC** 2481836.

Synthesis of complex 4 with pyridine-2-carboxylic acid

To the solution of heterocubane **3** (21.8 mg, 0.025 mmol [0.1 mmol monomer]) in DCM (0.7 ml) a solution of pyridine-2-carboxylic acid (1.0 equiv.) in 0.3 ml of MeCN was added dropwise at 0°C (ice bath) with vigorous stirring. After 5 min, the stirring was stopped, the mixture was layered with pentane (3 ml) and

placed into the freezer at −40°C. After 3 days bright yellow crystals formed, which were collected and dried in vacuo. Yield of complex **4**: 25 mg (78%). X-ray was measured for a crystal before drying, which contains a weakly coordinated water molecule.

Bis(trifluoromethyl)(aqua)copper(III) pyridine-2-carboxylate **4**

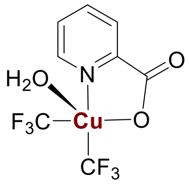

^{1}H NMR (400 MHz, MeCN-d_3) δ 8.52 (d, J = 5.7 Hz, 1H), 8.25 (t, J = 7.6 Hz, 1H), 8.09 (d, J = 7.8 Hz, 1H), 7.79 (t, J = 6.8 Hz, 1H). **^{13}C NMR** (101 MHz, MeCN-d_3) δ 153.0, 148.9, 143.3, 129.8, 127.1. **^{19}F NMR** (376 MHz, MeCN-d_3) δ −29.3 (s). **IR** (ATR): $\tilde{\nu}$ = 1675, 1639, 1610, 1368, 1151, 1123, 1115, 1095, 771, 714 cm^{-1}; **CCDC** 2481839.

Synthesis of (acetato)bis(trifluoromethyl)aquacopper(III) 5

To the solution of heterocubane **3** (21.8 mg, 0.025 mmol [0.1 mmol monomer]) in DCM (0.5 ml) at −20°C acetic acid (5.7 µl, 6.0 mg, 0.1 mmol) was added in one portion with stirring. After 5 min at −20°C, the flask was connected to the high vacuum and the solvent was evaporated to dryness to give **5** (28 mg, >99% based on monohydrate) as a yellow powder.

^{1}H NMR (400 MHz, $CDCl_3$) δ 2.68 (br s, Me), 2.2-2.0 (br, H_2O) ppm; **^{19}F NMR** (376 MHz, $CDCl_3$) δ −30.4 (br s). **HRMS** (ESI) *m/z* calcd for $[C_4H_4{}^{65}CuF_6O_3]^-$: 278.9348 [M-H]$^-$, found 278.9356. **IR** (ATR): $\tilde{\nu}$ = 2306, 2279, 1657, 1565, 1522, 1442, 1103, 937, 722, 704 cm^{-1}; **CCDC** 2481840.

X-ray quality crystals of a hydrate **$5{\cdot}2H_2O$** were obtained after recrystallization of the resulting DCM solution after layering with pentane at −40°C for 3 days. The compound is temperature-sensitive and decomposes at ambient temperature in solution within a few hours with the release of CF_3 radical.

Synthesis of 1,3-diketonate complexes 6-7

To the solution of heterocubane **3** (21.8 mg, 0.025 mmol [0.1 mmol monomer]) in DCM (1 ml) acetylacetone (10.2 µl, 10 mg, 0.1 mmol, for **6**) or a solution of dibenzoylmethane (22.4 mg, 0.1 mmol, for **7**) in 0.5 ml DCM was added at room temperature. The mixture was stirred for 5 min, then the solvent was evaporated to dryness to give pure diketonate complexes **6** and **7**.

(Acetylacetonato)bis(trifluoromethyl)copper(III) **6**

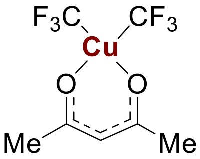

Yield 30 mg (quant.), orange solid. **^{1}H NMR** (400 MHz, $CDCl_3$) δ 5.65 (s, 1H), 2.11 (s, 6H); **^{13}C NMR** (101 MHz, $CDCl_3$) δ 189.6 (C=O), 101.8 (=CH), 26.8 (Me); **^{19}F NMR** (376 MHz, $CDCl_3$) δ −30.2 (s); NMR matched previously reported data.[1]

(1,3-Diphenyl-1,3-propandionato)bis(trifluoromethyl)copper(III) **7**

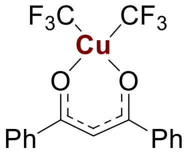

Yield 42.5 mg (quant.), red solid. **^{1}H NMR** (400 MHz, $CDCl_3$) δ 8.00 (d, J = 7.6 Hz, 4H), 7.60-7.58 (m, 2H), 7.51-7.49 (m, 4H), 7.01 (s, 1H); **^{13}C NMR** (101 MHz, $CDCl_3$) δ 183.0 (C=O), 136.9, 133.0, 129.0, 128.2, 94.9 (=CH); **^{19}F NMR** (376 MHz, $CDCl_3$) δ −30.0 (s); NMR matched previously reported data.[1]

Synthesis of thiobenzamide derivative 8

To the solution of heterocubane **3** (11 mg, 0.0125 mmol [0.05 mmol monomer]) in DCM (0.5 ml) at −20°C a solution of thioacetamide (3.8 mg, 0.05 mmol) in 0.5 ml of DCM was added dropwise with vigorous stirring. After 5 min of stirring at −20°C, the mixture was layered with pentane (4 ml) and left in the freezer (−40°C) over the weekend for crystallization. Bright orange-red crystals of **8** (17 mg, 73%) were collected and dried in vacuo. The compound is temperature-sensitive and decomposes at ambient conditions in dry form within a day and in a chloroform solution within 30 minutes.

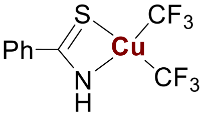

(η^2-Thiobenzamido)bis(trifluoromethyl)copper(III) 8

^{1}H NMR (400 MHz, $CDCl_3$) δ 8.96 (br s, 1H, NH), 7.78 (d, J = 7.8 Hz, 2H), 7.70 (t, J = 7.4 Hz, 1H), 7.60 – 7.52 (m, 2H) ppm; **^{13}C NMR** (101 MHz, $CDCl_3$) δ 139.4, 135.4, 129.3, 125.8 ppm; **^{19}F NMR** (376 MHz, $CDCl_3$) δ −22.4 (br s, 3F), −30.8 (br s, 3F); **HRMS** (ESI) m/z calcd for $[C_9H_5CuF_6NS]^-$: 335.9348 $[M-H]^-$, found 335.9360. **CCDC** 2481838.

Synthetic applications of Cu(III) trifluoromethyl hydroxide

Trifluoromethylation of phenylboronic acid

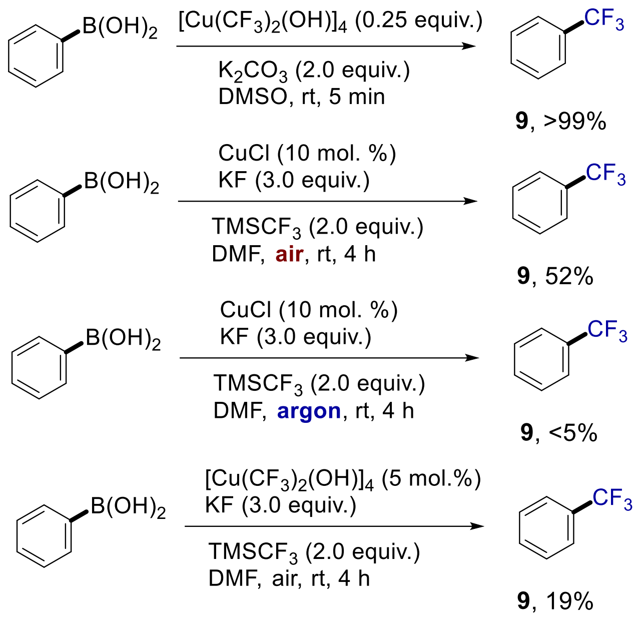

Experiment 1. Stoichiometric trifluoromethylation. A 15 ml Schlenk tube charged with **3** (21.8 mg, 0.1 mmol based on monomer), potassium carbonate (27 mg, 0.2 mmol, 2 equiv.) and phenylboronic acid (13.5 mg, 0.11 mmol, 1.1 equiv.) was evacuated and backfilled with argon three times, then DMSO (1 ml) was added under positive pressure of argon. The resulting dark green mixture was stirred at ambient temperature for 15 minutes. Upon completeness the color changes to light-yellow. Hexafluorobenzene (11.5 µl, 0.1 mmol) was added as a standard and an aliquot of the mixture was analyzed directly by ^{19}F NMR in $CDCl_3$, which indicated clean formation of trifluoromethylbenzene **9** in quantitative yield.

Experiments 2-3. Catalytic trifluoromethylation with CuCl/KF/TMSCF$_3$ system. To the 15 ml Schlenk tube charged with phenylboronic acid (24 mg, 0.2 mmol, 1.0 equiv.), CuCl (2 mg, 0.02 mmol, 10 mol.%), and KF (35 mg, 0.6 mmol, 3.0 equiv.) was evacuated and backfilled with argon three times, then DMF (1 ml) was added under positive pressure of argon, followed by $TMSCF_3$ (0.4 mmol, 2.0 equiv.). The resulting mixture was stirred at ambient temperature under argon (for experiment 3), or exposed to air (experiment 2). The mixtures were stirred at ambient conditions for 4 hours. Hexafluorobenzene (11.5 µl, 0.1 mmol) was added as a standard and an aliquot of the mixture was analyzed directly by ^{19}F NMR in CD_3CN, which indicated formation of trifluoromethylbenzene **9** in 52% yield (for experiment 2) or in trace amounts (for experiment 3).

Experiment 4. Trifluoromethylation with 3/KF/TMSCF$_3$ system. A 15 ml Schlenk tube charged with phenylboronic acid (24 mg, 0.2 mmol, 1.0 equiv.), heterocubane **3** (9 mg, 0.01 mmol, 5 mol.%), and KF (35 mg, 0.6 mmol, 3.0 equiv.) was evacuated and backfilled with argon three times, then DMF (1 ml) was added under positive pressure of argon, followed by TMSCF$_3$ (0.4 mmol, 2.0 equiv.). The resulting mixture was exposed to air and stirred at ambient conditions for 4 hours. Hexafluorobenzene (11.5 µl, 0.1 mmol) was added as a standard and an aliquot of the mixture was analyzed directly by ^{19}F NMR in CD$_3$CN, which indicated formation of trifluoromethylbenzene **9** in 19% yield.

Trifluoromethylation of trimethoxybenzene

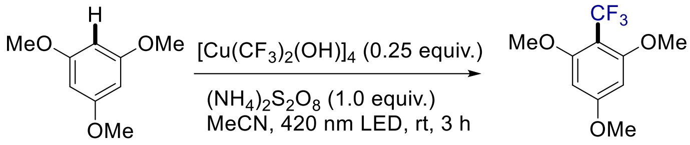

10, 63%

A 15 ml Schlenk tube charged with trimethoxybenzene (42 mg, 0.25 mmol) and ammonium persulfate (57 mg, 0.25 mmol) was evacuated and backfilled with argon three times, then a solution of **3** (54.5 mg, 0.25 mmol based on monomer) in MeCN (1 ml) was added under positive pressure of argon. Mixture was stirred at ambient temperature under 420 nm LED irradiation (2*30 W LED lamps) for 3 hours. Then it was quenched with ethyl acetate (25 ml) and washed with water (5 ml). Organic layer was separated, dried over anhydrous magnesium sulfate and evaporated under reduced pressure. Product was purified by column chromatography on silica gel (hexane/EtOAc, 5:1 to 1:1) to give product **10** (37 mg, 63%) as a white solid.

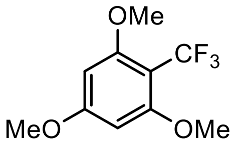

^{1}H NMR (400 MHz, MeCN-*d*$_3$) δ 6.13 (s, 2H, Ar), 3.84 (s, 9H, OMe); **^{13}C NMR** (101 MHz, MeCN-*d*$_3$) δ 163.6, 160.5, 124.4 (q, J = 273.3 Hz), 100.3 (q, J = 30.0 Hz), 91.3, 56.4 (OMe), 55.3 (OMe) ppm; **^{19}F NMR** (376 MHz, MeCN-*d*$_3$) δ −54.1 (s, 3F). NMR matches previously reported data.[2]

X-ray crystallography

General

Data for complexes **3-5, 8** were collected from a shock-cooled single crystal at 150.00 K on a Bruker D8 VENTURE dual wavelength Mo/Cu three-circle diffractometer with a microfocus sealed X-ray tube using a mirror optics as monochromator and a Bruker PHOTON II detector. The diffractometer was equipped with an Oxford Cryostream 700 low temperature device and used MoK_{α} radiation (λ = 0.71073 Å). All data were integrated with SAINT V8.41 and a multi-scan absorption correction using SADABS 2016/2 was applied.[3,4] The structure was solved by direct methods with SHELXT 2018/2 and refined by full-matrix least-squares methods against F^2 using XL.[5,6] All non-hydrogen atoms were refined with anisotropic displacement parameters. The heteroatom-bound hydrogen atoms were refined isotropic on calculated positions using a riding model with their U_{iso} values constrained to 1.5 times the U_{eq} of their pivot atoms for terminal sp^3 carbon atoms and 1.2 times for all other carbon atoms. Crystallographic data for the structures reported in this paper have been deposited with the Cambridge Crystallographic Data Centre.[7] CCDC 2481836, 2481839-2481841 contain the supplementary crystallographic data for this paper. These data can be obtained free of charge from The Cambridge Crystallographic Data Centre via www.ccdc.cam.ac.uk/structures. This reports and the CIF files were generated using FinalCif.[8]

Table 1. Crystal data and structure refinement for Cu(III) trifluoromethyl hydroxide tetramer 3

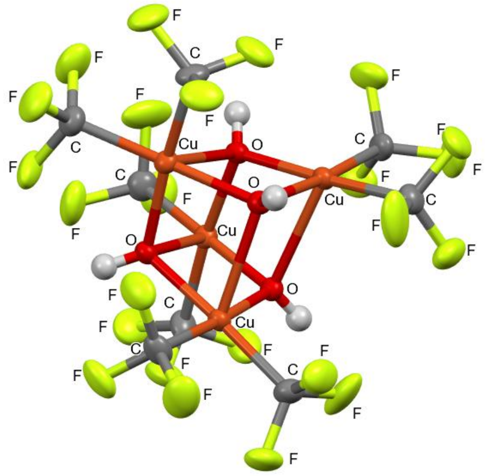

CCDC number	2481836
Empirical formula	$C_8H_4Cu_4F_{24}O_4$
Formula weight	874.27
Temperature [K]	150.00
Crystal system	tetragonal
Space group (number)	$I\bar{4}$ (82)
a [Å]	23.0647(7)
b [Å]	23.0647(7)
c [Å]	10.5677(4)
α [°]	90
β [°]	90
γ [°]	90
Volume [Å^3]	5621.8(4)
Z	10
ρ_{calc} [gcm^{-3}]	2.582
μ [mm^{-1}]	3.945
F(000)	4160
Crystal size [mm^3]	0.166×0.237×0.532
Crystal colour	colourless
Crystal shape	plate
Radiation	MoK_α (λ=0.71073 Å)
2θ range [°]	4.24 to 50.73 (0.83 Å)
Index ranges	$-27 \le h \le 27$<br>$-27 \le k \le 27$<br>$-12 \le l \le 12$
Reflections collected	35342
Independent reflections	5170<br>R_{int} = 0.0502<br>R_{sigma} = 0.0322
Completeness to θ = 25.242°	99.9 %
Data / Restraints / Parameters	5170 / 36 / 452
Absorption correction T_{min}/T_{max} (method)	0.4514 / 0.7452 (multi-scan)
Goodness-of-fit on F^2	1.150
Final R indexes [$I \geq 2\sigma(I)$]	R_1 = 0.0285<br>wR_2 = 0.0632
Final R indexes [all data]	R_1 = 0.0341<br>wR_2 = 0.0689
Largest peak/hole [eÅ^{-3}]	0.59/−0.63
Flack X parameter	0.041(19)

Refinement details for 3

Refined as a 2-component inversion twin.

Table 2. Atomic coordinates and U_{eq} [Å^2] for 3

Atom	*x*	*y*	*z*	U_{eq}
Cu4	0.27361(4)	0.64549(3)	0.89242(8)	0.02003(18)
Cu5	0.24650(3)	0.58342(4)	0.61558(8)	0.01901(18)
Cu1	0.07075(3)	0.51470(3)	1.15504(8)	0.02033(18)
Cu3	0.31053(4)	0.51857(3)	0.82836(8)	0.02156(19)
Cu2	0.36733(4)	0.63343(4)	0.68255(8)	0.02205(19)
O4	0.2407(2)	0.5589(2)	0.7880(4)	0.0209(10)
H4	0.209997	0.544628	0.811498	0.025
O1	0.00147(19)	0.55955(19)	1.1682(5)	0.0205(10)
H1	−0.001920	0.587634	1.121224	0.025
F29	0.1435(2)	0.5682(2)	0.4835(5)	0.0438(12)
O5	0.2917(2)	0.6632(2)	0.7208(4)	0.0205(10)
H5	0.283885	0.695913	0.695437	0.025
O2	0.32745(19)	0.56512(19)	0.6296(4)	0.0196(10)
H2	0.342119	0.545768	0.572935	0.024
O3	0.33808(19)	0.59246(19)	0.8828(5)	0.0218(10)
H3	0.361713	0.593080	0.940807	0.026
F2	0.1362(2)	0.5591(2)	0.9583(5)	0.0463(13)
F5	0.17712(19)	0.4997(2)	1.2375(5)	0.0441(12)
F27	0.2162(2)	0.6465(2)	0.3984(5)	0.0516(14)
F24	0.2237(3)	0.7395(2)	1.0136(5)	0.0520(14)
F26	0.3061(2)	0.6383(2)	0.4352(4)	0.0380(11)
F1	0.0828(2)	0.4867(2)	0.9048(5)	0.0497(13)
F20	0.2072(2)	0.6369(2)	1.1176(5)	0.0460(12)
F21	0.2985(2)	0.6500(3)	1.1376(4)	0.0529(15)
F22	0.1628(2)	0.6859(2)	0.9119(6)	0.0523(14)
F23	0.2203(3)	0.7428(2)	0.8121(5)	0.0518(14)
F4	0.1725(2)	0.4616(3)	1.0531(5)	0.0531(14)
F25	0.2582(3)	0.5645(2)	0.3674(4)	0.0505(14)
F6	0.1310(2)	0.4197(2)	1.2078(5)	0.0445(13)
F14	0.3692(3)	0.4268(2)	0.9406(7)	0.0693(19)
F9	0.4732(2)	0.6480(2)	0.5611(6)	0.0578(16)
F11	0.4586(2)	0.6990(2)	0.7848(6)	0.0565(15)
F7	0.4348(2)	0.5654(3)	0.5387(7)	0.076(2)
F3	0.0451(3)	0.5695(3)	0.9385(5)	0.0618(17)
F19	0.2641(3)	0.5677(2)	1.0759(5)	0.0522(15)
F18	0.3188(3)	0.4092(3)	0.7149(7)	0.0733(19)
F30	0.1459(2)	0.6429(3)	0.6030(7)	0.0696(19)
F12	0.3752(2)	0.7230(3)	0.8404(7)	0.0695(19)
F10	0.4042(3)	0.7422(2)	0.6521(7)	0.081(2)
F28	0.1373(2)	0.5576(3)	0.6810(6)	0.074(2)
F8	0.4749(3)	0.5882(3)	0.7129(7)	0.0736(19)
F15	0.4046(3)	0.5078(3)	0.9751(8)	0.093(3)
C5	0.3777(3)	0.4768(3)	0.8872(9)	0.038(2)
F16	0.2480(3)	0.4567(3)	0.6647(8)	0.084(2)
F13	0.4151(3)	0.4691(4)	0.7975(8)	0.103(3)

F17	0.2527(4)	0.4189(3)	0.8502(8)	0.111(3)
C3	0.4422(3)	0.6081(3)	0.6220(9)	0.0353(19)
C4	0.4054(4)	0.7029(4)	0.7427(9)	0.037(2)
C7	0.2578(3)	0.6250(3)	1.0662(7)	0.0291(17)
C6	0.2814(4)	0.4451(3)	0.7649(8)	0.0357(19)
C1	0.0853(3)	0.5326(3)	0.9806(7)	0.0321(17)
C9	0.2545(3)	0.6102(3)	0.4429(7)	0.0290(17)
C8	0.2165(4)	0.7069(4)	0.9103(8)	0.0381(19)
C2	0.1421(3)	0.4710(3)	1.1591(8)	0.0302(16)
C10	0.1634(3)	0.5891(4)	0.5924(8)	0.0375(19)

Table 3. Bond lengths and angles for 3

**Atom–Atom**	**Length [Å]**
Cu4–O4	2.405(5)
Cu4–O5	1.906(5)
Cu4–O3	1.928(5)
Cu4–C7	1.931(8)
Cu4–C8	1.943(8)
Cu5–O4	1.913(5)
Cu5–O5	2.390(5)
Cu5–O2	1.920(4)
Cu5–C9	1.935(8)
Cu5–C10	1.936(8)
Cu1–O1$^{\#1}$	1.922(5)
Cu1–O1$^{\#2}$	2.393(5)
Cu1–O1	1.909(4)
Cu1–C1	1.919(8)
Cu1–C2	1.929(7)
Cu3–O4	1.907(5)
Cu3–O2	2.391(5)
Cu3–O3	1.908(5)
Cu3–C5	1.927(8)
Cu3–C6	1.942(8)
Cu2–O5	1.918(5)
Cu2–O2	1.908(5)
Cu2–O3	2.414(5)
Cu2–C3	1.932(8)
Cu2–C4	1.933(8)
O4–H4	0.8200
O1–H1	0.8200
F29–C10	1.330(9)
O5–H5	0.8200
O2–H2	0.8200
O3–H3	0.8200
F2–C1	1.344(9)
F5–C2	1.333(9)
F27–C9	1.306(9)
F24–C8	1.336(10)
F26–C9	1.358(9)
F1–C1	1.330(9)
F20–C7	1.316(9)
F21–C7	1.336(9)
F22–C8	1.329(10)
F23–C8	1.331(10)
F4–C2	1.340(9)
F25–C9	1.324(9)
F6–C2	1.315(9)
F14–C5	1.299(9)
F9–C3	1.331(9)
F11–C4	1.310(10)
F7–C3	1.332(10)
F3–C1	1.335(10)
F19–C7	1.333(9)
F18–C6	1.307(10)
F30–C10	1.309(10)
F12–C4	1.330(10)
F10–C4	1.320(10)
F28–C10	1.330(10)
F8–C3	1.305(11)
F15–C5	1.326(11)
C5–F13	1.295(12)
F16–C6	1.337(10)
F17–C6	1.271(11)
**Atom–Atom–Atom**	**Angle [°]**
O5–Cu4–O4	79.09(18)
O5–Cu4–O3	85.3(2)
O5–Cu4–C7	177.6(3)
O5–Cu4–C8	94.9(3)
O3–Cu4–O4	72.07(18)
O3–Cu4–C7	92.3(3)
O3–Cu4–C8	172.0(3)
C7–Cu4–O4	100.0(3)
C7–Cu4–C8	87.6(4)
C8–Cu4–O4	115.9(3)
O4–Cu5–O5	79.34(18)
O4–Cu5–O2	85.9(2)
O4–Cu5–C9	177.9(3)
O4–Cu5–C10	94.1(3)
O2–Cu5–O5	73.10(18)

O2–Cu5–C9	92.9(3)
O2–Cu5–C10	170.8(3)
C9–Cu5–O5	98.7(2)
C9–Cu5–C10	87.4(3)
C10–Cu5–O5	116.0(3)
O1–Cu1–O1$^{\#1}$	85.5(2)
O1$^{\#1}$–Cu1–O1$^{\#2}$	73.2(2)
O1–Cu1–O1$^{\#2}$	78.51(19)
O1–Cu1–C1	95.7(3)
O1$^{\#1}$–Cu1–C2	89.5(3)
O1–Cu1–C2	174.3(3)
C1–Cu1–O1$^{\#1}$	177.3(3)
C1–Cu1–O1$^{\#2}$	109.4(3)
C1–Cu1–C2	89.1(4)
C2–Cu1–O1$^{\#2}$	102.6(3)
O4–Cu3–O2	73.89(17)
O4–Cu3–O3	85.02(19)
O4–Cu3–C5	173.8(3)
O4–Cu3–C6	93.2(3)
O3–Cu3–O2	79.03(18)
O3–Cu3–C5	94.6(3)
O3–Cu3–C6	177.1(3)
C5–Cu3–O2	112.1(3)
C5–Cu3–C6	87.3(4)
C6–Cu3–O2	98.3(3)
O5–Cu2–O3	72.63(18)
O5–Cu2–C3	172.3(3)
O5–Cu2–C4	92.7(3)
O2–Cu2–O5	85.36(19)
O2–Cu2–O3	78.42(17)
O2–Cu2–C3	94.8(3)
O2–Cu2–C4	177.4(3)
C3–Cu2–O3	115.0(3)
C3–Cu2–C4	87.3(3)
C4–Cu2–O3	99.4(3)
Cu4–O4–H4	117.8
Cu5–O4–Cu4	99.75(19)
Cu5–O4–H4	117.8
Cu3–O4–Cu4	92.08(18)
Cu3–O4–Cu5	107.4(2)
Cu3–O4–H4	117.8
Cu1–O1–Cu1$^{\#3}$	107.8(2)
Cu1–O1–Cu1$^{\#2}$	101.00(19)
Cu1$^{\#3}$–O1–Cu1$^{\#2}$	91.02(18)
Cu1$^{\#3}$–O1–H1	117.6
Cu1$^{\#2}$–O1–H1	117.6
Cu1–O1–H1	117.6
Cu4–O5–Cu5	100.48(19)
Cu4–O5–Cu2	108.8(2)
Cu4–O5–H5	117.4
Cu5–O5–H5	117.4
Cu2–O5–Cu5	91.30(17)
Cu2–O5–H5	117.4

Cu5–O2–Cu3	90.45(17)
Cu5–O2–H2	117.7
Cu3–O2–H2	117.7
Cu2–O2–Cu5	108.1(2)
Cu2–O2–Cu3	101.06(19)
Cu2–O2–H2	117.7
Cu4–O3–Cu2	90.76(18)
Cu4–O3–H3	117.5
Cu3–O3–Cu4	109.0(2)
Cu3–O3–Cu2	100.3(2)
Cu3–O3–H3	117.5
Cu2–O3–H3	117.5
F14–C5–Cu3	117.6(6)
F14–C5–F15	104.2(8)
F15–C5–Cu3	109.5(6)
F13–C5–Cu3	111.6(7)
F13–C5–F14	107.2(8)
F13–C5–F15	105.9(8)
F9–C3–Cu2	115.5(6)
F9–C3–F7	105.1(8)
F7–C3–Cu2	109.1(5)
F8–C3–Cu2	112.3(6)
F8–C3–F9	106.8(7)
F8–C3–F7	107.5(8)
F11–C4–Cu2	118.8(6)
F11–C4–F12	104.5(7)
F11–C4–F10	108.2(7)
F12–C4–Cu2	107.9(5)
F10–C4–Cu2	108.8(6)
F10–C4–F12	108.2(8)
F20–C7–Cu4	120.6(6)
F20–C7–F21	107.6(6)
F20–C7–F19	105.7(7)
F21–C7–Cu4	107.4(5)
F19–C7–Cu4	107.2(5)
F19–C7–F21	107.9(7)
F18–C6–Cu3	117.7(6)
F18–C6–F16	100.8(7)
F16–C6–Cu3	107.4(5)
F17–C6–Cu3	110.5(6)
F17–C6–F18	109.2(8)
F17–C6–F16	110.8(8)
F2–C1–Cu1	114.8(5)
F1–C1–Cu1	113.6(5)
F1–C1–F2	107.1(6)
F1–C1–F3	106.1(7)
F3–C1–Cu1	109.6(5)
F3–C1–F2	105.0(7)
F27–C9–Cu5	118.7(6)
F27–C9–F26	105.4(6)
F27–C9–F25	109.6(6)
F26–C9–Cu5	107.1(5)
F25–C9–Cu5	108.7(5)

F25–C9–F26	106.8(6)
F24–C8–Cu4	114.0(6)
F22–C8–Cu4	111.5(6)
F22–C8–F24	108.1(7)
F22–C8–F23	107.3(7)
F23–C8–Cu4	109.5(6)
F23–C8–F24	106.2(6)
F5–C2–Cu1	105.8(5)
F5–C2–F4	106.4(6)
F4–C2–Cu1	120.9(6)
F6–C2–Cu1	108.3(5)
F6–C2–F5	108.7(7)
F6–C2–F4	106.4(6)
F29–C10–Cu5	115.3(6)
F29–C10–F28	104.8(7)
F30–C10–Cu5	111.1(6)
F30–C10–F29	108.0(7)
F30–C10–F28	108.6(8)
F28–C10–Cu5	108.8(6)

Symmetry transformations used to generate equivalent atoms:
#1: -0.5+Y, 0.5-X, 2.5-Z; #2: -X, 1-Y, +Z; #3: 0.5-Y, 0.5+X, 2.5-Z;

Bis(trifluoromethyl)copper(III) pyridine-2-carboxylate 4

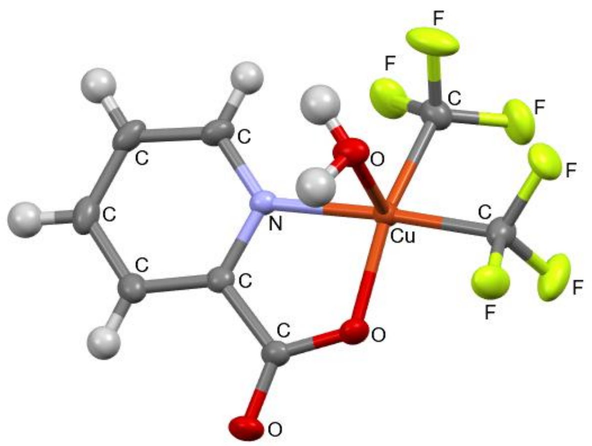

Table 4. Crystal data and structure refinement for 4

CCDC number	2481839
Empirical formula	$C_{10}H_9CuF_6N_2O_3$
Formula weight	382.73
Temperature [K]	150.00
Crystal system	triclinic
Space group (number)	$P\bar{1}$ (2)
a [Å]	7.0179(3)
b [Å]	8.0222(4)
c [Å]	12.2963(6)
α [°]	86.173(2)
β [°]	84.951(2)
γ [°]	86.220(2)
Volume [Å^3]	686.79(6)
Z	2
ρ_{calc} [gcm^{-3}]	1.851
μ [mm^{-1}]	1.673
F(000)	380
Crystal size [mm^3]	0.127×0.328×0.409
Crystal colour	clear yellow
Crystal shape	block
Radiation	MoK_α (λ=0.71073 Å)
2θ range [°]	5.10 to 50.74 (0.83 Å)
Index ranges	$-8 \le h \le 8$<br>$-9 \le k \le 9$<br>$-14 \le l \le 14$
Reflections collected	30680
Independent reflections	2529<br>R_{int} = 0.0568<br>R_{sigma} = 0.0249
Completeness to θ = 25.242°	100.0 %
Data / Restraints / Parameters	2529 / 0 / 201
Absorption correction T_{min}/T_{max} (method)	0.6025 / 0.7452 (multi-scan)
Goodness-of-fit on F^2	1.085
Final R indexes [$I \geq 2\sigma(I)$]	R_1 = 0.0221<br>wR_2 = 0.0535
Final R indexes [all data]	R_1 = 0.0256<br>wR_2 = 0.0552
Largest peak/hole [eÅ^{-3}]	0.26/−0.34

Table 5. Atomic coordinates and U_{eq} [Å^2] for 4

Atom	*x*	*y*	*z*	U_{eq}
Cu1	0.63483(3)	0.89778(3)	0.69802(2)	0.01843(8)
F2	0.39094(18)	0.66397(16)	0.77303(10)	0.0354(3)
F4	0.63462(18)	1.05343(16)	0.89543(9)	0.0335(3)
F6	0.75830(19)	1.18415(15)	0.75253(10)	0.0348(3)
F3	0.34466(18)	0.87335(17)	0.87046(10)	0.0387(3)
F1	0.59261(18)	0.70494(19)	0.88871(11)	0.0428(4)
F5	0.4525(2)	1.18059(17)	0.78045(12)	0.0443(4)
O1	0.7036(2)	1.04016(16)	0.57271(11)	0.0236(3)
O2	0.7613(2)	1.03984(18)	0.39159(11)	0.0287(3)
O00A	0.94009(19)	0.84687(18)	0.75538(11)	0.0266(3)
H00A	1.024845	0.870601	0.701236	0.040
H00B	0.961261	0.739752	0.770447	0.040
N1	0.6825(2)	0.71671(19)	0.59667(12)	0.0175(3)
C4	0.7346(3)	0.7794(2)	0.49440(15)	0.0181(4)
N2	1.0054(3)	0.5015(2)	0.84135(16)	0.0366(4)
C3	0.7341(3)	0.9672(2)	0.48215(15)	0.0191(4)
C5	0.7874(3)	0.6790(3)	0.40937(16)	0.0231(4)
H5	0.823129	0.726566	0.338269	0.028
C8	0.6875(3)	0.5501(2)	0.61536(16)	0.0227(4)
H8	0.654672	0.504859	0.687455	0.027
C7	0.7385(3)	0.4418(2)	0.53370(17)	0.0254(4)
H7	0.739627	0.324122	0.549268	0.031
C1	0.4832(3)	0.7825(3)	0.81505(16)	0.0251(4)
C9	1.0276(3)	0.3750(3)	0.88740(17)	0.0269(4)
C2	0.6158(3)	1.0868(3)	0.78892(16)	0.0264(4)
C6	0.7878(3)	0.5069(3)	0.42916(17)	0.0266(4)
H6	0.821516	0.434681	0.371432	0.032
C10	1.0547(3)	0.2127(3)	0.94593(18)	0.0338(5)
H10A	1.050433	0.123911	0.895337	0.051
H10B	0.952629	0.200364	1.005107	0.051
H10C	1.179317	0.204434	0.976779	0.051

U_{eq} is defined as 1/3 of the trace of the orthogonalized U_{ij} tensor.

Table 6. Bond lengths and angles for 4

Atom–Atom	Length [Å]
Cu1–O1	1.9023(13)
Cu1–O00A	2.3164(13)
Cu1–N1	1.9696(15)
Cu1–C1	1.934(2)
Cu1–C2	1.934(2)
F2–C1	1.341(2)
F4–C2	1.335(2)
F6–C2	1.337(2)
F3–C1	1.340(2)
F1–C1	1.335(2)
F5–C2	1.337(2)
O1–C3	1.287(2)
O2–C3	1.227(2)
O00A–H00A	0.8727
O00A–H00B	0.8727
N1–C4	1.352(2)
N1–C8	1.340(2)
C4–C3	1.504(3)
C4–C5	1.373(3)
N2–C9	1.137(3)
C5–H5	0.9500
C5–C6	1.386(3)
C8–H8	0.9500
C8–C7	1.380(3)
C7–H7	0.9500

C7–C6	1.379(3)
C9–C10	1.455(3)
C6–H6	0.9500
C10–H10A	0.9800
C10–H10B	0.9800
C10–H10C	0.9800
**Atom–Atom–Atom**	**Angle [°]**
O1–Cu1–O00A	96.99(5)
O1–Cu1–N1	84.52(6)
O1–Cu1–C1	161.37(7)
O1–Cu1–C2	90.34(7)
N1–Cu1–O00A	90.40(6)
C1–Cu1–O00A	101.14(7)
C1–Cu1–N1	99.50(8)
C2–Cu1–O00A	85.59(7)
C2–Cu1–N1	173.07(8)
C2–Cu1–C1	86.85(9)
C3–O1–Cu1	115.33(12)
Cu1–O00A–H00A	109.5
Cu1–O00A–H00B	109.5
H00A–O00A–H00B	104.4
C4–N1–Cu1	110.62(12)
C8–N1–Cu1	130.85(13)
C8–N1–C4	118.22(16)
N1–C4–C3	114.60(16)
N1–C4–C5	122.45(18)
C5–C4–C3	122.94(17)
O1–C3–C4	114.47(16)
O2–C3–O1	124.79(18)
O2–C3–C4	120.74(17)
C4–C5–H5	120.6
C4–C5–C6	118.79(18)
C6–C5–H5	120.6
N1–C8–H8	118.8
N1–C8–C7	122.38(18)
C7–C8–H8	118.8
C8–C7–H7	120.5
C6–C7–C8	118.99(19)
C6–C7–H7	120.5
F2–C1–Cu1	108.85(13)
F3–C1–Cu1	117.34(14)
F3–C1–F2	104.44(16)
F1–C1–Cu1	111.74(13)
F1–C1–F2	106.80(17)
F1–C1–F3	106.96(16)
N2–C9–C10	179.5(2)
F4–C2–Cu1	116.85(14)
F4–C2–F6	105.84(16)
F4–C2–F5	107.20(17)
F6–C2–Cu1	107.10(13)
F5–C2–Cu1	112.52(13)
F5–C2–F6	106.68(17)
C5–C6–H6	120.4
C7–C6–C5	119.14(19)
C7–C6–H6	120.4
C9–C10–H10A	109.5
C9–C10–H10B	109.5
C9–C10–H10C	109.5
H10A–C10–H10B	109.5
H10A–C10–H10C	109.5
H10B–C10–H10C	109.5

Table 7. Torsion angles for 4

Atom–Atom–Atom–Atom	Torsion Angle [°]
Cu1–O1–C3–O2	172.48(15)
Cu1–O1–C3–C4	−7.50(19)
Cu1–N1–C4–C3	−2.79(18)
Cu1–N1–C4–C5	175.93(14)
Cu1–N1–C8–C7	−174.78(14)
N1–C4–C3–O1	6.9(2)
N1–C4–C3–O2	−173.13(17)
N1–C4–C5–C6	−0.2(3)
N1–C8–C7–C6	0.6(3)
C4–N1–C8–C7	−1.8(3)
C4–C5–C6–C7	−1.1(3)
C3–C4–C5–C6	178.46(17)
C5–C4–C3–O1	−171.86(17)
C5–C4–C3–O2	8.2(3)
C8–N1–C4–C3	−177.10(15)
C8–N1–C4–C5	1.6(3)

C8–C7–C6–C5	0.9(3)

(Acetato)(bis(trifluoromethyl)copper(III) 5

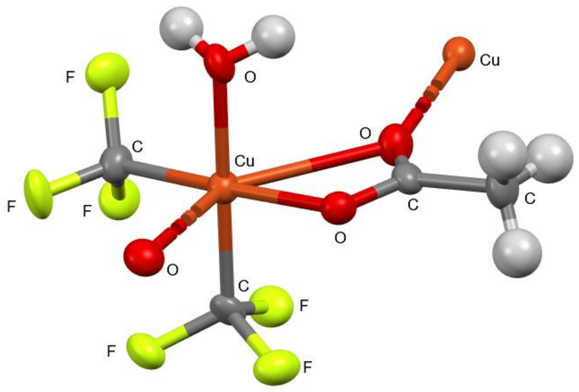

Table 7. Crystal data and structure refinement for 5

CCDC number	2481840
Empirical formula	$C_4H_7CuF_6O_4$
Formula weight	296.64
Temperature [K]	150.00
Crystal system	monoclinic
Space group (number)	$P2_1/c$ (14)
a [Å]	7.5786(15)
b [Å]	12.770(3)
c [Å]	9.1246(17)
α [°]	90
β [°]	91.771(7)
γ [°]	90
Volume [Å^3]	882.7(3)
Z	4
ρ_{calc} [gcm^{-3}]	2.232
μ [mm^{-1}]	2.569
F(000)	584
Crystal size [mm^3]	0.067×0.089×0.105
Crystal colour	clear yellow
Crystal shape	plate
Radiation	MoK_α (λ=0.71073 Å)
2θ range [°]	4.47 to 50.82 (0.83 Å)
Index ranges	$-9 \le h \le 9$<br>$-15 \le k \le 15$<br>$-9 \le l \le 11$
Reflections collected	8926
Independent reflections	1602<br>R_{int} = 0.0915<br>R_{sigma} = 0.0664
Completeness to θ = 25.242°	99.2 %
Data / Restraints / Parameters	1602 / 3 / 147
Absorption correction T_{min}/T_{max} (method)	0.4917 / 0.7452 (multi-scan)
Goodness-of-fit on F^2	1.171
Final R indexes [$I \geq 2\sigma(I)$]	R_1 = 0.0792<br>wR_2 = 0.1572
Final R indexes [all data]	R_1 = 0.1147<br>wR_2 = 0.1808
Largest peak/hole [eÅ^{-3}]	0.98/−0.93

Refinement details for 5

Refined as a 2-component twin.

Table 8. Atomic coordinates and U_{eq} [Å^2] for 5

Atom	*x*	*y*	*z*	U_{eq}
Cu1	0.70476(16)	0.28851(10)	0.28038(16)	0.0237(4)
F5	0.7302(8)	0.0942(5)	0.1661(8)	0.0378(17)
F3	0.9151(8)	0.4506(5)	0.3572(8)	0.0387(18)
F4	0.9259(9)	0.1264(5)	0.3362(8)	0.0383(17)
F6	0.9561(8)	0.1853(5)	0.1175(7)	0.0339(17)
F2	0.9530(8)	0.4008(6)	0.1375(7)	0.0377(18)
F1	1.0761(8)	0.3184(5)	0.3199(8)	0.0348(17)
O4	0.6551(10)	0.6089(6)	0.4305(10)	0.0282(19)
O1	0.4988(9)	0.2039(6)	0.3116(8)	0.0249(17)
O3	0.5711(9)	0.4097(6)	0.3418(9)	0.0282(18)
H3A	0.517043	0.395035	0.422046	0.042
H3B	0.643372	0.460150	0.366507	0.042
O2	0.6210(9)	0.1966(6)	0.5360(9)	0.0313(19)
C2	0.8405(13)	0.1678(9)	0.2219(12)	0.025(2)
C1	0.9279(14)	0.3673(9)	0.2726(14)	0.029(3)
C3	0.4973(14)	0.1735(9)	0.4468(13)	0.025(3)
C4	0.3420(15)	0.1093(10)	0.4897(15)	0.036(3)
H4A	0.232950	0.147859	0.466271	0.054
H4B	0.350031	0.095132	0.595243	0.054
H4C	0.341405	0.042988	0.435699	0.054
H4D	0.764(4)	0.616(11)	0.445(14)	0.05(4)
H4E	0.611(17)	0.62(2)	0.346(9)	0.19(13)

Table 9. Bond lengths and angles for 5

Atom–Atom	Length [Å]
Cu1–O1	1.926(7)
Cu1–O3	1.942(8)
Cu1–O2#1	2.307(8)
Cu1–C2	1.937(11)
Cu1–C1	1.971(11)
F5–C2	1.348(12)
F3–C1	1.320(13)
F4–C2	1.321(13)
F6–C2	1.333(13)
F2–C1	1.324(14)
F1–C1	1.344(12)
O4–H4D	0.84(2)
O4–H4E	0.84(2)
O1–C3	1.294(13)
O3–H3A	0.8707
O3–H3B	0.8708
O2–C3	1.258(13)
C3–C4	1.496(15)
C4–H4A	0.9800
C4–H4B	0.9800
C4–H4C	0.9800

Atom–Atom–Atom	Angle [°]
O1–Cu1–O3	88.4(3)
O1–Cu1–O2#1	89.4(3)
O1–Cu1–C2	92.0(4)
O1–Cu1–C1	172.5(4)
O3–Cu1–O2#1	94.7(3)
O3–Cu1–C1	93.4(4)
C2–Cu1–O3	179.1(4)
C2–Cu1–O2#1	86.1(4)
C2–Cu1–C1	86.1(5)
C1–Cu1–O2#1	97.7(4)
H4D–O4–H4E	119(4)
C3–O1–Cu1	109.9(7)
Cu1–O3–H3A	109.5
Cu1–O3–H3B	109.6
H3A–O3–H3B	104.5
C3–O2–Cu1#2	145.3(7)
F5–C2–Cu1	109.3(7)
F4–C2–Cu1	110.6(7)

F4–C2–F5	107.6(9)
F4–C2–F6	108.4(8)
F6–C2–Cu1	115.4(8)
F6–C2–F5	105.1(9)
F3–C1–Cu1	108.1(7)
F3–C1–F2	107.4(9)
F3–C1–F1	105.3(9)
F2–C1–Cu1	110.3(8)
F2–C1–F1	107.8(9)
F1–C1–Cu1	117.4(8)
O1–C3–C4	116.4(10)

O2–C3–O1	121.2(10)
O2–C3–C4	122.4(11)
C3–C4–H4A	109.5
C3–C4–H4B	109.5
C3–C4–H4C	109.5
H4A–C4–H4B	109.5
H4A–C4–H4C	109.5
H4B–C4–H4C	109.5

Symmetry transformations used to generate equivalent atoms:
#1: +X, 0.5-Y, -0.5+Z;   #2: +X, 0.5-Y, 0.5+Z;

(η^2-Thiobenzamido)bis(trifluoromethyl)-copper(III) 8

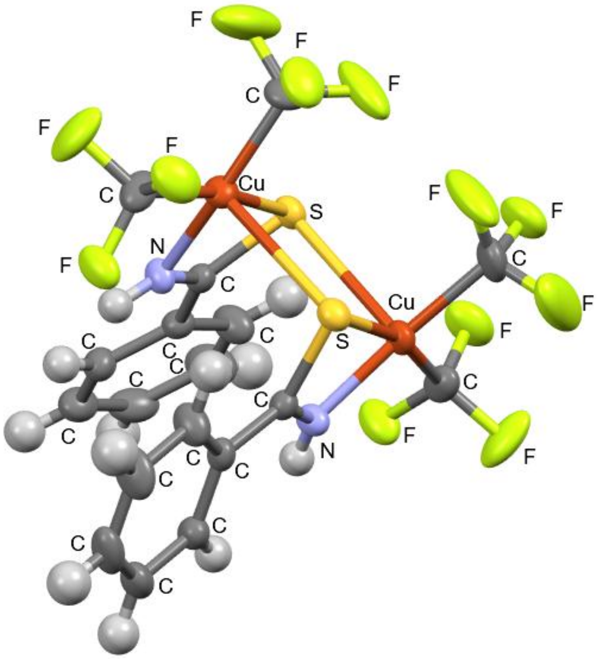

Table 10. Crystal data and structure refinement for 8

CCDC number	2481838
Empirical formula	$C_{18}H_{12}Cu_2F_{12}N_2S_2$
Formula weight	675.50
Temperature [K]	150.00
Crystal system	monoclinic
Space group (number)	$C2/c$ (15)
a [Å]	11.6062(4)
b [Å]	14.1352(5)
c [Å]	14.6945(6)
α [°]	90
β [°]	108.4570(10)
γ [°]	90
Volume [Å^3]	2286.72(15)
Z	4
ρ_{calc} [gcm^{-3}]	1.962
μ [mm^{-1}]	2.151
F(000)	1328
Crystal size [mm^3]	0.285×0.297×0.468
Crystal colour	red
Crystal shape	block
Radiation	MoK_α (λ=0.71073 Å)
2θ range [°]	4.87 to 50.73 (0.83 Å)
Index ranges	$-13 \le h \le 13$<br>$-16 \le k \le 16$<br>$-17 \le l \le 17$
Reflections collected	14794
Independent reflections	2086<br>R_{int} = 0.0326<br>R_{sigma} = 0.0226
Completeness to θ = 25.242°	99.9 %
Data / Restraints / Parameters	2086 / 152 / 230
Absorption correction T_{min}/T_{max} (method)	0.6107 / 0.7452 (multi-scan)
Goodness-of-fit on F^2	1.060
Final R indexes [$I \ge 2\sigma(I)$]	R_1 = 0.0217<br>wR_2 = 0.0552
Final R indexes [all data]	R_1 = 0.0236<br>wR_2 = 0.0563
Largest peak/hole [eÅ^{-3}]	0.19/−0.37

Table 11. Atomic coordinates and U_{eq} [Å^2] for 8

Atom	*x*	*y*	*z*	U_{eq}
Cu1	0.66681(2)	0.45160(2)	0.27398(2)	0.02923(9)
S1	0.56352(4)	0.45355(3)	0.38094(3)	0.02872(12)
N1	0.64901(14)	0.32392(10)	0.30775(10)	0.0276(3)
H1	0.670674	0.271675	0.284929	0.033
C4	0.57238(15)	0.26073(12)	0.43490(11)	0.0254(4)
C3	0.59700(15)	0.33369(12)	0.37272(12)	0.0240(3)
C9	0.61846(17)	0.16944(13)	0.43598(13)	0.0309(4)
H9	0.663651	0.153305	0.394432	0.037
C5	0.5064(2)	0.28314(14)	0.49607(14)	0.0399(5)
H5	0.475042	0.345212	0.495999	0.048
C8	0.59800(18)	0.10239(14)	0.49795(14)	0.0384(5)
H8	0.629860	0.040339	0.499072	0.046
C7	0.5323(2)	0.12483(15)	0.55748(14)	0.0439(5)
H7	0.518283	0.078103	0.599219	0.053
C6	0.4864(2)	0.21479(16)	0.55739(16)	0.0500(6)
H6	0.441119	0.230027	0.599124	0.060
C2A	0.6906(6)	0.5879(4)	0.2786(5)	0.0451(16)
F5A	0.6910(8)	0.6276(6)	0.1960(5)	0.0554(14)
F6A	0.7991(4)	0.6086(2)	0.3413(4)	0.099(2)
F4A	0.6076(5)	0.63638(19)	0.3020(4)	0.0830(13)
F6B	0.8326(6)	0.5943(5)	0.2863(7)	0.065(3)
F4B	0.6892(14)	0.6288(5)	0.3397(6)	0.070(3)
F5B	0.661(2)	0.6239(18)	0.1865(13)	0.059(4)
C2B	0.7147(12)	0.5820(10)	0.2706(12)	0.044(5)
C1B	0.7638(10)	0.4164(9)	0.1892(10)	0.0387(8)
F3B	0.7413(16)	0.4719(12)	0.1115(12)	0.053(4)
F1B	0.7362(9)	0.3291(4)	0.1506(6)	0.0456(18)
F2B	0.8845(5)	0.4179(11)	0.2250(4)	0.063(3)
C1A	0.7676(4)	0.4355(3)	0.1927(4)	0.0387(8)
F2A	0.8743(2)	0.4812(3)	0.22385(18)	0.0554(8)
F1A	0.7955(4)	0.3442(2)	0.1918(3)	0.0612(11)
F3A	0.7148(6)	0.4628(5)	0.1020(5)	0.0501(14)

Table 12. Bond lengths and angles for 8

Atom–Atom	Length [Å]
Cu1–S1	2.2586(5)
Cu1–N1	1.9003(14)
Cu1–C3	2.5069(17)
Cu1–C2A	1.944(5)
Cu1–C2B	1.930(14)
Cu1–C1B	1.989(13)
Cu1–C1A	1.932(5)
S1–C3	1.7509(17)
N1–H1	0.8800
N1–C3	1.288(2)
C4–C3	1.465(2)
C4–C9	1.395(3)
C4–C5	1.389(3)
C9–H9	0.9500
C9–C8	1.386(3)
C5–H5	0.9500
C5–C6	1.390(3)
C8–H8	0.9500
C8–C7	1.367(3)
C7–H7	0.9500
C7–C6	1.379(3)
C6–H6	0.9500
C2A–F5A	1.338(6)
C2A–F6A	1.336(6)
C2A–F4A	1.314(7)

F6B–C2B	1.326(12)
F4B–C2B	1.321(13)
F5B–C2B	1.334(13)
C1B–F3B	1.341(11)
C1B–F1B	1.353(11)
C1B–F2B	1.331(11)
C1A–F2A	1.342(5)
C1A–F1A	1.332(5)
C1A–F3A	1.336(5)
**Atom–Atom–Atom**	**Angle [°]**
S1–Cu1–C3	42.72(4)
N1–Cu1–S1	72.80(4)
N1–Cu1–C3	30.19(6)
N1–Cu1–C2A	163.3(2)
N1–Cu1–C2B	165.8(5)
N1–Cu1–C1B	93.2(4)
N1–Cu1–C1A	100.76(15)
C2A–Cu1–S1	93.7(2)
C2A–Cu1–C3	135.0(2)
C2A–Cu1–C1B	99.6(4)
C2B–Cu1–S1	103.1(4)
C2B–Cu1–C3	142.9(4)
C2B–Cu1–C1A	82.0(4)
C1B–Cu1–S1	165.8(4)
C1B–Cu1–C3	123.1(4)
C1A–Cu1–S1	172.07(16)
C1A–Cu1–C3	130.51(15)
C3–S1–Cu1	76.23(6)
Cu1–N1–H1	129.0
C3–N1–Cu1	101.94(11)
C3–N1–H1	129.0
C9–C4–C3	120.56(15)
C5–C4–C3	119.96(16)
C5–C4–C9	119.43(16)
S1–C3–Cu1	61.05(5)
N1–C3–Cu1	47.87(8)
N1–C3–S1	108.73(12)
N1–C3–C4	128.06(15)
C4–C3–Cu1	172.67(12)
C4–C3–S1	123.15(13)
C4–C9–H9	120.2
C8–C9–C4	119.68(18)
C8–C9–H9	120.2
C4–C5–H5	120.0
C4–C5–C6	120.04(19)
C6–C5–H5	120.0
C9–C8–H8	119.7
C7–C8–C9	120.53(19)
C7–C8–H8	119.7
C8–C7–H7	119.8
C8–C7–C6	120.48(18)
C6–C7–H7	119.8
C5–C6–H6	120.1
C7–C6–C5	119.8(2)
C7–C6–H6	120.1
F5A–C2A–Cu1	115.2(6)
F6A–C2A–Cu1	109.5(4)
F6A–C2A–F5A	105.1(6)
F4A–C2A–Cu1	114.7(4)
F4A–C2A–F5A	103.4(5)
F4A–C2A–F6A	108.3(5)
F6B–C2B–Cu1	114.3(9)
F6B–C2B–F5B	104.8(13)
F4B–C2B–Cu1	108.8(9)
F4B–C2B–F6B	105.7(11)
F4B–C2B–F5B	109.7(15)
F5B–C2B–Cu1	113.2(14)
F3B–C1B–Cu1	113.0(12)
F3B–C1B–F1B	102.7(12)
F1B–C1B–Cu1	112.7(8)
F2B–C1B–Cu1	118.8(9)
F2B–C1B–F3B	102.9(11)
F2B–C1B–F1B	105.1(9)
F2A–C1A–Cu1	114.0(3)
F1A–C1A–Cu1	108.4(3)
F1A–C1A–F2A	105.6(4)
F1A–C1A–F3A	107.9(5)
F3A–C1A–Cu1	114.0(4)
F3A–C1A–F2A	106.5(4)

Table 13. Torsion angles for 8

**Atom–Atom–Atom–Atom**	**Torsion Angle [°]**
Cu1–S1–C3–N1	−4.50(11)
Cu1–S1–C3–C4	172.99(15)
Cu1–N1–C3–S1	5.31(13)
Cu1–N1–C3–C4	−172.02(15)
C4–C9–C8–C7	0.5(3)
C4–C5–C6–C7	0.2(3)
C3–C4–C9–C8	177.58(16)
C3–C4–C5–C6	−177.90(19)
C9–C4–C3–S1	−169.51(13)
C9–C4–C3–N1	7.5(3)
C9–C4–C5–C6	−0.3(3)
C9–C8–C7–C6	−0.6(3)
C5–C4–C3–S1	8.1(2)
C5–C4–C3–N1	−174.93(18)
C5–C4–C9–C8	0.0(3)
C8–C7–C6–C5	0.3(3)

DFT calculations

The optimization of geometry of **3** was done at the B3LYP level with Zero-Order Regular Approximation relativistic Hamiltonian[9] using the ZORA-def2-TZVP basis set as implemented in the ORCA 6.1.0 software[10] for single state of the molecule with the initial coordinates taken from XRD. The computed geometry of **3** can be found below. The single point energy calculation and natural bonding orbital (NBO) analysis were done at the ωB97X-D3BJ def2-QZVPPD level of theory using the B3LYP/ZORA-def2-TZVP optimal geometry.

$[Cu(CF_3)_2(OH)]_4$ (**3**)

Cu	6.30362828727990	14.82788460753144	9.43014603267423
Cu	5.69564562809118	13.53306793686600	6.48178872533249
Cu	7.20827335289437	11.96899708185114	8.67956586853201
Cu	8.48694762987865	14.62251306951231	7.25459461220648
O	5.53341857691082	12.91326581227667	8.31279453399135
F	3.23626950752588	13.10013327331155	5.25790368795836
O	6.70085648186507	15.33924812858008	7.60002879802028
O	7.58080465263693	13.02820838566449	6.62058789728674
O	7.87976079842403	13.67632764873549	9.30900329630026
F	5.00859554173585	14.54356845685304	3.84126615400918
F	5.04548381602883	17.01236640439090	10.60292233547145
F	6.97827127037455	14.89683354673180	4.57743916547274
F	5.09131564478966	14.76780377596851	12.07667959021041
F	7.22533703593734	14.69110900856352	11.92966938475730
F	3.66548454194409	15.47037601732934	9.95874738282995

F	4.61157391593085	16.74474928595095	8.52817098113112
F	6.37866933831540	12.90309428057586	3.98066904451388
F	8.49168602223962	9.97524126393448	10.13953411765846
F	10.87797201608894	14.81226310687986	5.84145202938660
F	10.59038965940765	16.53312654707782	7.87791413596825
F	10.22161757333581	12.81421531636952	6.21315667351606
F	6.01745511793913	12.99037729388914	11.34582658236458
F	7.05850159032420	9.13970813717366	8.02871031825698
F	3.50869089394715	15.13939344661751	5.93794170521145
F	8.80140692606470	16.58636041243292	9.03707729238461
F	8.78746526669434	17.26025007105217	6.97894462614625
F	3.12181537806355	13.57197246823823	7.33817892493954
F	11.10403193053704	13.93010450807838	7.80820763484374
F	9.65669759709340	11.73757484051980	9.82874293604573
C	8.77893281446814	10.91324995128880	9.24318040354609
F	5.88059065069389	10.50127572197827	6.88806293605477
F	9.40688747757173	10.35983677070415	8.21326427655029
F	5.35486321063847	10.06465288031277	8.94288327866154
C	10.30358079145689	14.03597144626324	6.75412030608117
C	9.28424819814630	16.33918632609256	7.81187382972447
C	6.08733719158474	14.32805610225289	11.32701173982112
C	6.38029755085996	10.27186768723481	8.10996216880064

C	5.96504674296559	14.02018417747431	4.58891325298296
C	4.80627707440674	16.08867228063848	9.67821835367325
C	3.76969596759937	13.86567290917111	6.20427391118279
H	4.69285112351263	12.54075214629342	8.61262970712822
H	6.46809191048121	16.22671520208923	7.29440074866044
H	7.99205305926704	12.49833003216775	5.92394326249857
H	8.54518024404762	13.69044223308019	10.01059735721233

References

1. V. Motornov, B. Klepetářová, P. Beier, *Adv. Synth. Catal.* **2023**, *365*, 2858–2864.
2. H. R. Zhang, C. C. Feng, N. Chen, S. L. Zhang, *Angew. Chem. Int. Ed.* **2022**, *61*, e202209029.
3. Bruker, *SAINT*, *V8.41*, Bruker AXS Inc., Madison, Wisconsin, USA.
4. L. Krause, R. Herbst-Irmer, G. M. Sheldrick, D. Stalke, *J. Appl. Cryst.* **2015**, *48*, 3–10, doi:10.1107/S1600576714022985.
5. G. M. Sheldrick, *Acta Cryst.* **2015**, *A71*, 3–8, doi:10.1107/S2053273314026370.
6. G. M. Sheldrick, *Acta Cryst.* **2015**, *C71*, 3–8, doi:10.1107/S2053229614024218.
7. C. R. Groom, I. J. Bruno, M. P. Lightfoot, S. C. Ward, *Acta Cryst.* **2016**, *B72*, 171–179, doi:10.1107/S2052520616003954.
8. D. Kratzert, *FinalCif*, *V152*, https://dkratzert.de/finalcif.html.
9. a) M. Douglas, N. M. Kroll, *Ann. Phys.*, **1974**, *82*, 89. b) B. A. Hess, *Phys. Rev. A*, **1985**, *32*, 756. c) D. A. Pantazis, X.-Y. Chen, C. R. Landis, F. Neese, *J. Chem. Theor. Comput.*, **2008**, *4*, 908.
10. a) Neese, F. Software update: the ORCA program system, version 6.0, WIRES *Comput. Molec. Sci.* **2025**, 15, e70019, DOI: 10.1002/wcms.7019. b) Neese, F., *J. Comp. Chem.* **2003** *24*, 1740-1747, DOI: 10.1002/jcc.10318; c) Neese, F.; Wennmohs, F.; Hansen, A.; Becker, U. Chem. Phys. 2009 356, 98-109; d) Grimme, S.; Antony, J.; Ehrlich, S.; Krieg, H. *J. Chem. Phys.* **2010** *132*, 154104; e) Grimme, S.; Ehrlich, S.; Goerigk, L. *J. Comput. Chem.* **2011** *32*, 1456-1465; f) Helmich-Paris, B.; de Souza, B.; Neese, F.; Izsák, R. *J. Chem. Phys.* **2021** *155*, 104109; g) Neese, F. J. *Comp. Chem.* **2022** *44*, 381.

Author contributions

V. M. conceived an idea, led and managed the project, acquired the funding, and conducted experiments. N. L. measured all X-ray crystal structures.

NMR spectra

Bis(trifluoromethyl)copper(III) hydroxide tetramer 3

^{1}H NMR (400 MHz, MeCN-d$_3$)

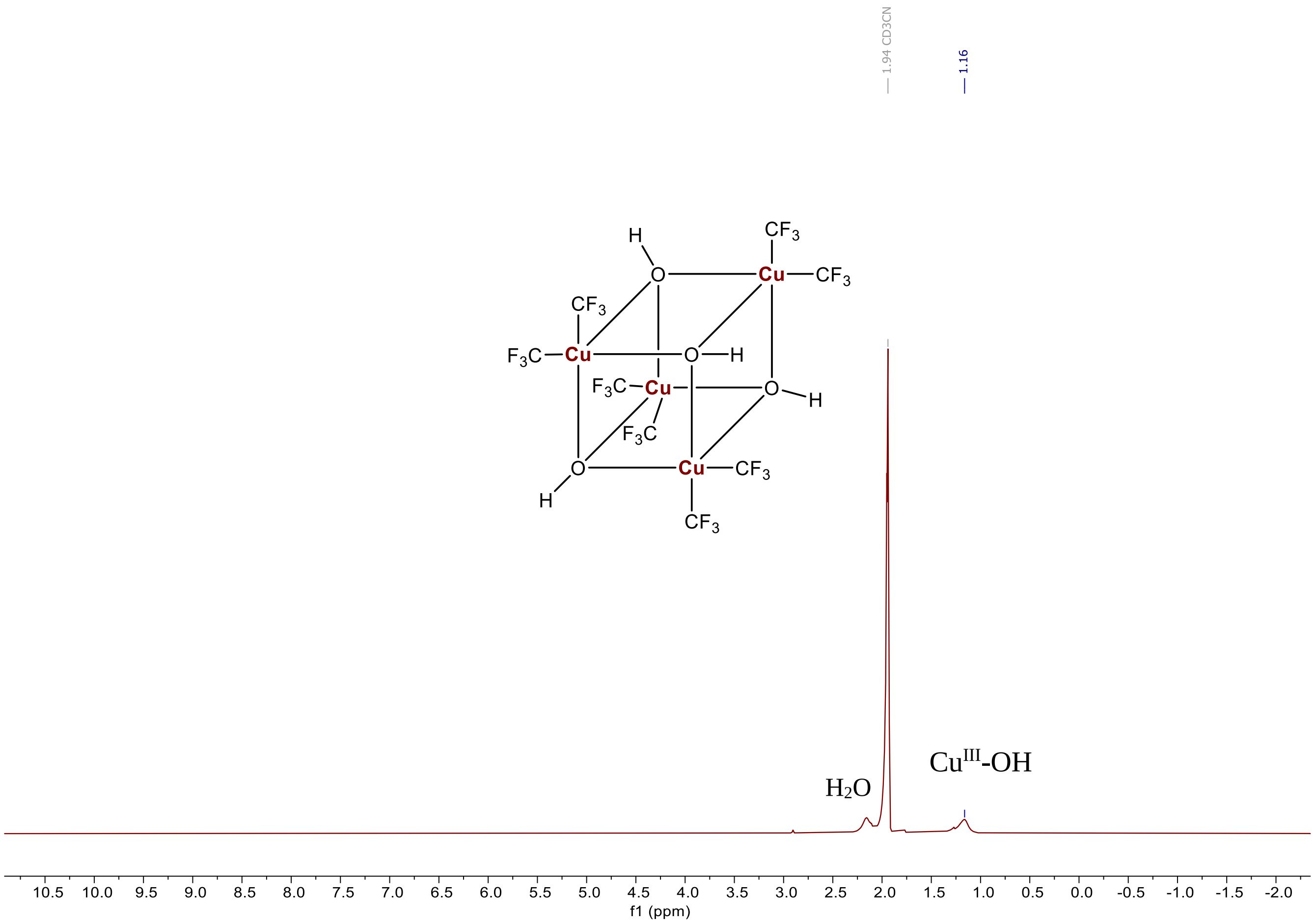

^{19}F NMR (376 MHz, MeCN-d_3)

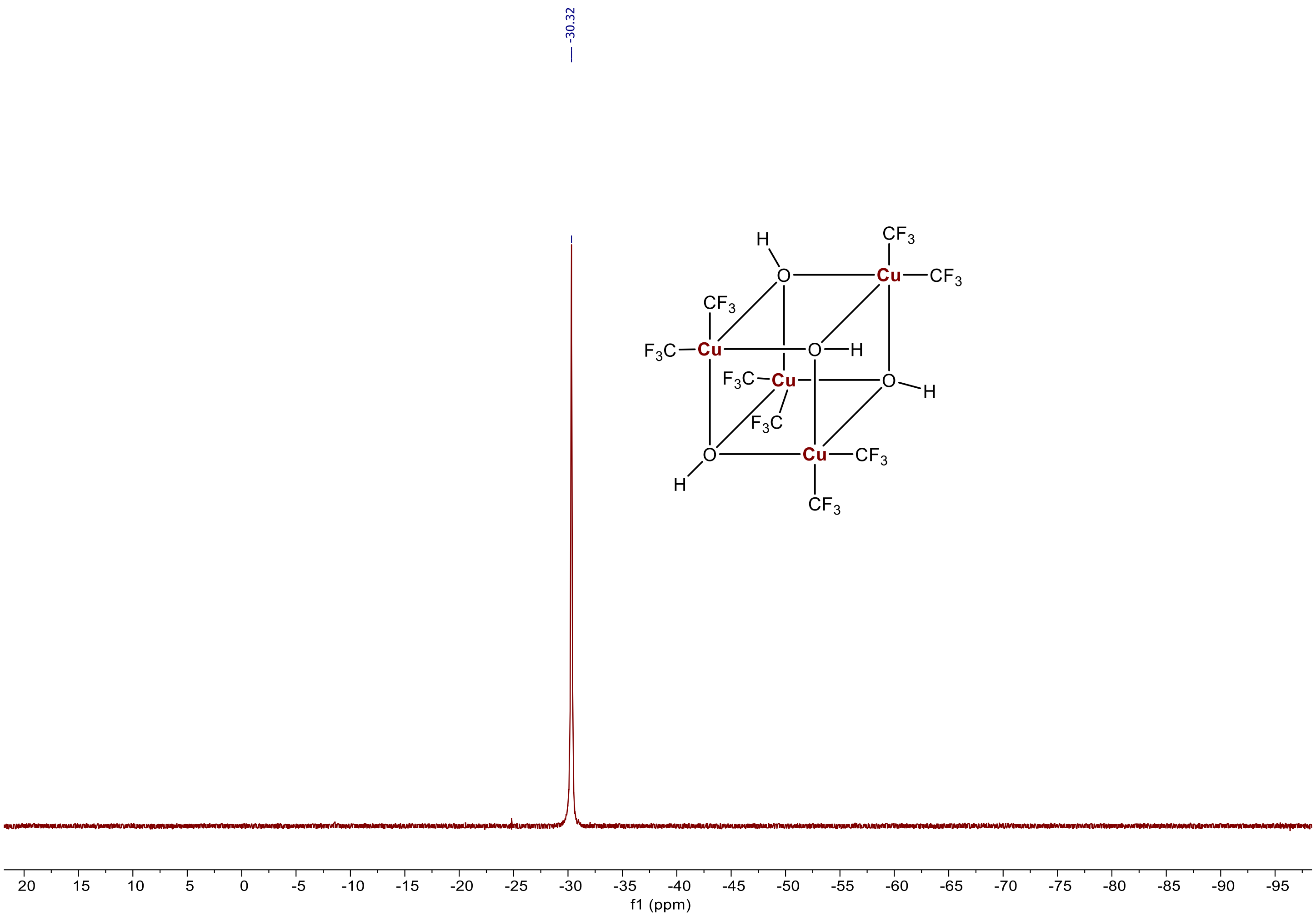

^{19}F NMR (376 MHz, $CDCl_3$)

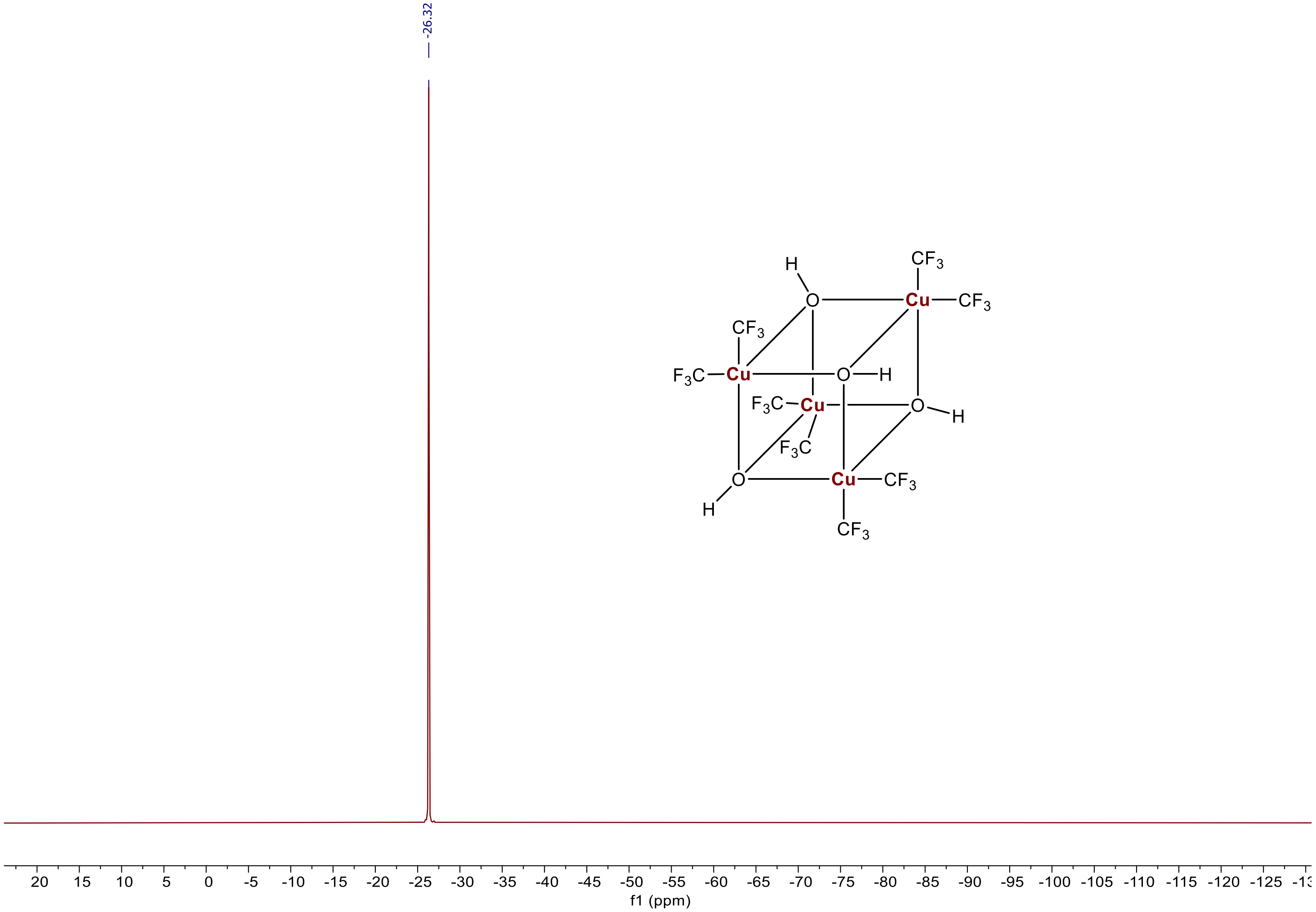

^{19}F NMR (376 MHz, acetone-d_6)

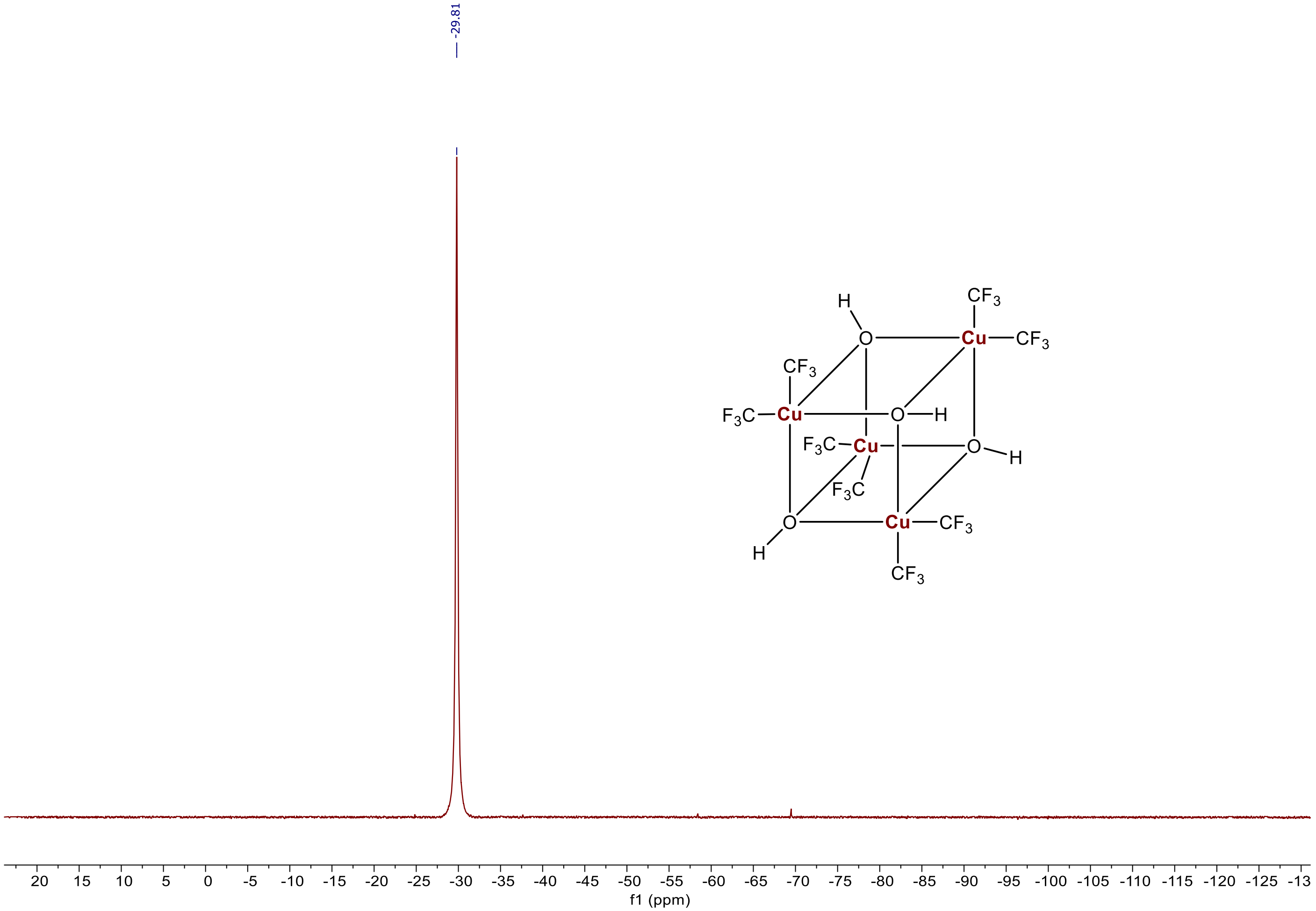

^{19}F NMR (376 MHz, D_2O)

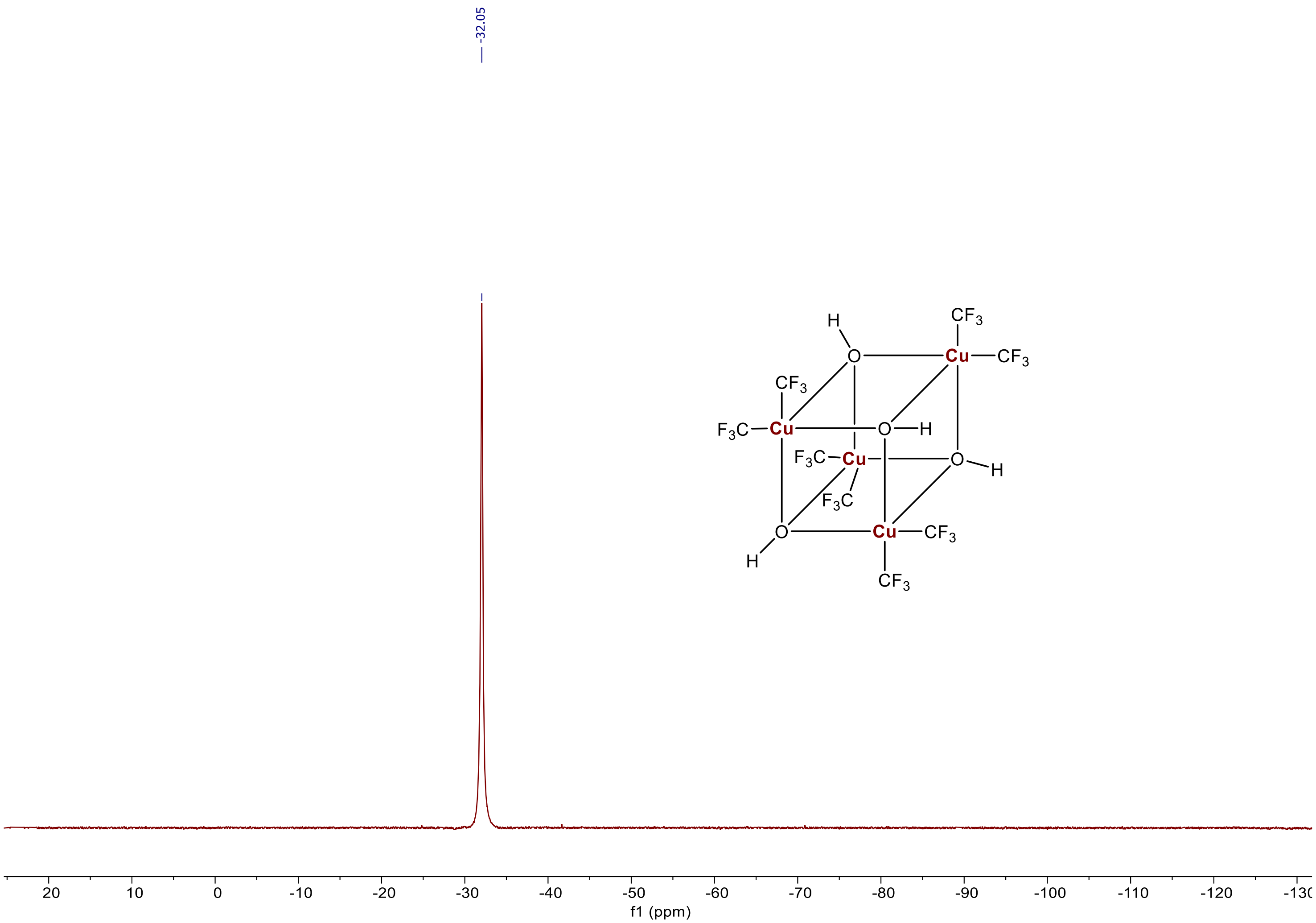

***Bis(trifluoromethyl)copper(III) pyridine-2-carboxylate* 4**

^{1}H NMR (400 MHz, MeCN-d_3)

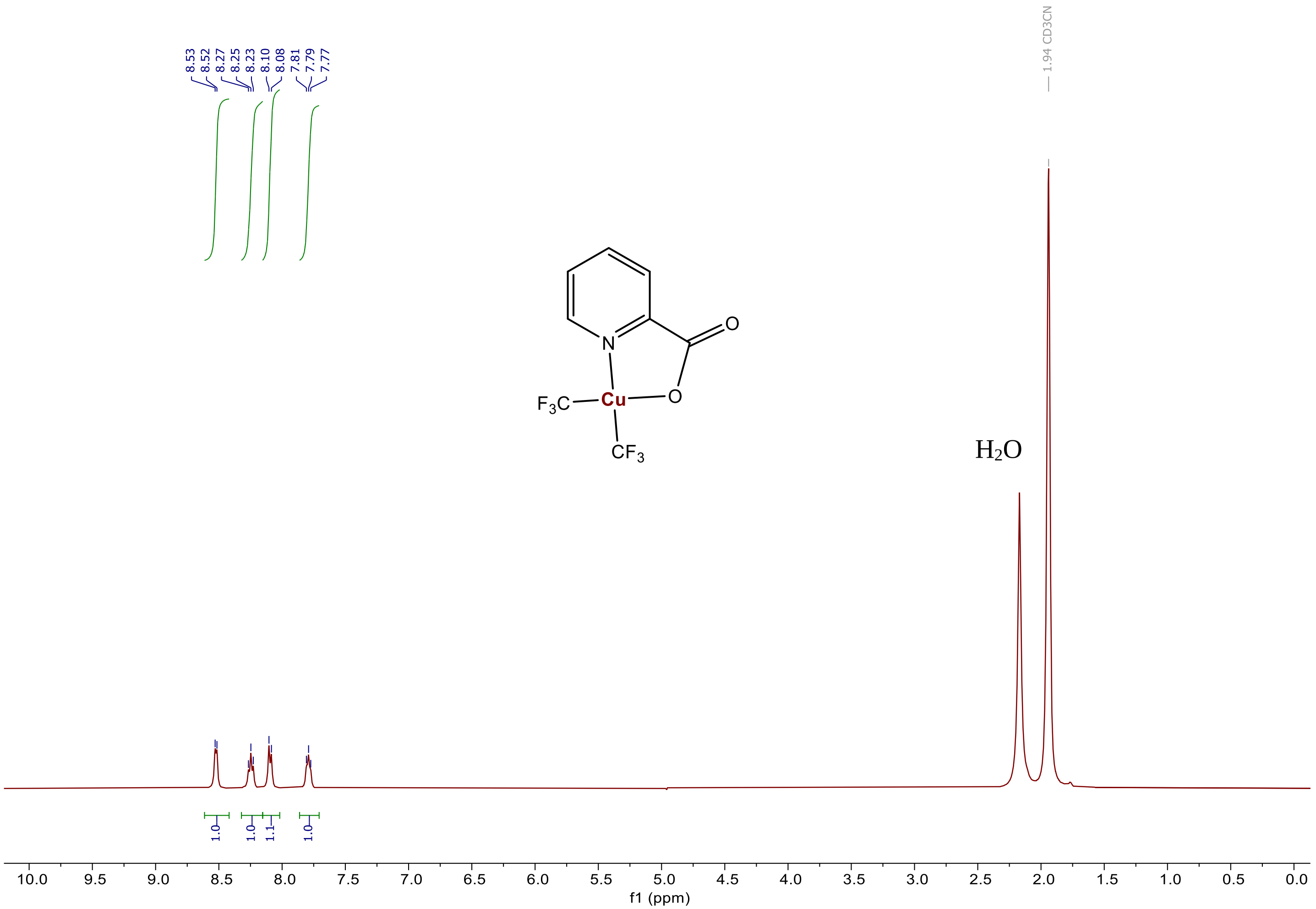

^{13}C NMR (101 MHz, MeCN-d_3)

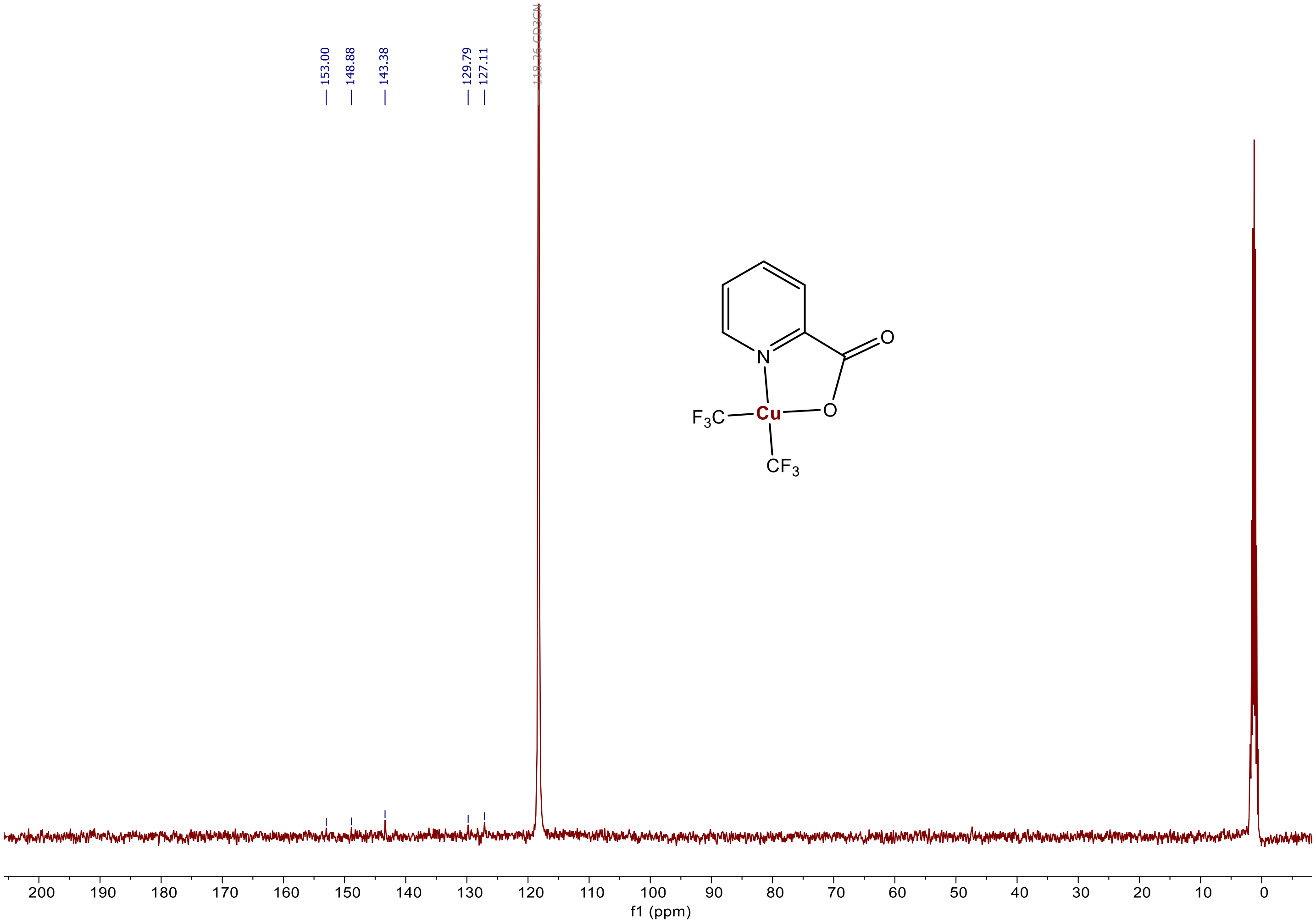

^{19}F NMR (282 MHz, MeCN-d_3)

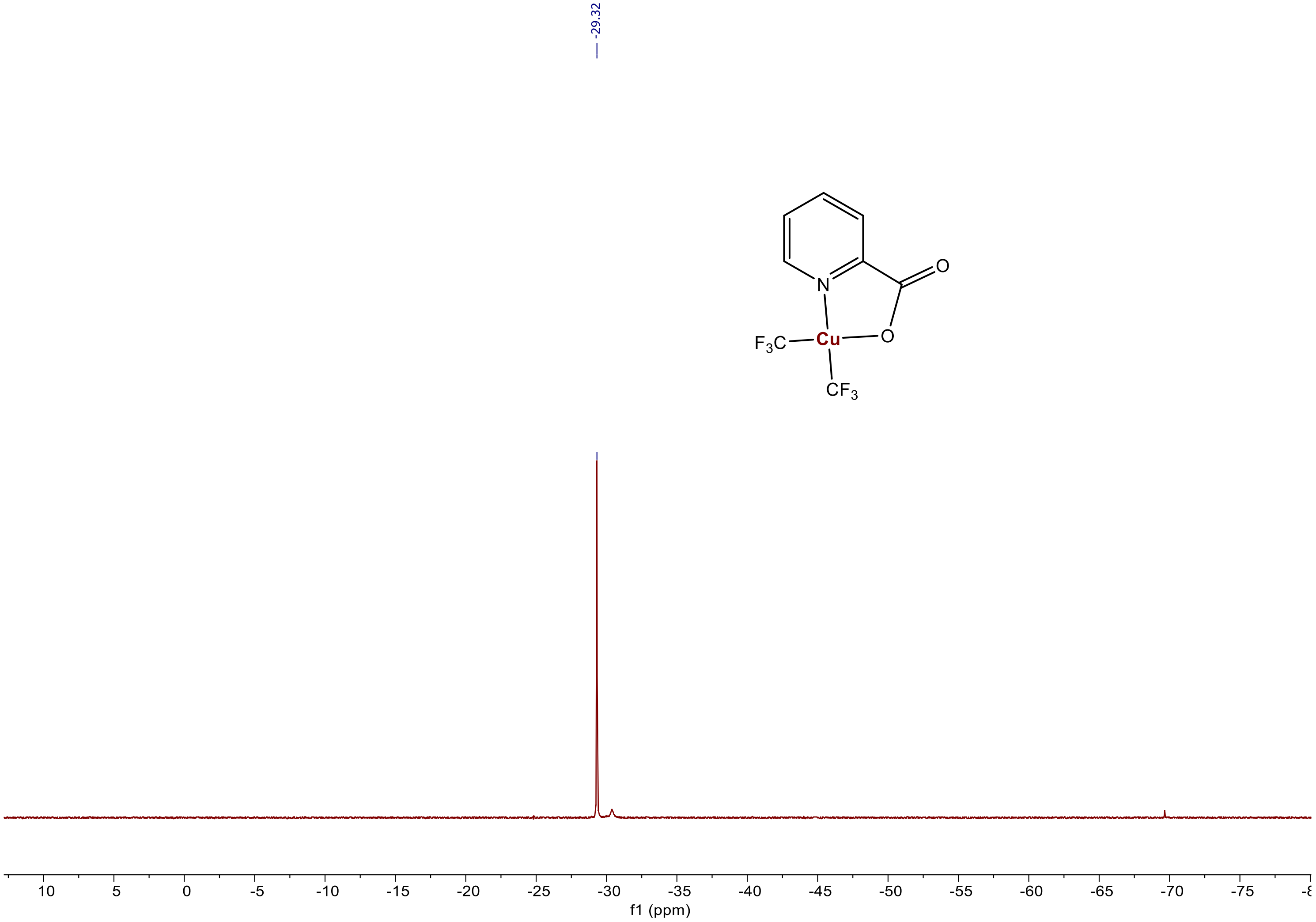

(Acetato)(bis(trifluoromethyl)copper(III) 5

^{1}H NMR (400 MHz, MeCN-d_3)

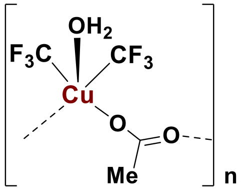

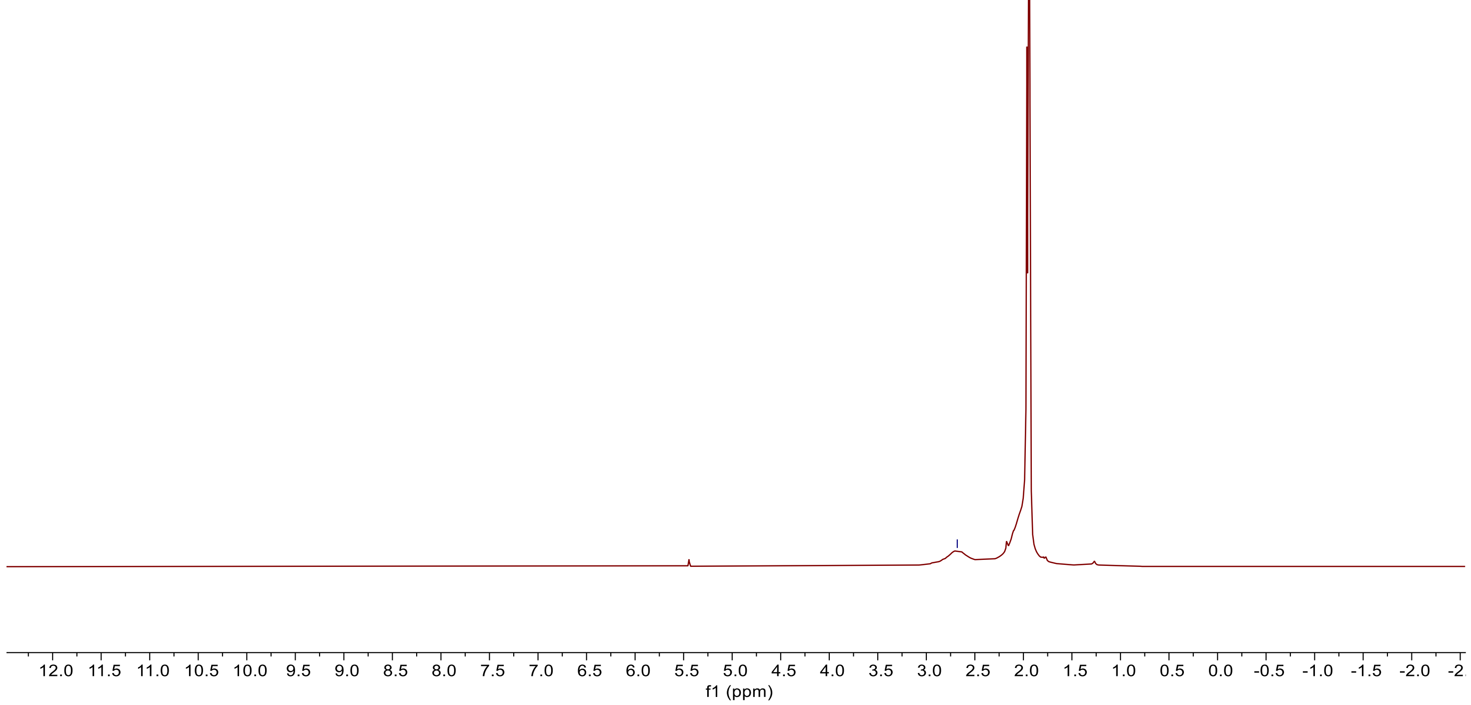

^{19}F NMR (376 MHz, MeCN-d_3)

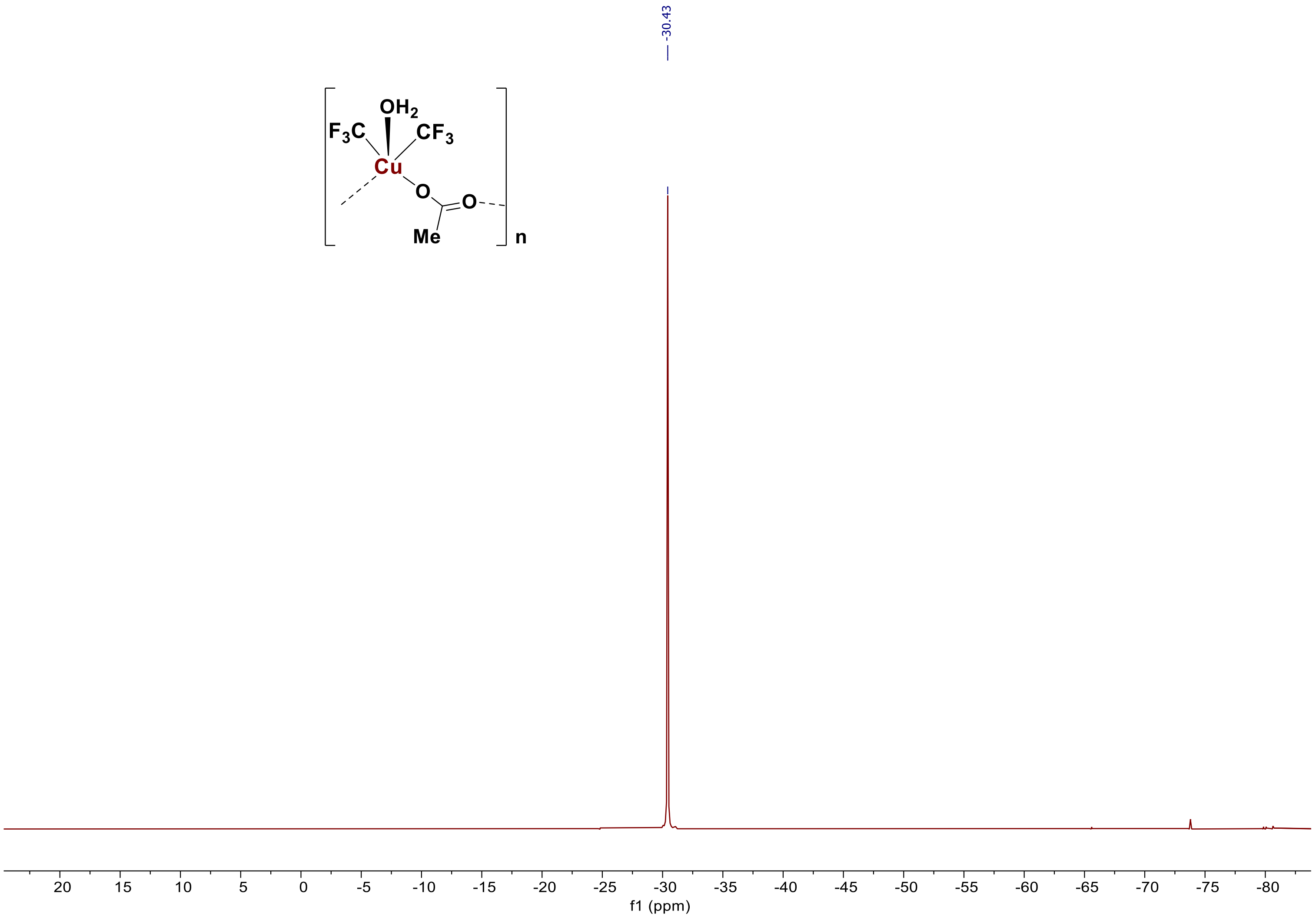

***(Acetylacetonato)bis(trifluoromethyl)copper(III)* 6**

^{1}H NMR (400 MHz, $CDCl_3$)

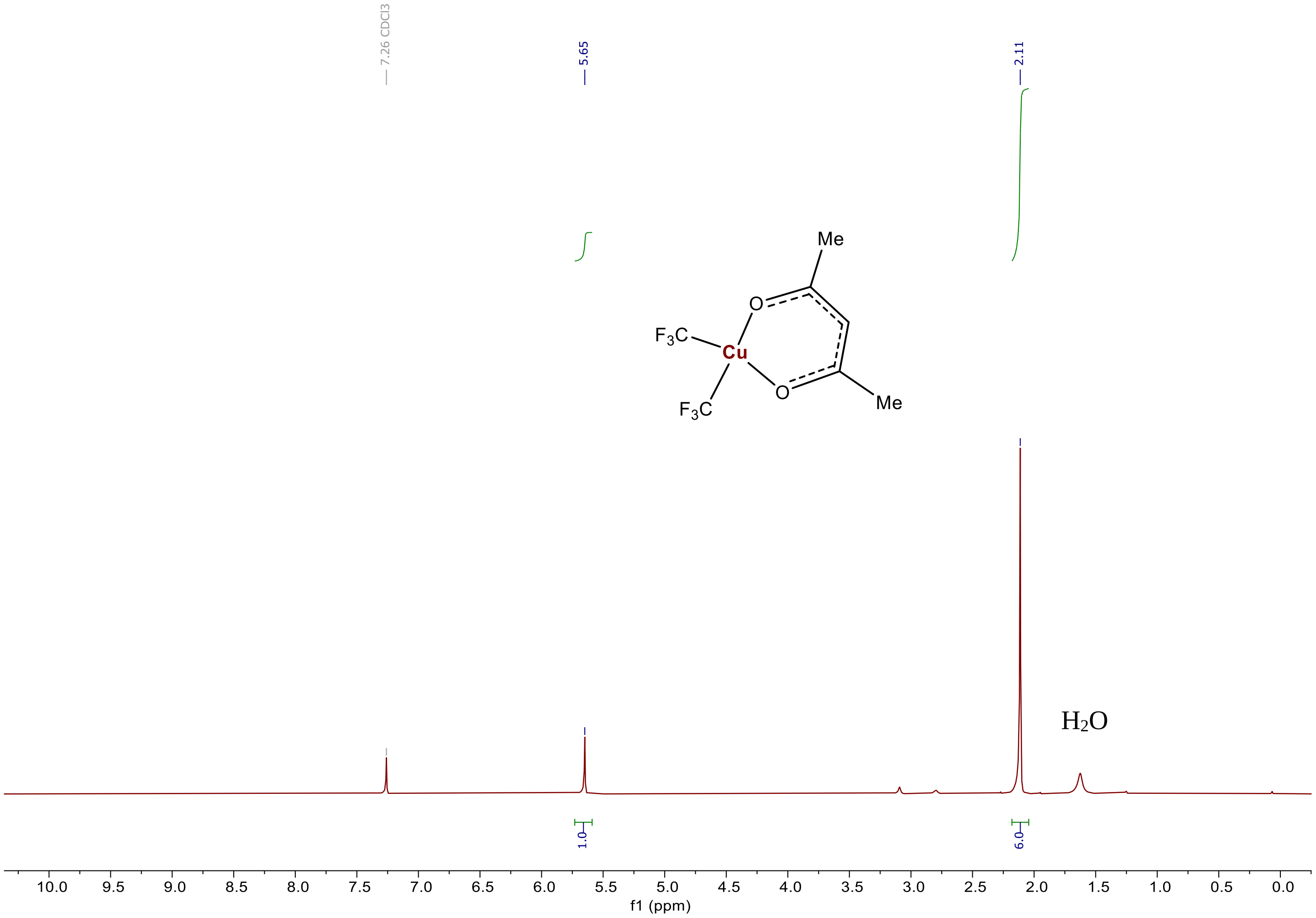

^{13}C NMR (101 MHz, $CDCl_3$)

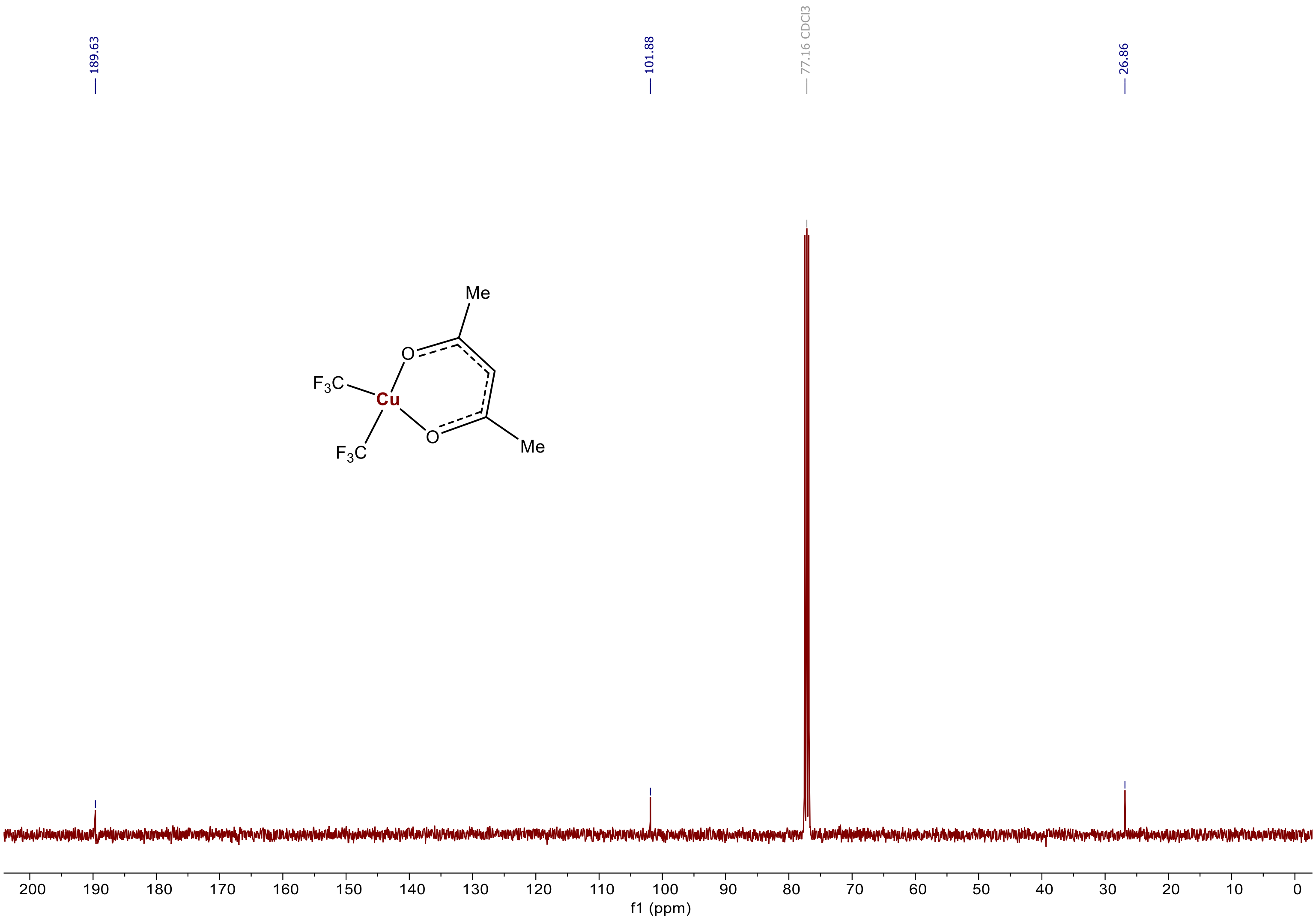

^{19}F NMR (376 MHz, $CDCl_3$)

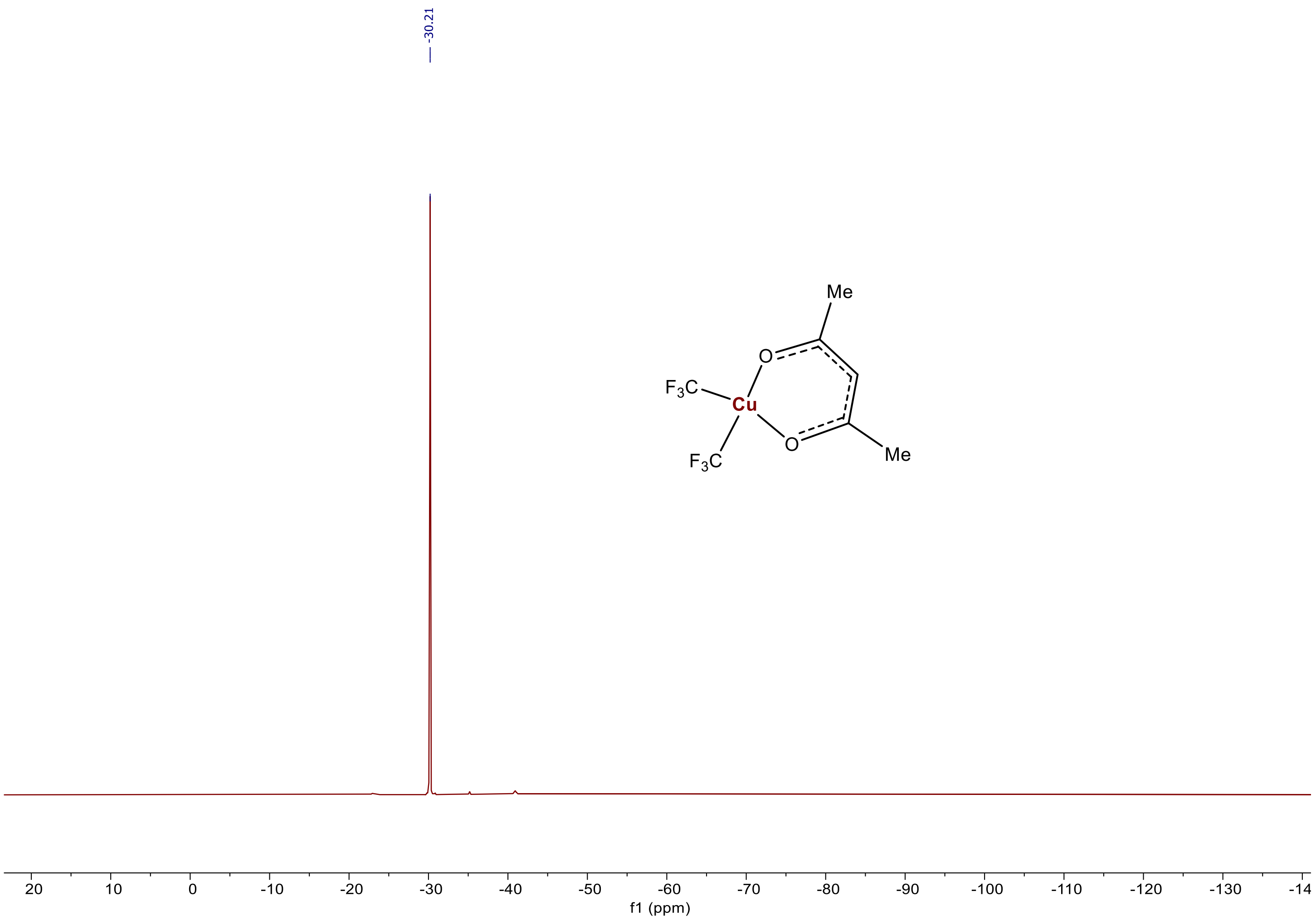

(1,3-Diphenylpropan-1,3-dionato)bis(trifluoromethyl)copper(III) **7**

^{1}H NMR (400 MHz, $CDCl_3$)

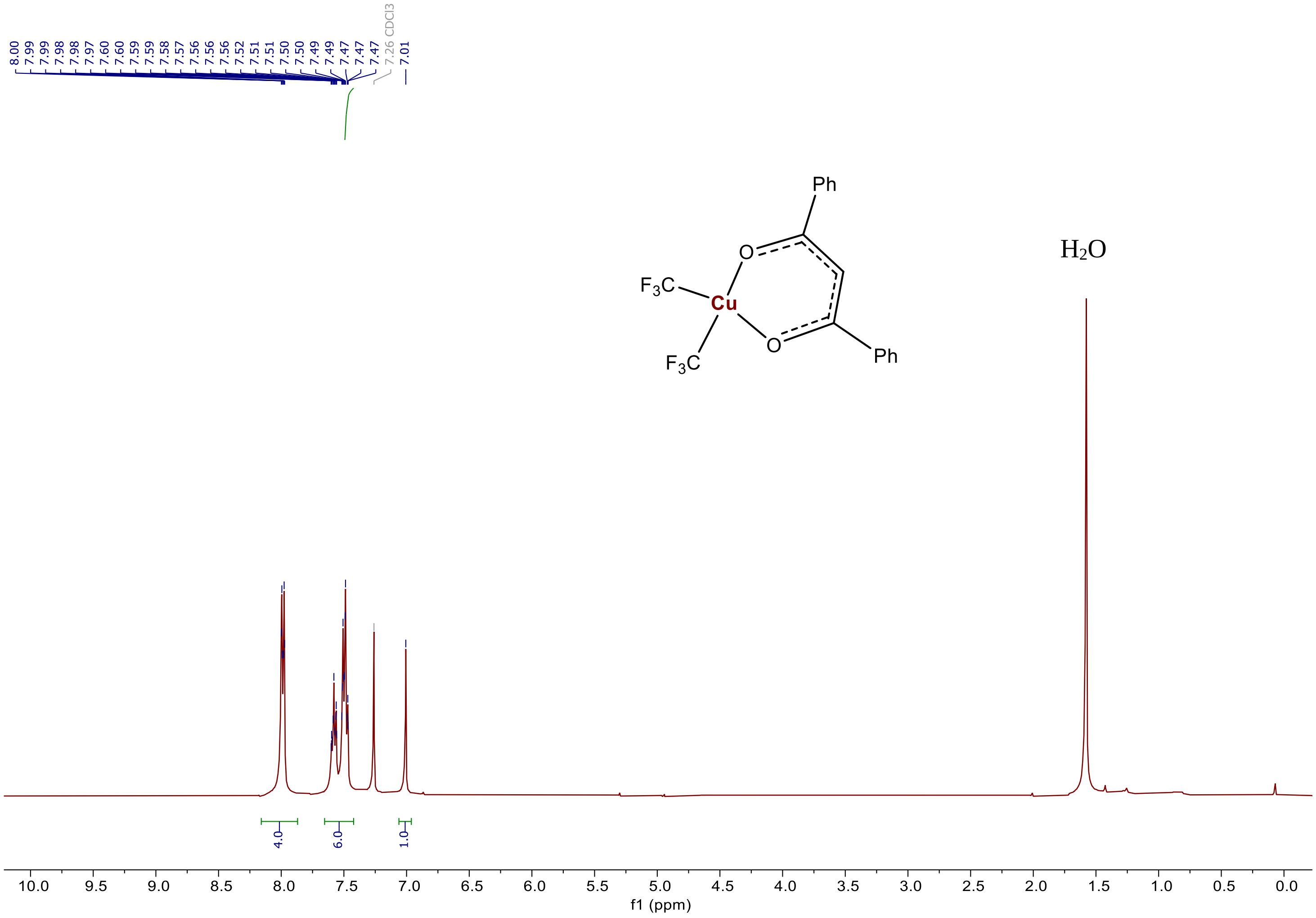

^{13}C NMR (101 MHz, $CDCl_3$)

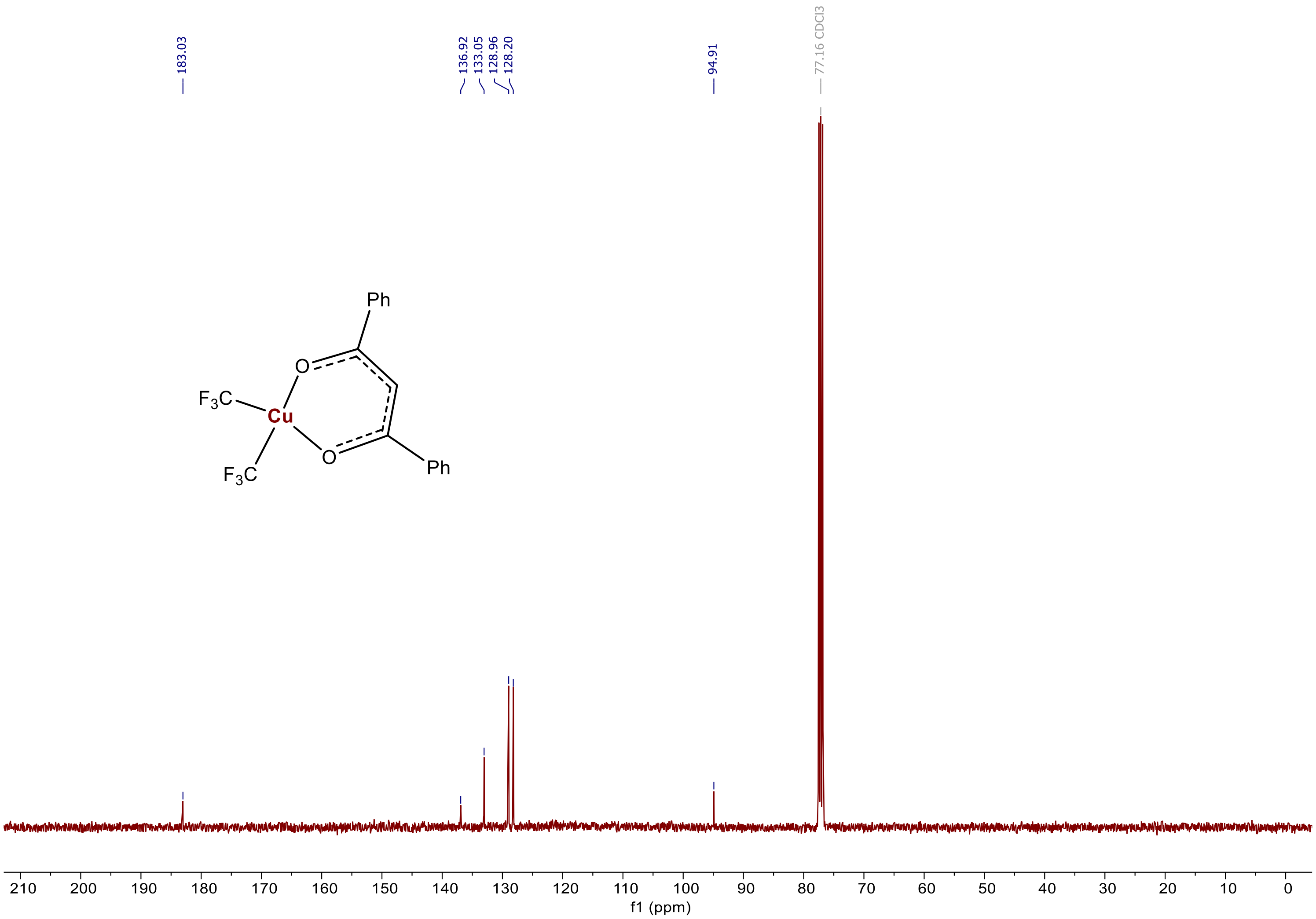

^{19}F NMR (376 MHz, $CDCl_3$)

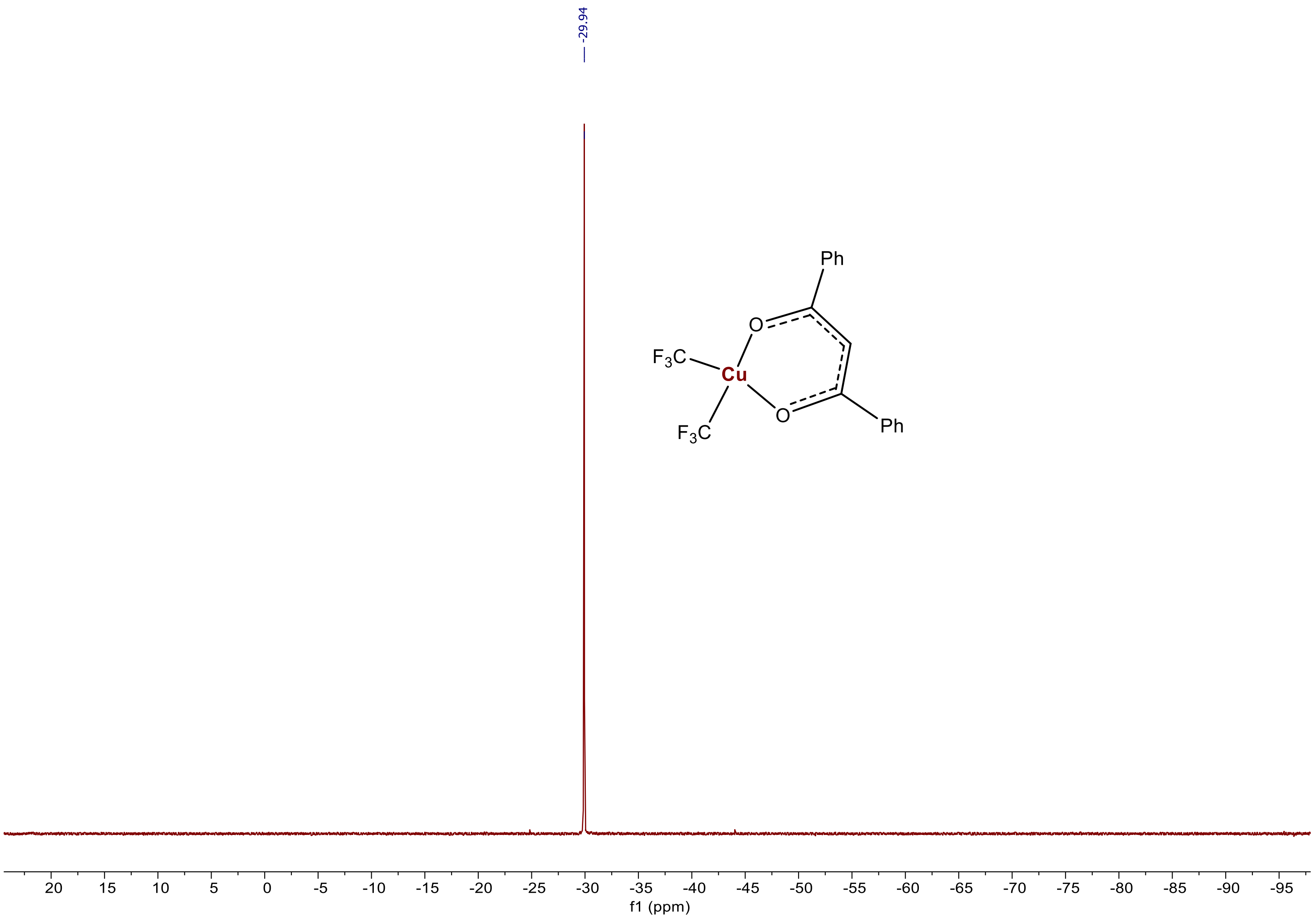

(η^2-Thiobenzamido)bis(trifluoromethyl)copper(III) 8

^{1}H NMR (401 MHz, $CDCl_3$)

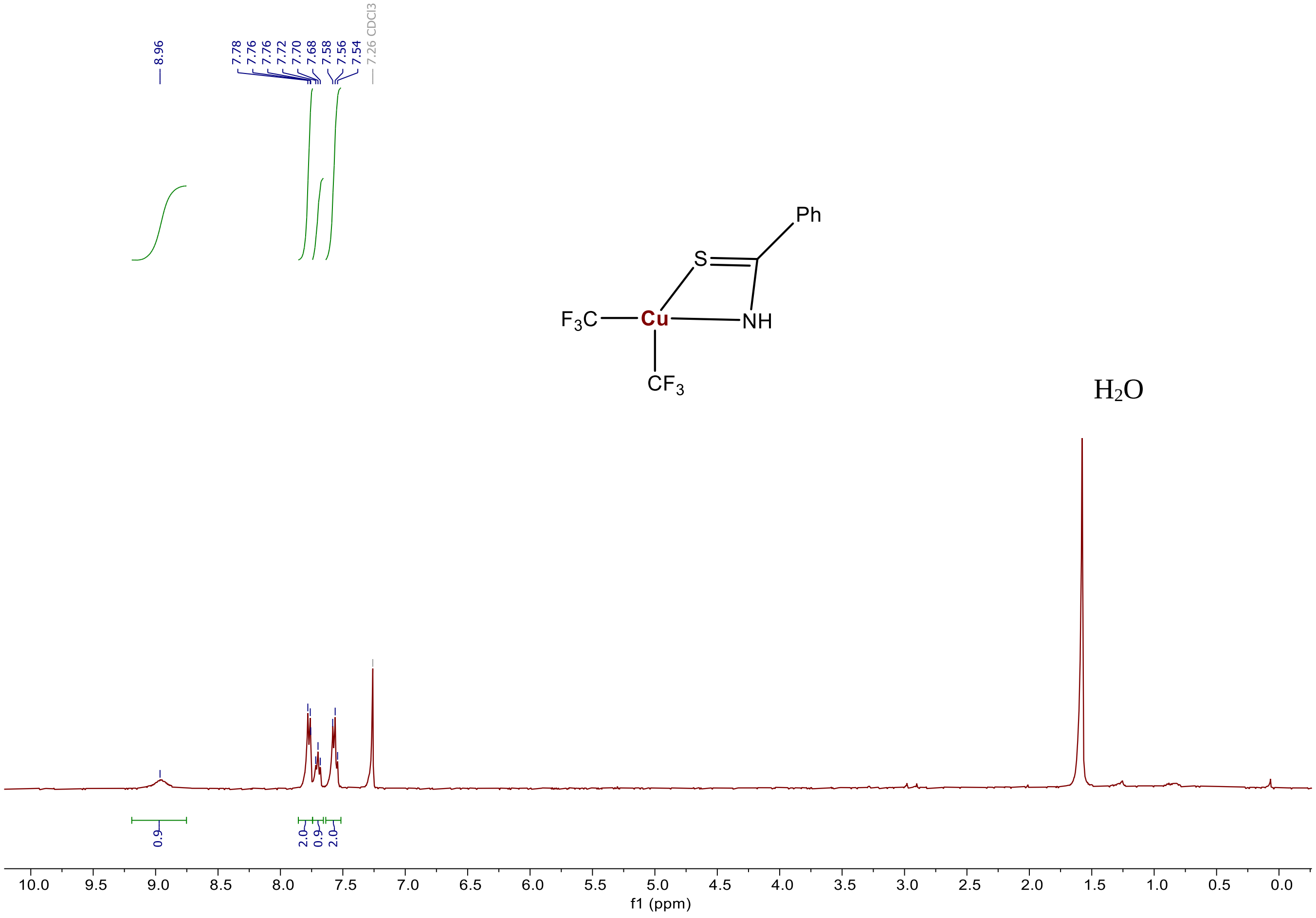

^{13}C NMR (101 MHz, $CDCl_3$)

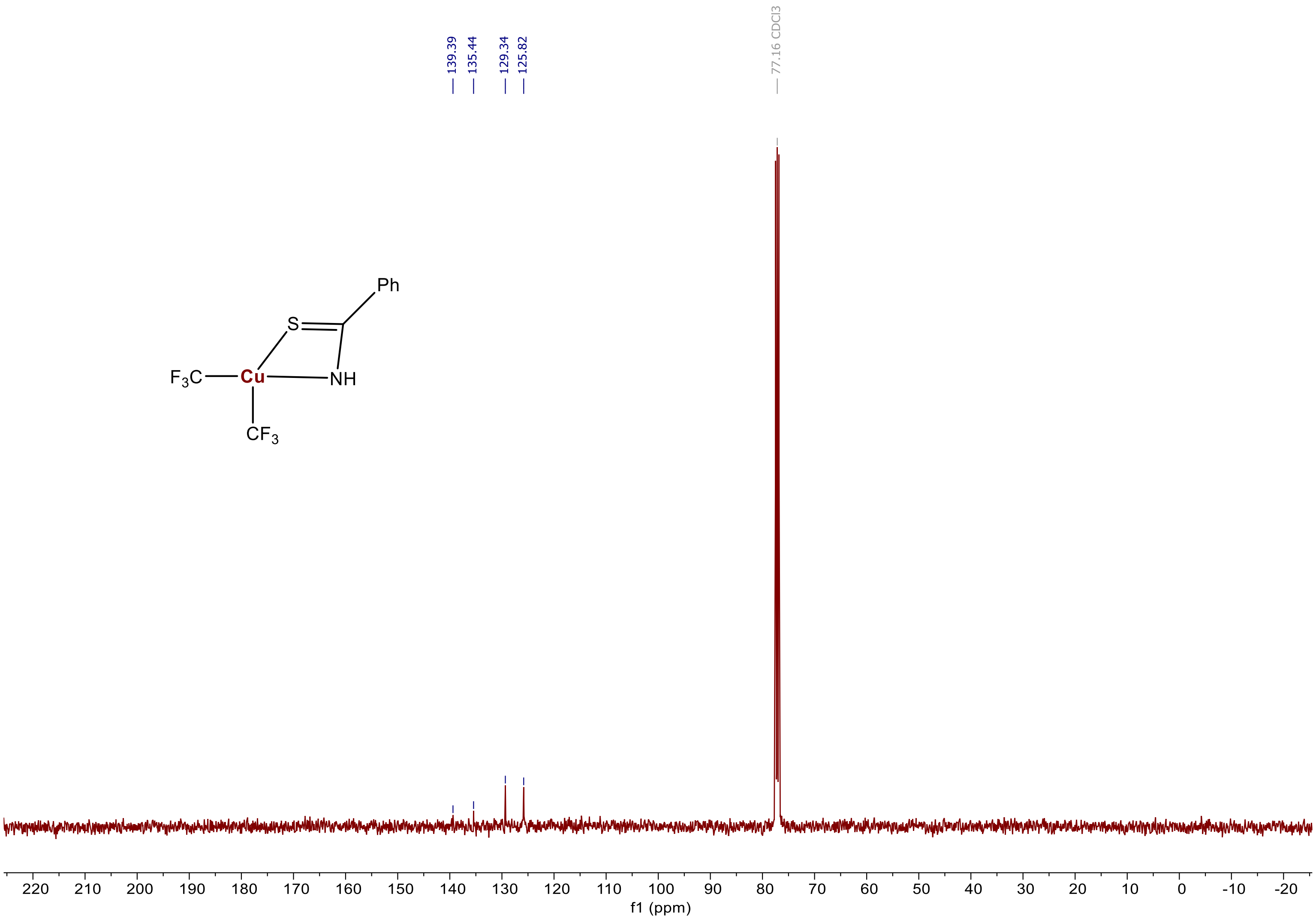

^{19}F NMR (376 MHz, $CDCl_3$)

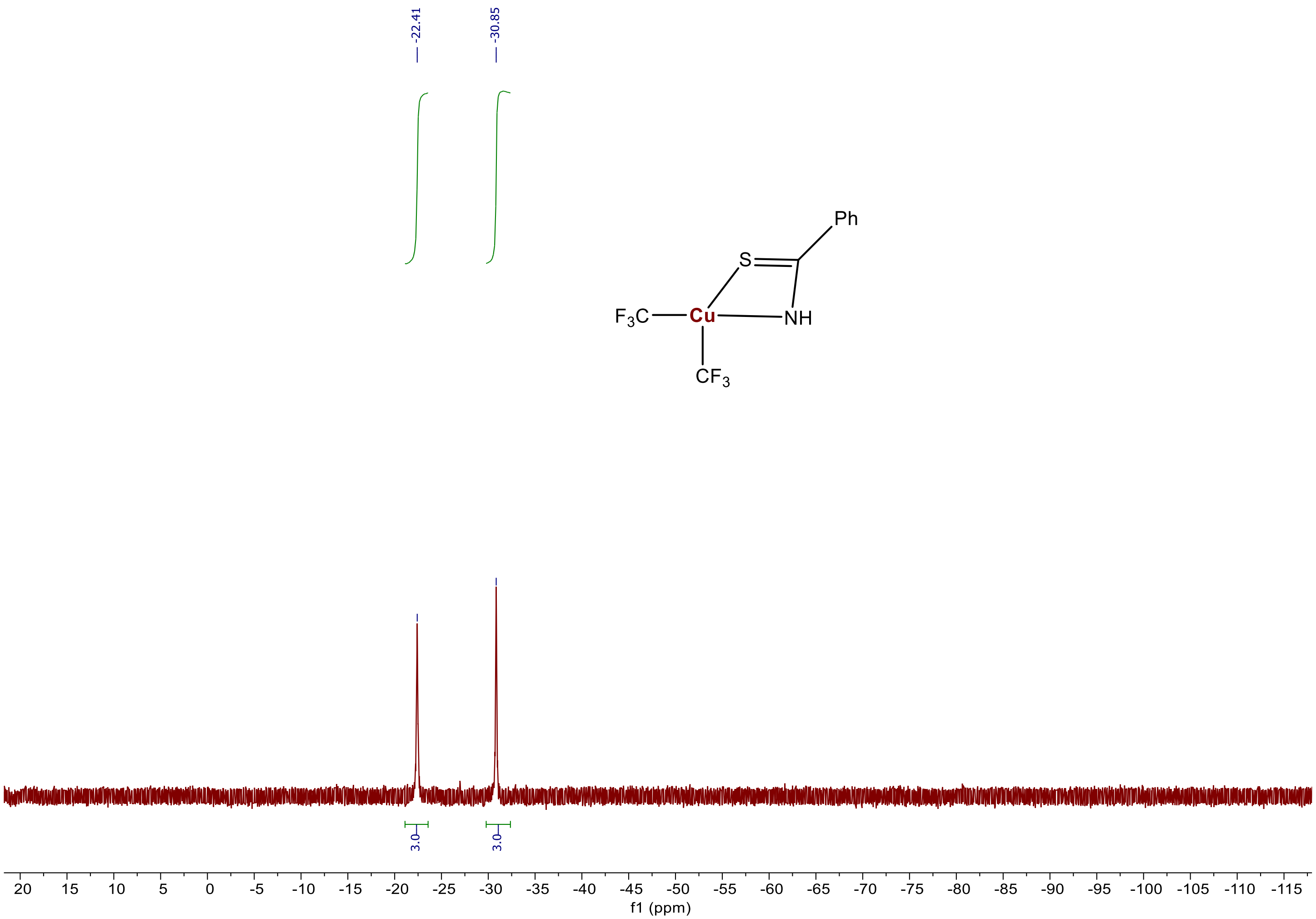

Trifluoromethylbenzene 9 (reaction mixture)

^{19}F NMR (376 MHz, $CDCl_3$)

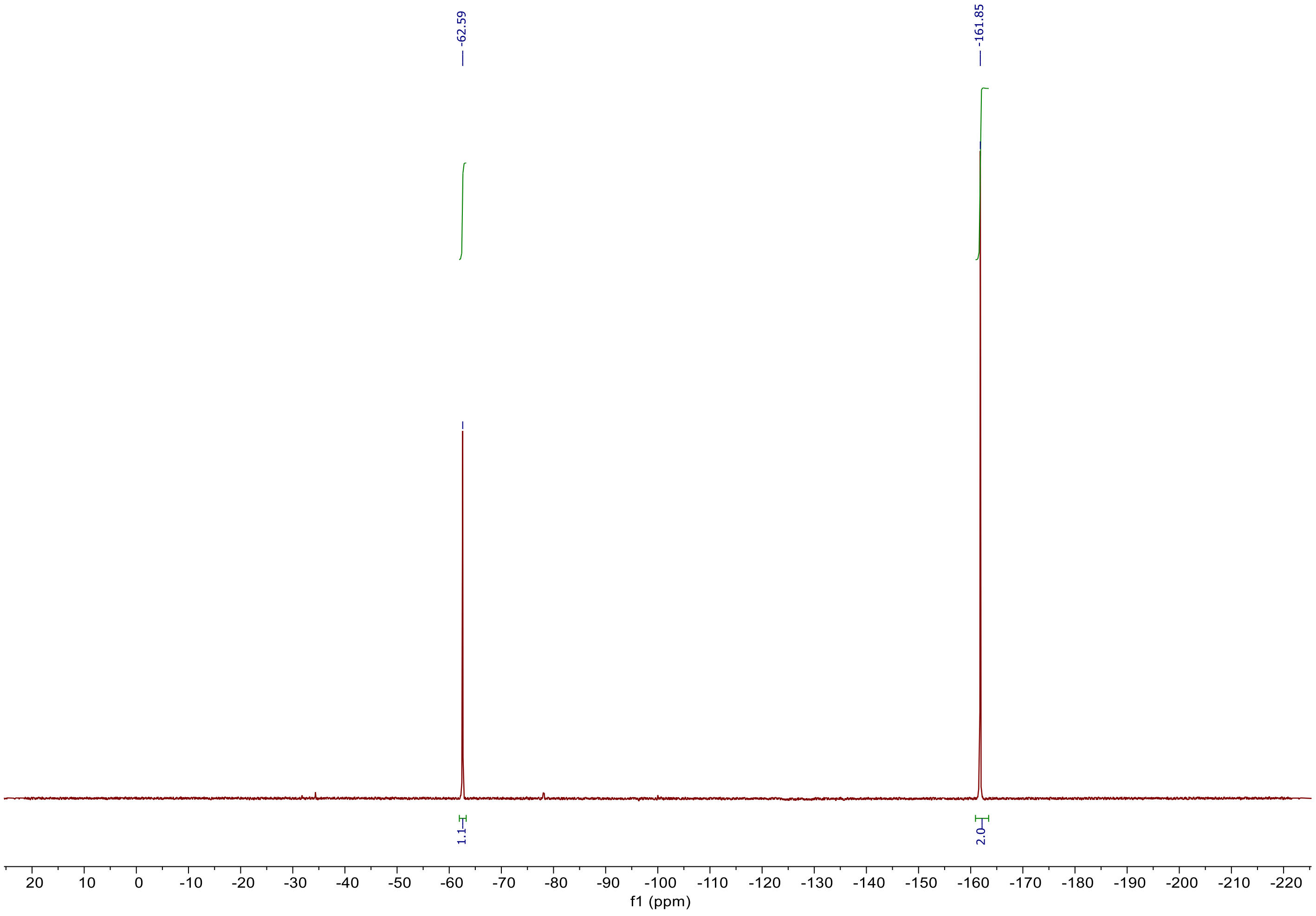

1,3,5-Trimethoxy-2-(trifluoromethyl)benzene 10

^1H NMR (400 MHz, $CDCl_3$)

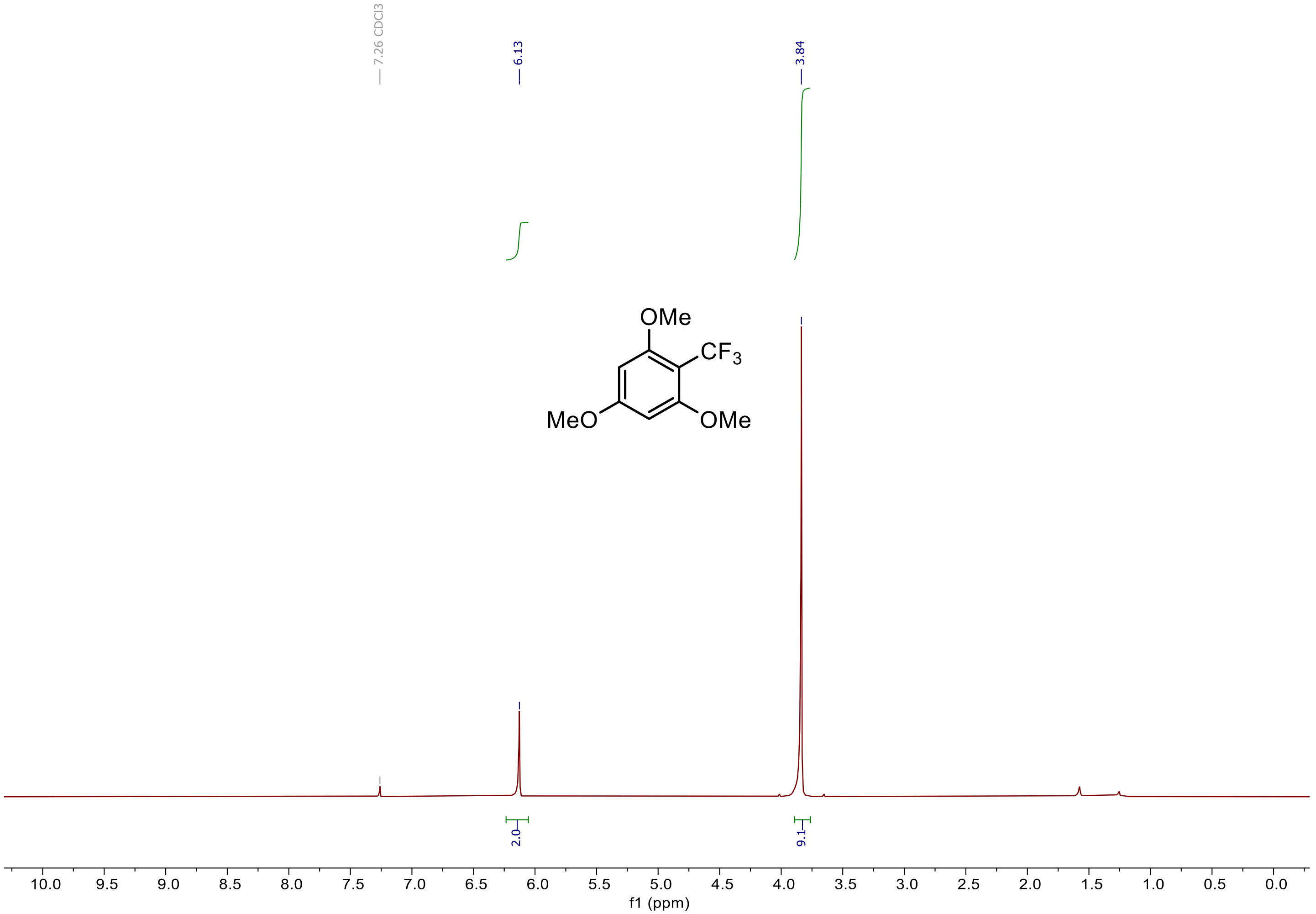

^{13}C NMR (101 MHz, $CDCl_3$)

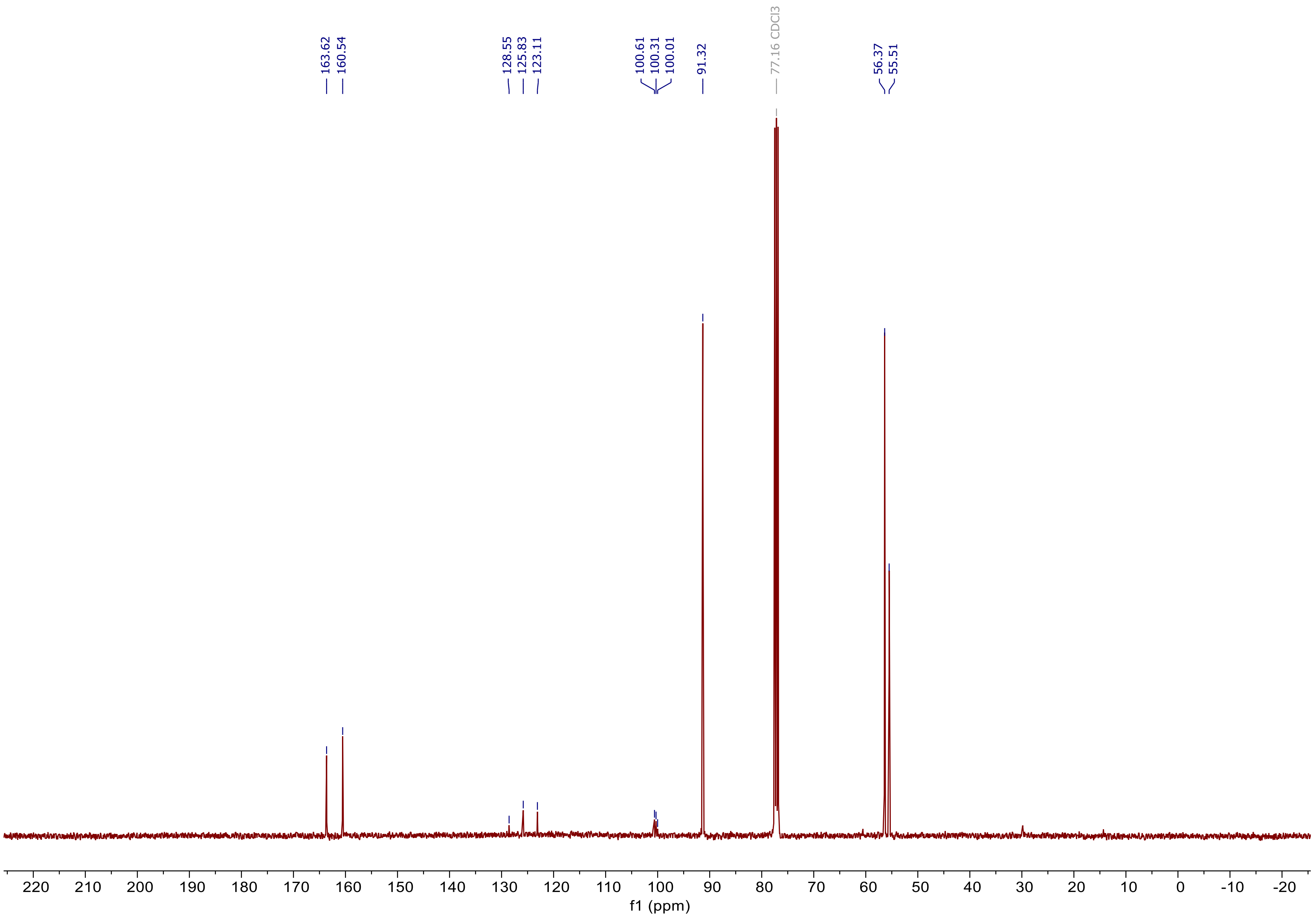

^{19}F NMR (376 MHz, $CDCl_3$)

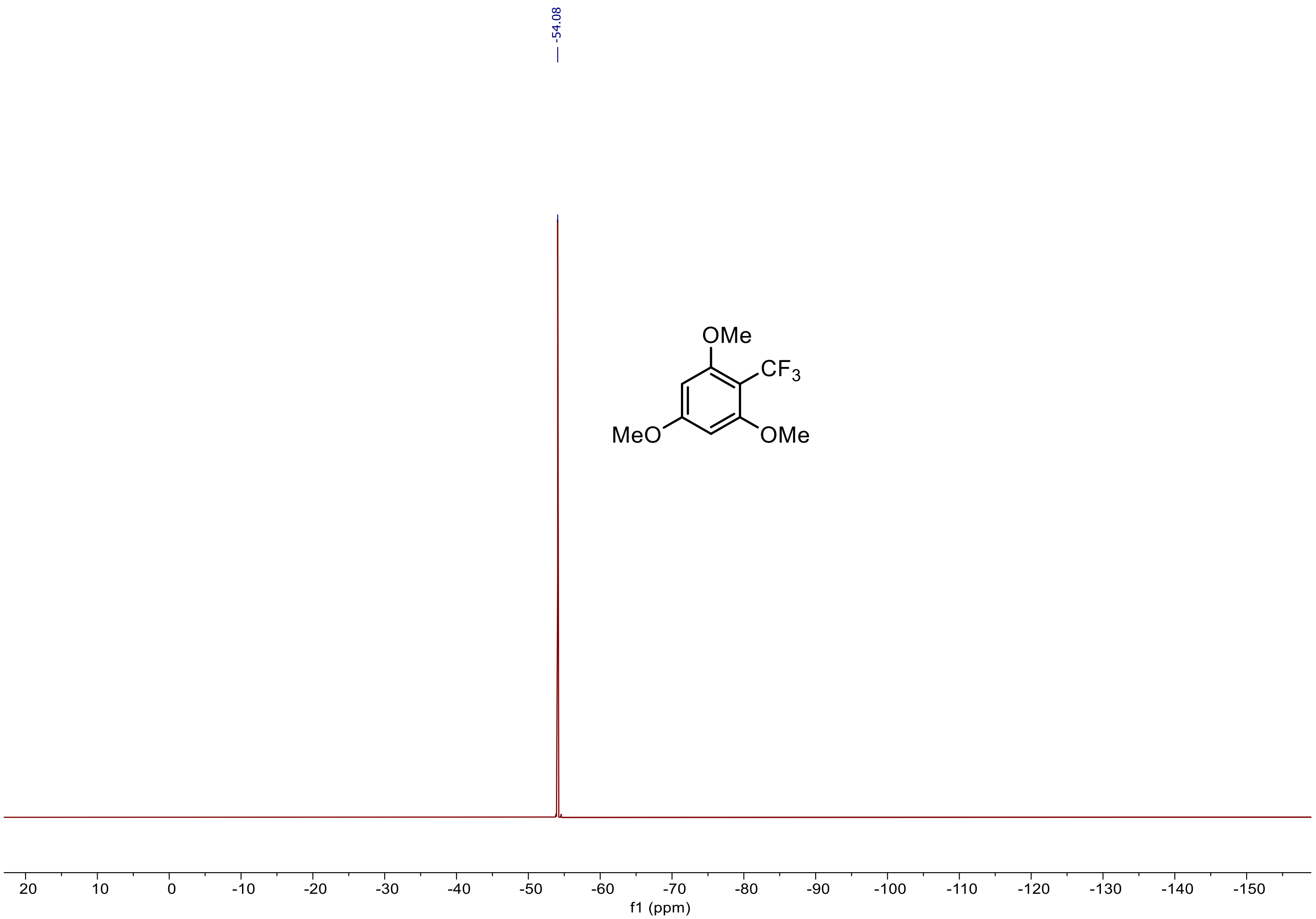

Supplement: Supplementary file 1 — Supporting file 1: The authors have cited additional references within the Supporting Information [41, 42, 43, 44, 45, 46, 47, 48, 49, 50, 51, 52, 53, 54, 55, 56]. Deposition Numbers 2481836 (for 3), 2481839 (for 4), 2481840 (for 5), 2481838 (for 8) contain the supplementary crystallographic data for this paper. These data are provided free of charge by the joint Cambridge Crystallographic Data Centre and Fachinformationszentrum Karlsruhe Access Structures service (https://www.ccdc.cam.ac.uk/structures). [file CHEM-32-e03417-s001.pdf]
