# Supplementary material for: Unprecedented Formation of a Formally Cu(III) Trifluoromethyl Hydroxide Tetramer
Source: Chemistry. 2025 Dec 18;32(5):e03417. doi: 10.1002/chem.202503417 (PMC12865140; doi:10.1002/chem.202503417)

checkCIF/PLATON report

Structure factors have been supplied for datablock(s) MV022

THIS REPORT IS FOR GUIDANCE ONLY. IF USED AS PART OF A REVIEW PROCEDURE FOR PUBLICATION, IT SHOULD NOT REPLACE THE EXPERTISE OF AN EXPERIENCED CRYSTALLOGRAPHIC REFEREE.

No syntax errors found. CIF dictionary Interpreting this report

Datablock: MV022

Bond precision: F- C = 0.0098 A Wavelength=0.71073

Cell:	a=23.0647(7)	b=23.0647(7)	c=10.5677(4)
	alpha=90	beta=90	gamma=90
Temperature:	150 K		

	Calculated	Reported
Volume	5621.8(4)	5621.8(4)
Space group	I -4	I-4
Hall group	I -4	I -4
Moiety formula	C8 H4 Cu4 F24 O4	C8 H4 Cu4 F24 O4
Sum formula	C8 H4 Cu4 F24 O4	C8 H4 Cu4 F24 O4
Mr	874.31	874.27
Dx,g cm-3	2.582	2.582
Z	10	10
Mu (mm-1)	3.945	3.945
F000	4160.0	4160.0
F000’	4177.12	
h,k,lmax	27,27,12	27,27,12
Nref	5178[2743]	5170
Tmin,Tmax	0.339,0.520	0.451,0.745
Tmin’	0.118	

Correction method= # Reported T Limits: Tmin=0.451 Tmax=0.745
AbsCorr = MULTI-SCAN

Data completeness= 1.88/1.00 Theta(max)= 25.365

R(reflections)= 0.0285(4858) wR2(reflections)= 0.0689(5170)

S = 1.150 Npar= 452

The following ALERTS were generated. Each ALERT has the format
test-name_ALERT_alert-type_alert-level.
Click on the hyperlinks for more details of the test.

Alert level C

```
PLAT090_ALERT_3_C Poor Data / Parameter Ratio (Zmax > 18) ........       6.06 Note
PLAT213_ALERT_2_C Atom F17             has ADP max/min Ratio .....        3.1 prolat
PLAT790_ALERT_4_C Centre of Gravity not Within Unit Cell: Resd.  #          1 Note
              C8 H4 Cu4 F24 O4
```

Alert level G

```
PLAT003_ALERT_2_G Number of Uiso or U(i,j)  Restrained non-H-Atoms          6 Report
PLAT007_ALERT_5_G Number of Unrefined Donor-H Atoms .............           5 Report
              H1    H2    H3    H4    H5
PLAT083_ALERT_2_G SHELXL Second Parameter in WGHT  Unusually Large      24.50 Why ?
PLAT186_ALERT_4_G The CIF-Embedded .res File Contains ISOR Records          2 Report
PLAT242_ALERT_2_G Low    'MainMol' Ueq as Compared to Neighbors of         C1 Check
PLAT242_ALERT_2_G Low    'MainMol' Ueq as Compared to Neighbors of         C2 Check
PLAT242_ALERT_2_G Low    'MainMol' Ueq as Compared to Neighbors of         C3 Check
PLAT242_ALERT_2_G Low    'MainMol' Ueq as Compared to Neighbors of         C4 Check
PLAT242_ALERT_2_G Low    'MainMol' Ueq as Compared to Neighbors of         C5 Check
PLAT242_ALERT_2_G Low    'MainMol' Ueq as Compared to Neighbors of         C6 Check
PLAT242_ALERT_2_G Low    'MainMol' Ueq as Compared to Neighbors of         C7 Check
PLAT242_ALERT_2_G Low    'MainMol' Ueq as Compared to Neighbors of         C8 Check
PLAT242_ALERT_2_G Low    'MainMol' Ueq as Compared to Neighbors of         C9 Check
PLAT242_ALERT_2_G Low    'MainMol' Ueq as Compared to Neighbors of        C10 Check
PLAT434_ALERT_2_G Short Inter HL..HL Contact F5       ..F25      .       2.76 Ang.
                                                    x,y,1+z  =      1_556 Check
PLAT794_ALERT_5_G Tentative Bond Valency for Cu1       (III)     .       2.99 Info
PLAT794_ALERT_5_G Tentative Bond Valency for Cu2       (III)     .       2.95 Info
PLAT794_ALERT_5_G Tentative Bond Valency for Cu3       (III)     .       2.97 Info
PLAT794_ALERT_5_G Tentative Bond Valency for Cu4       (III)     .       2.93 Info
PLAT794_ALERT_5_G Tentative Bond Valency for Cu5       (III)     .       2.94 Info
PLAT860_ALERT_3_G Number of Least-Squares Restraints ............          36 Note
PLAT910_ALERT_3_G Missing FCF Reflection(s) Below Theta(Min)[Deg]=       2.12 Note
                1  1  0,   0  2  0,
PLAT969_ALERT_5_G The 'Henn et al.' R-Factor-gap value ..........      1.926 Note
              Predicted wR2: Based on SigI**2  3.57 or SHELX Weight  5.99
```

0 **ALERT level A** = Most likely a serious problem - resolve or explain
0 **ALERT level B** = A potentially serious problem, consider carefully
3 **ALERT level C** = Check. Ensure it is not caused by an omission or oversight
23 **ALERT level G** = General information/check it is not something unexpected

0 ALERT type 1 CIF construction/syntax error, inconsistent or missing data
14 ALERT type 2 Indicator that the structure model may be wrong or deficient
3 ALERT type 3 Indicator that the structure quality may be low
2 ALERT type 4 Improvement, methodology, query or suggestion
7 ALERT type 5 Informative message, check

It is advisable to attempt to resolve as many as possible of the alerts in all categories. Often the minor alerts point to easily fixed oversights, errors and omissions in your CIF or refinement strategy, so attention to these fine details can be worthwhile. In order to resolve some of the more serious problems it may be necessary to carry out additional measurements or structure refinements. However, the purpose of your study may justify the reported deviations and the more serious of these should normally be commented upon in the discussion or experimental section of a paper or in the "special_details" fields of the CIF. checkCIF was carefully designed to identify outliers and unusual parameters, but every test has its limitations and alerts that are not important in a particular case may appear. Conversely, the absence of alerts does not guarantee there are no aspects of the results needing attention. It is up to the individual to critically assess their own results and, if necessary, seek expert advice.

Publication of your CIF in IUCr journals

A basic structural check has been run on your CIF. These basic checks will be run on all CIFs submitted for publication in IUCr journals (*Acta Crystallographica*, *Journal of Applied Crystallography*, *Journal of Synchrotron Radiation*); however, if you intend to submit to *Acta Crystallographica Section C* or *E* or *IUCrData*, you should make sure that full publication checks are run on the final version of your CIF prior to submission.

Publication of your CIF in other journals

Please refer to the *Notes for Authors* of the relevant journal for any special instructions relating to CIF submission.

PLATON version of 04/06/2025; check.def file version of 30/05/2025

Datablock MV022 - ellipsoid plot

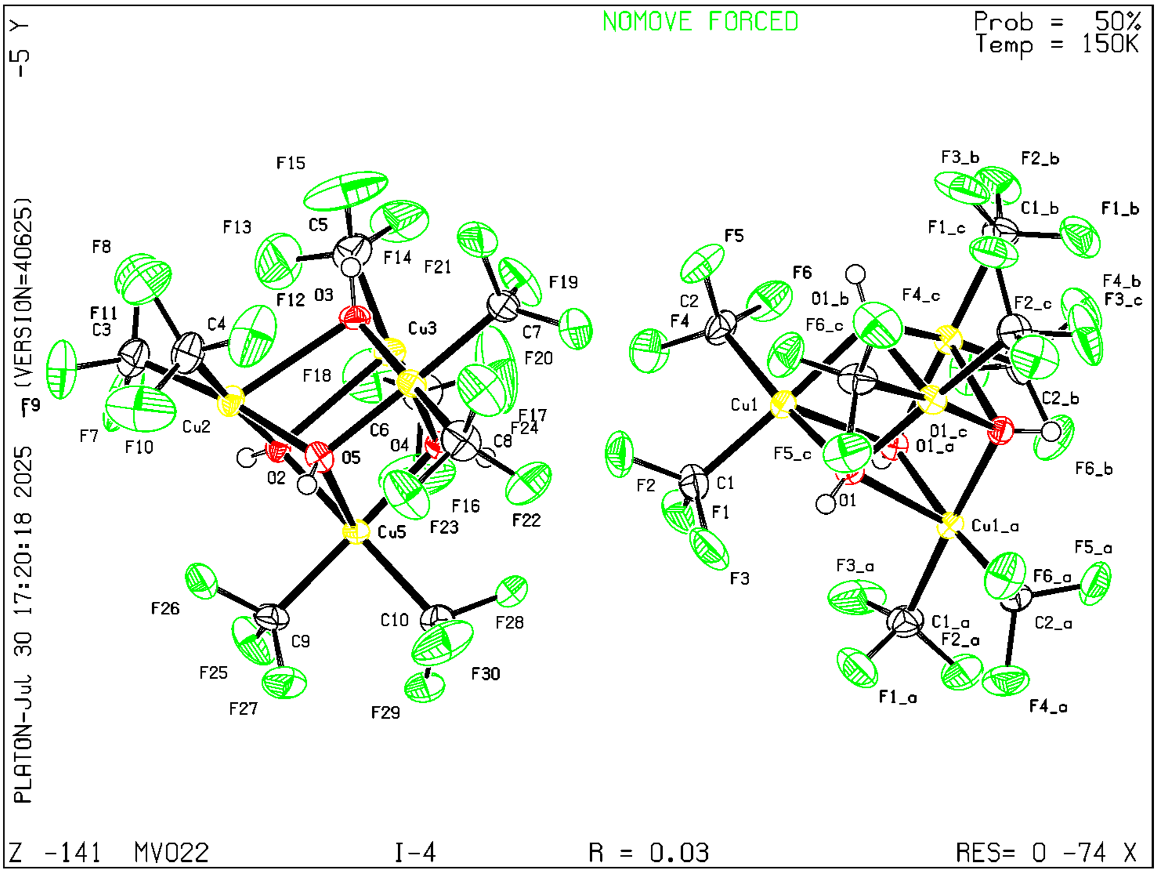

Supplement: Supplementary file 3 — Supporting file 3: chem70587‐sup‐0003‐SuppMat.zip [file CHEM-32-e03417-s003.zip › checkcif_3.pdf]
