# Supplementary material for: Unprecedented Formation of a Formally Cu(III) Trifluoromethyl Hydroxide Tetramer
Source: Chemistry. 2025 Dec 18;32(5):e03417. doi: 10.1002/chem.202503417 (PMC12865140; doi:10.1002/chem.202503417)

checkCIF/PLATON report

Structure factors have been supplied for datablock(s) MV025

THIS REPORT IS FOR GUIDANCE ONLY. IF USED AS PART OF A REVIEW PROCEDURE FOR PUBLICATION, IT SHOULD NOT REPLACE THE EXPERTISE OF AN EXPERIENCED CRYSTALLOGRAPHIC REFEREE.

No syntax errors found.   CIF dictionary   Interpreting this report

Datablock: MV025

```
Bond precision:   C-C = 0.0028 A          Wavelength=0.71073

Cell:            a=7.0179(3)       b=8.0222(4)       c=12.2963(6)
                 alpha=86.173(2)   beta=84.951(2)    gamma=86.220(2)
Temperature:     150 K

                  Calculated                   Reported
Volume            686.79(6)                    686.79(6)
Space group       P -1                         P -1
Hall group        -P 1                         -P 1
Moiety formula    C8 H6 Cu F6 N O3, C2 H3 N    C8 H6 Cu F6 N O3, C2 H3 N
Sum formula       C10 H9 Cu F6 N2 O3           C10 H9 Cu F6 N2 O3
Mr                382.74                       382.73
Dx,g cm-3         1.851                        1.851
Z                 2                            2
Mu (mm-1)         1.673                        1.673
F000              380.0                        380.0
F000'             380.93
h,k,lmax          8,9,14                       8,9,14
Nref              2531                         2529
Tmin,Tmax         0.523,0.809                  0.603,0.745
Tmin'             0.499

Correction method= # Reported T Limits: Tmin=0.603 Tmax=0.745
AbsCorr = MULTI-SCAN

Data completeness= 0.999          Theta(max)= 25.370

R(reflections)= 0.0221( 2330)                        wR2(reflections)=
                                                     0.0552( 2529)
S = 1.085                  Npar= 201
```

The following ALERTS were generated. Each ALERT has the format

test-name_ALERT_alert-type_alert-level.

Click on the hyperlinks for more details of the test.

Alert level G

```
PLAT007_ALERT_5_G Number of Unrefined Donor-H Atoms .............          2 Report
              H00A  H00B
PLAT154_ALERT_1_G The s.u.'s on the Cell Angles are Equal ..(Note)      0.002 Degree
PLAT232_ALERT_2_G Hirshfeld Test Diff (M-X)  Cu1      --C1       .        5.7 s.u.
PLAT242_ALERT_2_G Low    'MainMol' Ueq as Compared to Neighbors of         C1 Check
PLAT242_ALERT_2_G Low    'MainMol' Ueq as Compared to Neighbors of         C2 Check
PLAT720_ALERT_4_G Number of Unusual/Non-Standard Labels .........          3 Note
              O00A    H00A    H00B
PLAT794_ALERT_5_G Tentative Bond Valency for Cu1       (III)     .       2.98 Info
PLAT910_ALERT_3_G Missing FCF Reflection(s) Below Theta(Min)[Deg]=       2.55 Note
                0  0  1,
PLAT912_ALERT_4_G Missing # of FCF Reflections Above STh/L=  0.600          1 Note
PLAT969_ALERT_5_G The 'Henn et al.' R-Factor-gap value ..........      1.968 Note
              Predicted wR2: Based on SigI**2  2.80 or SHELX Weight  5.08
PLAT978_ALERT_2_G Number C-C Bonds with Positive Residual Density.          5 Info
```

0 **ALERT level A** = Most likely a serious problem - resolve or explain
0 **ALERT level B** = A potentially serious problem, consider carefully
0 **ALERT level C** = Check. Ensure it is not caused by an omission or oversight
11 **ALERT level G** = General information/check it is not something unexpected

1 ALERT type 1 CIF construction/syntax error, inconsistent or missing data
4 ALERT type 2 Indicator that the structure model may be wrong or deficient
1 ALERT type 3 Indicator that the structure quality may be low
2 ALERT type 4 Improvement, methodology, query or suggestion
3 ALERT type 5 Informative message, check

It is advisable to attempt to resolve as many as possible of the alerts in all categories. Often the minor alerts point to easily fixed oversights, errors and omissions in your CIF or refinement strategy, so attention to these fine details can be worthwhile. In order to resolve some of the more serious problems it may be necessary to carry out additional measurements or structure refinements. However, the purpose of your study may justify the reported deviations and the more serious of these should normally be commented upon in the discussion or experimental section of a paper or in the "special_details" fields of the CIF. checkCIF was carefully designed to identify outliers and unusual parameters, but every test has its limitations and alerts that are not important in a particular case may appear. Conversely, the absence of alerts does not guarantee there are no aspects of the results needing attention. It is up to the individual to critically assess their own results and, if necessary, seek expert advice.

Publication of your CIF in IUCr journals

A basic structural check has been run on your CIF. These basic checks will be run on all CIFs submitted for publication in IUCr journals (*Acta Crystallographica*, *Journal of Applied Crystallography*, *Journal of Synchrotron Radiation*); however, if you intend to submit to *Acta Crystallographica Section C* or *E* or *IUCrData*, you should make sure that full publication checks are run on the final version of your CIF prior to submission.

Publication of your CIF in other journals

Please refer to the *Notes for Authors* of the relevant journal for any special instructions relating to CIF submission.

PLATON version of 04/06/2025; check.def file version of 30/05/2025

Datablock MV025 - ellipsoid plot

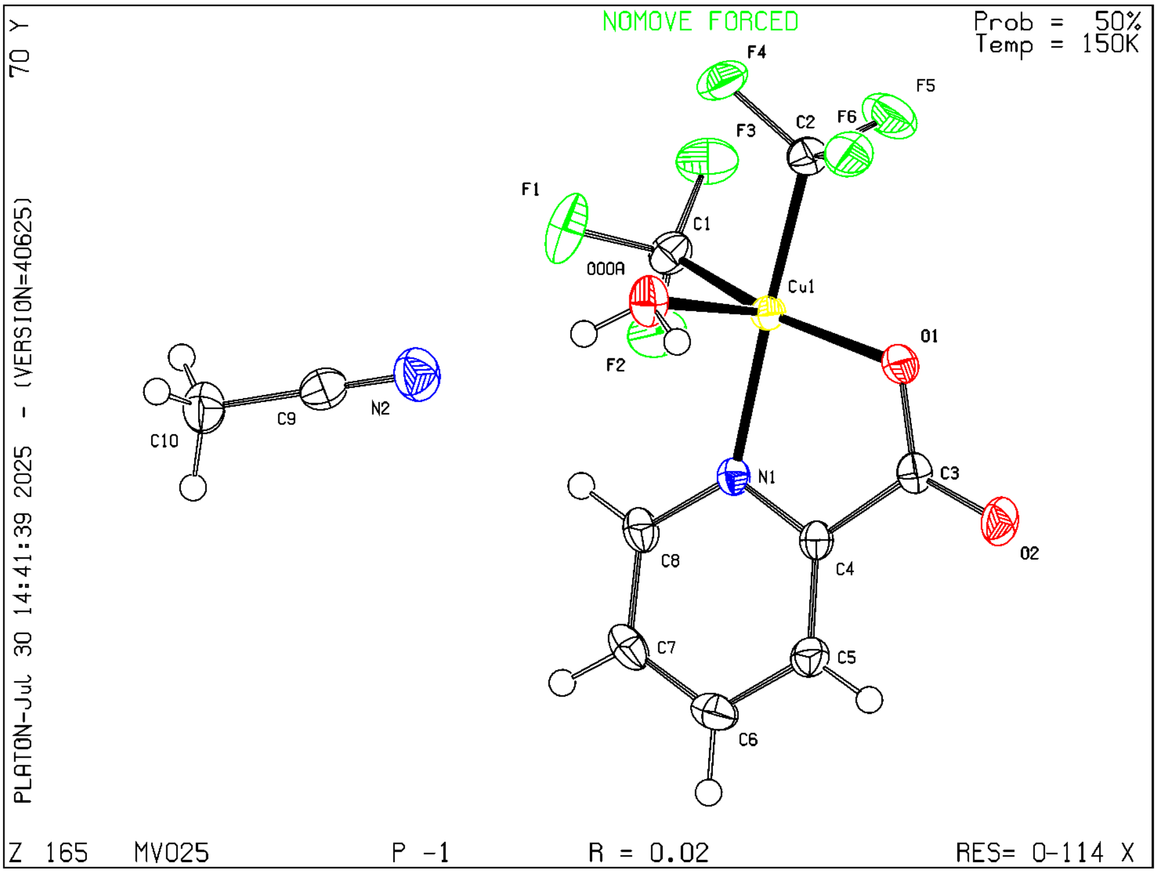

Supplement: Supplementary file 3 — Supporting file 3: chem70587‐sup‐0003‐SuppMat.zip [file CHEM-32-e03417-s003.zip › checkcif_4.pdf]
