# Supplementary material for: Unprecedented Formation of a Formally Cu(III) Trifluoromethyl Hydroxide Tetramer
Source: Chemistry. 2025 Dec 18;32(5):e03417. doi: 10.1002/chem.202503417 (PMC12865140; doi:10.1002/chem.202503417)

checkCIF/PLATON report

Structure factors have been supplied for datablock(s) MV021

THIS REPORT IS FOR GUIDANCE ONLY. IF USED AS PART OF A REVIEW PROCEDURE FOR PUBLICATION, IT SHOULD NOT REPLACE THE EXPERTISE OF AN EXPERIENCED CRYSTALLOGRAPHIC REFEREE.

No syntax errors found. CIF dictionary Interpreting this report

Datablock: MV021

Bond precision: C-C = 0.0029 A Wavelength=0.71073

Cell:	a=11.6062(4)	b=14.1352(5)	c=14.6945(6)
	alpha=90	beta=108.457(1)	gamma=90
Temperature:	150 K		

	Calculated	Reported
Volume	2286.72(15)	2286.72(15)
Space group	C 2/c	C2/c
Hall group	-C 2yc	-C 2yc
Moiety formula	C18 H12 Cu2 F12 N2 S2	2(C9 H6 Cu F6 N S)
Sum formula	C18 H12 Cu2 F12 N2 S2	C18 H12 Cu2 F12 N2 S2
Mr	675.52	675.50
Dx,g cm-3	1.962	1.962
Z	4	4
Mu (mm-1)	2.151	2.151
F000	1328.0	1328.0
F000'	1332.47	
h,k,lmax	13,16,17	13,16,17
Nref	2088	2086
Tmin,Tmax	0.469,0.542	0.611,0.745
Tmin'	0.362	

Correction method= # Reported T Limits: Tmin=0.611 Tmax=0.745
AbsCorr = MULTI-SCAN

Data completeness= 0.999 Theta(max)= 25.363

R(reflections)= 0.0217(1947) wR2(reflections)= 0.0563(2086)

S = 1.060 Npar= 230

The following ALERTS were generated. Each ALERT has the format
test-name_ALERT_alert-type_alert-level.
Click on the hyperlinks for more details of the test.

Alert level C

```
PLAT042_ALERT_1_C Calc. and Reported MoietyFormula Strings  Differ     Please Check
              Calc: C18 H12 Cu2 F12 N2 S2
              Rep.: 2(C9 H6 Cu F6 N S)
PLAT088_ALERT_3_C Poor Data / Parameter Ratio ...................       9.07 Note
```

Alert level G

```
PLAT002_ALERT_2_G Number of Distance or Angle Restraints on AtSite         17 Note
PLAT003_ALERT_2_G Number of Uiso or U(i,j)  Restrained non-H-Atoms         16 Report
PLAT007_ALERT_5_G Number of Unrefined Donor-H Atoms .............           1 Report
              H1
PLAT171_ALERT_4_G The CIF-Embedded .res File Contains EADP Records          1 Report
PLAT172_ALERT_4_G The CIF-Embedded .res File Contains DFIX Records          6 Report
PLAT176_ALERT_4_G The CIF-Embedded .res File Contains SADI Records         24 Report
PLAT186_ALERT_4_G The CIF-Embedded .res File Contains ISOR Records          1 Report
PLAT191_ALERT_3_G A Non-default SADI Restraint Value has been used     0.0300 Report
PLAT191_ALERT_3_G A Non-default SADI Restraint Value has been used     0.0400 Report
PLAT191_ALERT_3_G A Non-default SADI Restraint Value has been used     0.0300 Report
PLAT191_ALERT_3_G A Non-default SADI Restraint Value has been used     0.0400 Report
PLAT191_ALERT_3_G A Non-default SADI Restraint Value has been used     0.0400 Report
PLAT191_ALERT_3_G A Non-default SADI Restraint Value has been used     0.0400 Report
PLAT191_ALERT_3_G A Non-default SADI Restraint Value has been used     0.0400 Report
PLAT191_ALERT_3_G A Non-default SADI Restraint Value has been used     0.0400 Report
PLAT191_ALERT_3_G A Non-default SADI Restraint Value has been used     0.0400 Report
PLAT191_ALERT_3_G A Non-default SADI Restraint Value has been used     0.0400 Report
PLAT191_ALERT_3_G A Non-default SADI Restraint Value has been used     0.0400 Report
PLAT191_ALERT_3_G A Non-default SADI Restraint Value has been used     0.0400 Report
PLAT191_ALERT_3_G A Non-default SADI Restraint Value has been used     0.0400 Report
PLAT191_ALERT_3_G A Non-default SADI Restraint Value has been used     0.0400 Report
PLAT232_ALERT_2_G Hirshfeld Test Diff (M-X)  Cu1      --C3       .        6.9 s.u.
PLAT232_ALERT_2_G Hirshfeld Test Diff (M-X)  Cu1      --C1B      .        5.9 s.u.
PLAT301_ALERT_3_G Main Residue  Disorder ..............(Resd    1)        44% Note
PLAT811_ALERT_5_G No ADDSYM Analysis: Too Many Excluded Atoms ....          ! Info
PLAT860_ALERT_3_G Number of Least-Squares Restraints ............         152 Note
PLAT910_ALERT_3_G Missing FCF Reflection(s) Below Theta(Min)[Deg]=       2.43 Note
                1  1  0,
PLAT933_ALERT_2_G Number of HKL-OMIT Records in Embedded .res File          1 Note
                1  1  0,
PLAT969_ALERT_5_G The 'Henn et al.' R-Factor-gap value ..........       2.183 Note
              Predicted wR2: Based on SigI**2  2.58 or SHELX Weight  5.31
PLAT978_ALERT_2_G Number C-C Bonds with Positive Residual Density.          6 Info
```

0 **ALERT level A** = Most likely a serious problem - resolve or explain
0 **ALERT level B** = A potentially serious problem, consider carefully
2 **ALERT level C** = Check. Ensure it is not caused by an omission or oversight
30 **ALERT level G** = General information/check it is not something unexpected

1 ALERT type 1 CIF construction/syntax error, inconsistent or missing data

```
 6 ALERT type 2 Indicator that the structure model may be wrong or deficient
18 ALERT type 3 Indicator that the structure quality may be low
 4 ALERT type 4 Improvement, methodology, query or suggestion
 3 ALERT type 5 Informative message, check
```

It is advisable to attempt to resolve as many as possible of the alerts in all categories. Often the minor alerts point to easily fixed oversights, errors and omissions in your CIF or refinement strategy, so attention to these fine details can be worthwhile. In order to resolve some of the more serious problems it may be necessary to carry out additional measurements or structure refinements. However, the purpose of your study may justify the reported deviations and the more serious of these should normally be commented upon in the discussion or experimental section of a paper or in the "special_details" fields of the CIF. checkCIF was carefully designed to identify outliers and unusual parameters, but every test has its limitations and alerts that are not important in a particular case may appear. Conversely, the absence of alerts does not guarantee there are no aspects of the results needing attention. It is up to the individual to critically assess their own results and, if necessary, seek expert advice.

Publication of your CIF in IUCr journals

A basic structural check has been run on your CIF. These basic checks will be run on all CIFs submitted for publication in IUCr journals (*Acta Crystallographica*, *Journal of Applied Crystallography*, *Journal of Synchrotron Radiation*); however, if you intend to submit to *Acta Crystallographica Section C* or *E* or *IUCrData*, you should make sure that full publication checks are run on the final version of your CIF prior to submission.

Publication of your CIF in other journals

Please refer to the *Notes for Authors* of the relevant journal for any special instructions relating to CIF submission.

PLATON version of 04/06/2025; check.def file version of 30/05/2025

Datablock MV021 - ellipsoid plot

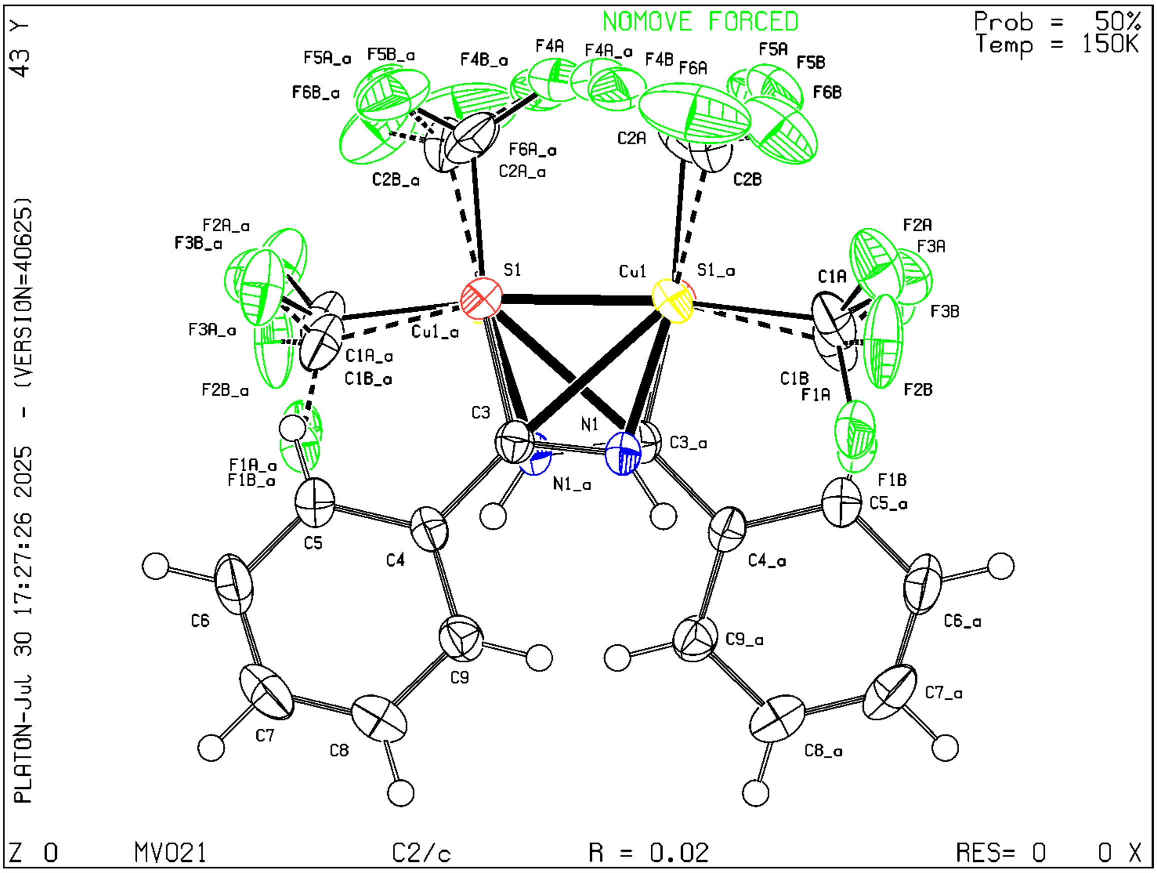

Supplement: Supplementary file 3 — Supporting file 3: chem70587‐sup‐0003‐SuppMat.zip [file CHEM-32-e03417-s003.zip › checkcif_8.pdf]
