# Supplementary material for: Unprecedented Formation of a Formally Cu(III) Trifluoromethyl Hydroxide Tetramer
Source: Chemistry. 2025 Dec 18;32(5):e03417. doi: 10.1002/chem.202503417 (PMC12865140; doi:10.1002/chem.202503417)

checkCIF/PLATON report

Structure factors have been supplied for datablock(s) MV026

THIS REPORT IS FOR GUIDANCE ONLY. IF USED AS PART OF A REVIEW PROCEDURE FOR PUBLICATION, IT SHOULD NOT REPLACE THE EXPERTISE OF AN EXPERIENCED CRYSTALLOGRAPHIC REFEREE.

No syntax errors found. CIF dictionary Interpreting this report

Datablock: MV026

Bond precision:	C-C = 0.0160 A		Wavelength=0.71073
Cell:	a=7.5786(15)	b=12.770(3)	c=9.1246(17)
	alpha=90	beta=91.771(7)	gamma=90
Temperature:	150 K		

	Calculated	Reported
Volume	882.7(3)	882.7(3)
Space group	P 21/c	P 1 21/c 1
Hall group	-P 2ybc	-P 2ybc
Moiety formula	C4 H5 Cu F6 O3, H2 O	C4 H5 Cu F6 O3, H2 O
Sum formula	C4 H7 Cu F6 O4	C4 H7 Cu F6 O4
Mr	296.65	296.64
Dx,g cm-3	2.232	2.232
Z	4	4
Mu (mm-1)	2.569	2.569
F000	584.0	584.0
F000’	585.84	
h,k,lmax	9,15,11	9,15,11
Nref	1620	1602
Tmin,Tmax	0.764,0.842	0.492,0.745
Tmin’	0.764	

Correction method= # Reported T Limits: Tmin=0.492 Tmax=0.745
AbsCorr = MULTI-SCAN

Data completeness= 0.989 Theta(max)= 25.409

R(reflections)= 0.0792(1226) wR2(reflections)= 0.1808(1602)

S = 1.171 Npar= 147

```
The following ALERTS were generated. Each ALERT has the format
       test-name_ALERT_alert-type_alert-level.
Click on the hyperlinks for more details of the test.
```

Alert level B

```
PLAT341_ALERT_3_B Low Bond Precision on  C-C Bonds ..............      0.016 Ang.
```

Author Response: The low bond precision is due to a poor data/parameter ratio resulting from twinning.

```
PLAT417_ALERT_2_B Short Inter D-H..H-D       H3B      ..H4E      .       2.06 Ang.
                                                      x,y,z  =      1_555 Check
```

Author Response: A short distance is observed between two hydrogen atoms due to intermolecular hydrogen bonding between two polymeric chains. The hydrogen atom H3B of the aqua ligand forms a hydrogen bond with O4 of the free water molecule, whose hydrogen atom H4E in turn forms a hydrogen bond with an oxygen atom of the acetate ligand. Altogether, the free water molecule participates in three hydrogen bonds, resulting in a short H-H contact between the aqua ligand and the free water molecule.

Alert level C

```
PLAT906_ALERT_3_C Large K Value in the Analysis of Variance ......      3.736 Check
PLAT911_ALERT_3_C Missing FCF Refl Between Thmin & STh/L=    0.600         13 Report
                1  5  1,  -2  0  2,  -1  0  2,   1  5  3,  -2  0  4,   0  1  7,
                0  0  8,   0  1  8,   0  2  8,   4  4  8,   0  2  9,   1  3  9,
                0  2 10,
```

Alert level G

```
PLAT002_ALERT_2_G Number of Distance or Angle Restraints on AtSite          3 Note
PLAT004_ALERT_5_G Polymeric Structure Found with Maximum Dimension          1 Info
PLAT007_ALERT_5_G Number of Unrefined Donor-H Atoms .............          2 Report
              H3A   H3B
PLAT083_ALERT_2_G SHELXL Second Parameter in WGHT  Unusually Large      19.62 Why ?
PLAT172_ALERT_4_G The CIF-Embedded .res File Contains DFIX Records          2 Report
PLAT242_ALERT_2_G Low    'MainMol' Ueq as Compared to Neighbors of         C2 Check
PLAT794_ALERT_5_G Tentative Bond Valency for Cu1       (III)     .       2.85 Info
PLAT860_ALERT_3_G Number of Least-Squares Restraints ............          3 Note
PLAT870_ALERT_4_G ALERTS Related to Twinning Effects Suppressed ..          ! Info
PLAT912_ALERT_4_G Missing # of FCF Reflections Above STh/L=  0.600          5 Note
PLAT933_ALERT_2_G Number of HKL-OMIT Records in Embedded .res File          2 Note
                1  5  1,   1  5  3,
PLAT955_ALERT_1_G Reported (CIF) and Actual (FCF) Lmax Differ by .          1 Units
PLAT969_ALERT_5_G The 'Henn et al.' R-Factor-gap value ..........      3.330 Note
              Predicted wR2: Based on SigI**2  5.43 or SHELX Weight 15.44
```

```
 0 ALERT level A = Most likely a serious problem - resolve or explain
 2 ALERT level B = A potentially serious problem, consider carefully
 2 ALERT level C = Check. Ensure it is not caused by an omission or oversight
13 ALERT level G = General information/check it is not something unexpected

 1 ALERT type 1 CIF construction/syntax error, inconsistent or missing data
 5 ALERT type 2 Indicator that the structure model may be wrong or deficient
 4 ALERT type 3 Indicator that the structure quality may be low
 3 ALERT type 4 Improvement, methodology, query or suggestion
 4 ALERT type 5 Informative message, check
```

It is advisable to attempt to resolve as many as possible of the alerts in all categories. Often the minor alerts point to easily fixed oversights, errors and omissions in your CIF or refinement strategy, so attention to these fine details can be worthwhile. In order to resolve some of the more serious problems it may be necessary to carry out additional measurements or structure refinements. However, the purpose of your study may justify the reported deviations and the more serious of these should normally be commented upon in the discussion or experimental section of a paper or in the "special_details" fields of the CIF. checkCIF was carefully designed to identify outliers and unusual parameters, but every test has its limitations and alerts that are not important in a particular case may appear. Conversely, the absence of alerts does not guarantee there are no aspects of the results needing attention. It is up to the individual to critically assess their own results and, if necessary, seek expert advice.

Publication of your CIF in IUCr journals

A basic structural check has been run on your CIF. These basic checks will be run on all CIFs submitted for publication in IUCr journals (*Acta Crystallographica*, *Journal of Applied Crystallography*, *Journal of Synchrotron Radiation*); however, if you intend to submit to *Acta Crystallographica Section C* or *E* or *IUCrData*, you should make sure that full publication checks are run on the final version of your CIF prior to submission.

Publication of your CIF in other journals

Please refer to the *Notes for Authors* of the relevant journal for any special instructions relating to CIF submission.

PLATON version of 04/06/2025; check.def file version of 30/05/2025

Datablock MV026 - ellipsoid plot

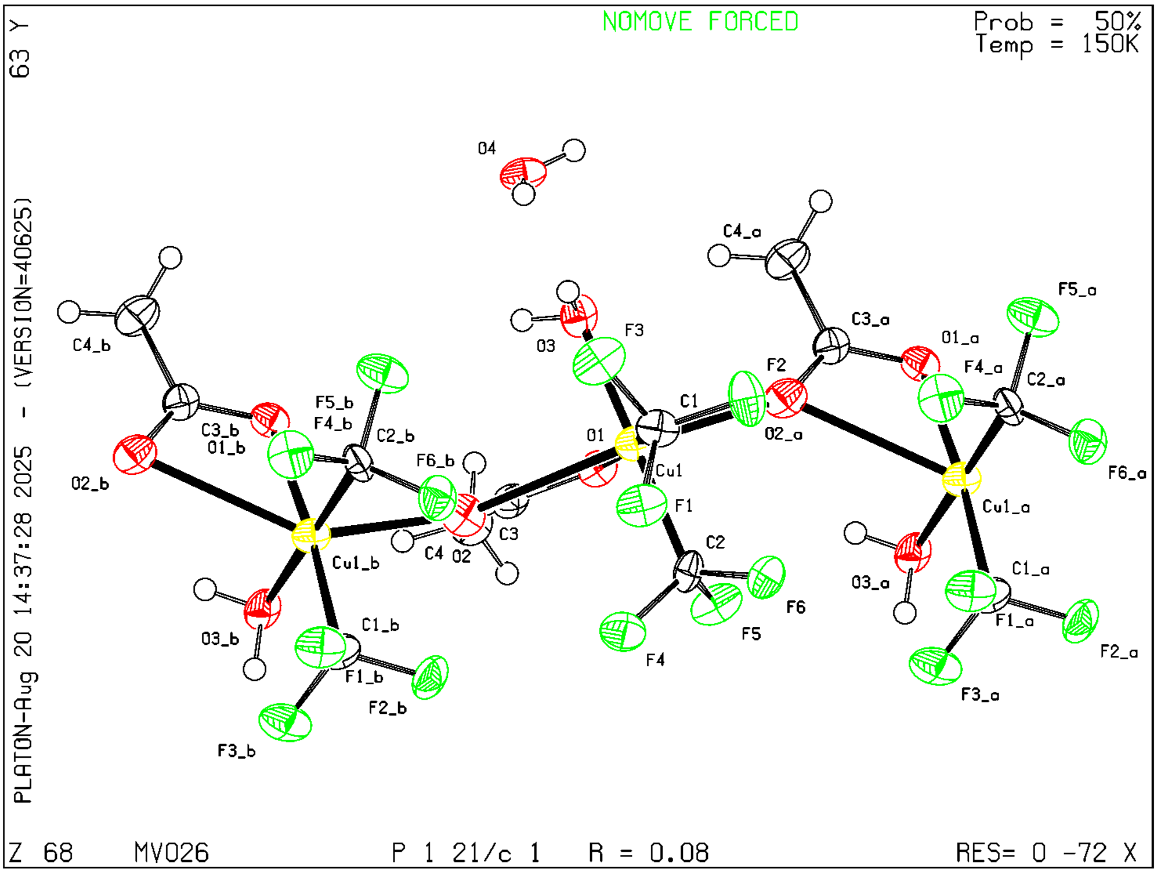

Supplement: Supplementary file 3 — Supporting file 3: chem70587‐sup‐0003‐SuppMat.zip [file checkcif_5.pdf › checkcif_5.pdf]
